# Pulmonopoly: A Game-Based Approach to Teach and Reinforce Basic Concepts of Pulmonary Medicine to Medical Students

**DOI:** 10.15766/mep_2374-8265.11493

**Published:** 2025-02-21

**Authors:** Michael Dong, James Uricheck, Urvashi Vaid

**Affiliations:** 1 Fellow in Pulmonary and Critical Care Medicine, Department of Pulmonary and Critical Care, Thomas Jefferson University Hospital; 2 Attending Physician, Department of Pulmonary and Critical Care, Kaiser Permanente Santa Rosa; 3 Associate Professor of Medicine, Department of Pulmonary and Critical Care, Sidney Kimmel Medical College at Thomas Jefferson University

**Keywords:** Physiology, Internal Medicine, Pulmonary Medicine, Games

## Abstract

**Introduction:**

Pulmonopoly is an innovative active-learning medical board game addressses core pulmonary concepts in an engaging, competitive style. The strength of Pulmonopoly lies in its ability to combine ease of setup and play with comprehensive content coverage that comprises a significant proportion of national board examinations, offering a gamification experience that can be readily applied in different settings and modified for learner levels.

**Methods:**

We created an instructional game, Pulmonopoly, based on pulmonary content delivered to preclinical medical students and required for national board examinations. We invited first-year medical students to participate in 60-minute small-group sessions. Content addressed pulmonary subject areas of anatomy and pharmacology, physiology, pathophysiology, and more advanced modifier questions. Correct answers were rewarded properties, and victory was achieved when all properties of a neighborhood were collected. Students completed pre- and postintervention surveys with 5-point Likert-scale responses about their experience, understanding of core pulmonary concepts, and whether playing Pulmonopoly reinforced these concepts.

**Results:**

Fifty-six first-year medical students participated in the game sessions. The postintervention survey (*N* = 51), when compared to the preintervention survey (*N* = 56), demonstrated that students’ understanding of 11 core pulmonary concepts was reinforced after playing the board game (average Likert survey scores improved from 3.1 to 4.4). The surveys also provided positive narrative feedback on student engagement and satisfaction.

**Discussion:**

Pulmonopoly is an engaging game-based approach to help improve and reinforce core pulmonary concepts for medical students. The game requires minimal setup and preparation and is easily replicable at other institutions.

## Educational Objectives

By the end of this activity, medical students will be able to:

1. Describe the normal pulmonary and thoracic anatomy.
2. Describe and apply the appropriate treatment for obstructive airway diseases and smoking cessation, with a focus on the use of inhalers and the rationale behind their selection.
3. Identify and explain the physiology and pathophysiology of acid-base disorders, obstructive and restrictive lung diseases, and ventilation/perfusion mismatch.
4. Apply knowledge of flow volume loops in pulmonary function testing to diagnose obstructive and restrictive lung diseases.

## Introduction

In recent years, there has been a rise in the utilization of board games as a nontraditional educational vehicle to increase the engagement of medical students.^1,2^ The gamification of medical education is an effective approach to teaching complex topics, and its interactive nature increases learner satisfaction.^3–5^ Previously published medical education games, such as The Bloody Board Game, Candy Gland, and LiverLand, have demonstrated effectiveness in teaching in their respective subject areas.^1,2,6^ In our review of *MedEdPORTAL*, as well as a general literature search, we identified three educational games focused on pulmonary medicine designed for classroom use.^7–9^ However, these games required a significant investment of time and resources, offered limited coverage of pulmonary concepts, and lacked broad generalizability.

Pulmonary medicine can be a challenging subject for medical students because of the breadth of topics: respiratory physiology and anatomy, pathophysiology of acid/base problems, ventilation/perfusion mismatch, obstructive and restrictive lung diseases, and inhaler pharmacology, to name a few. Pulmonary medicine makes up 9% of the American Board of Internal Medicine's National Certification Exam^10^ and up to 10%-15% of the USMLE Step 1 examination (categorized as Respiratory & Renal/Urinary Systems).^11^ In a needs assessment of our preclinical curriculum, we noticed that knowledge from these subject areas can be challenging for learners to apply in case-based learning sessions and summative assessments.

We sought to address these learning challenges by developing an active-learning education innovation that diverges from the conventional lecture-based format. In comparison to previous approaches to pulmonary medicine gamification, the strength of Pulmonopoly lies in its ability to combine ease of setup and play with comprehensive content coverage that comprises a significant proportion of national board examinations, offering a gamification experience that can be readily applied in different settings and modified for learner levels.

We aim to demonstrate that the Pulmonopoly medical board game can increase medical student engagement and enjoyment while effectively reinforcing and teaching core concepts in pulmonary medicine.

## Methods

Sidney Kimmel Medical College (SKMC) at Thomas Jefferson University offers its students a longitudinally integrated preclinical systems-based curriculum. Pulmonary medicine is one of the systems that students study halfway through their first year of medical school. We administered Pulmonopoly to student volunteers as a supplement to their didactic and team-based instruction. Participation was voluntary, and there was no incentive. The innovation was announced at the beginning of the students’ pulmonary block and followed up with an invitation to participate via email once the students had completed their routine curricular instruction in the content areas that we had used to create questions for the game.

A needs assessment was conducted through qualitative analysis of student evaluations and multiple-choice exam performance from the preceding 5 years of the pulmonary block in the first-year SKMC curriculum. The data suggested that pulmonary physiology, acid-base physiology, and pulmonary pharmacology were particularly challenging for students on board-style multiple-choice question (MCQ) assessments. Percent correct scores on MCQs covering this material in the pulmonary end-of-block exam were consistently lower than those for other pulmonary topics, a trend further supported by student comments and curriculum evaluation responses. Using this feedback, the authors curated Pulmonopoly's question content based on the SKMC pulmonary block's learning objectives and lecture materials. The final content was reviewed by one of the authors, who serves as the director of the preclinical curriculum, ensuring alignment with educational standards and curricular goals.

Pulmonopoly is a multiplayer game board created by the authors and styled after the classic Hasbro game Monopoly. Of note, the game mechanics of Pulmonopoly are unique and distinct from Monopoly and do not incorporate the use of houses, hotels, or currency as in the Hasbro game.

Groups of four to eight students were divided into four teams in a large classroom and used the printed Pulmonopoly gameboard (Appendix A), player pieces, dice, and four printed card decks (Appendix B) to play the game. Full instructions on gameplay were discussed with the students.

Briefly, a team would roll the dice and then move their game piece according to the rolled number. When landing on a property space, the team would draw a corresponding question card and attempt to answer it; a correct answer allowed the team to acquire the property, while an incorrect answer ended their turn. The moderator, equipped with a question bank and answer key—including explanations—would provide the correct answer and additional salient teaching points after each question. If a team landed on a Modifier space, they drew a card from the Modifier deck and attempted to answer it; correctly answering allowed them to retain the Modifier card, which could be used strategically later in the game. Modifier cards offered various functions: using one could adjust a dice roll by up to two, using two could force a property trade with another team, and using three could secure any property at the end of the turn. The objective was to acquire all properties within a single color-coordinated neighborhood to win the game.

We created 200 question cards split into four decks corresponding to core pulmonary subject areas: anatomy and pharmacology, physiology, pathophysiology, and modifier questions. Modifier questions were drawn from more advanced content domains related to pulmonary medicine that require a higher order of clinical reasoning. Most questions were multiple choice with a single best answer, with a few questions in a short-answer format. We designed the questions on the cards to reinforce key pulmonary content previously covered in class materials during the pulmonary block. The answer key that provided correct answers with corresponding explanations was included.

We also created property cards that were given to the teams for answering questions correctly. These game cards could be printed on standard printer paper and cut to size, with 16 cards per page (Appendix B contains question cards and Appendix C contains property cards). The 16 × 16-inch gameboard could be printed on poster paper or created by simply printing and combining four sheets of standard printer paper (Appendix A).

Four internal medicine residents volunteered as moderators for the game, and each was provided with the question bank, answer key (Appendix D), and rules (Appendix E). The medical students were instructed on gameplay rules before starting the activity, and printed gameplay rules were available to them. In all of our sessions, an instructor (a pulmonary fellow) was present to settle disputes and answer any questions arising from the discussion over the correct answer. The moderators required no prerequisite training. The instructor had prerequisite knowledge about the gameplay and rules and was knowledgeable about the pulmonary concepts tested in the game. Moderators and instructors are not required, as the learners can reference the comprehensive answer key that is provided; however, we utilized volunteers in these roles to improve the pace of the game and enhance the educational experience. The moderator had the question bank and answer key, which includes explanations for the majority of the answers. After the team answered, the moderator read the correct answer and explanation to the teams. They also added salient teaching points. We recommend senior medical students or residents for the moderator role, if available.

At the beginning of each session, individual students completed a preintervention survey (Appendix F) with Likert-scale responses about their understanding of 11 core concepts involving obstructive and restrictive lung disease, acid-base and ventilation/perfusion problems, inhaler pharmacology, smoking cessation pharmacology, and pulmonary anatomy. At the end of the session, a postintervention survey (Appendix F) with a Likert-scale format assessed whether playing Pulmonopoly was enjoyable and engaging and reinforced the aforementioned concepts. We used the Kirkpatrick model to evaluate the effectiveness of Pulmonopoly as a learning tool. This educational innovation was designed to assess Kirkpatrick levels 1 and 2 (i.e., reaction and learning, respectively).^12^

The Thomas Jefferson University Hospital Institutional Review Board deemed further review of this project not necessary (#1002848).

## Results

A total of 56 out of 272 first-year medical students enrolled in SKMC participated in the innovation two-thirds of the way through their pulmonary medicine block in January 2023. Of the 56 participants, 56 (100%) completed the preintervention survey, and 51 (91%) completed the postintervention survey.

The preintervention survey captured students’ understanding of core concepts of obstructive and restrictive lung disease, acid-base and ventilation/perfusion problems, inhaler pharmacology, smoking cessation pharmacology, and pulmonary anatomy at baseline. The postintervention survey evaluated whether playing Pulmonopoly reinforced the student's understanding of these subject areas. The average score on the preintervention survey was 3.1 out of 5 on a 5-point Likert scale (1 = *strongly disagree*, 2 = *disagree*, 3 = *neutral*, 4 = *agree*, 5 = *strongly agree*). This was consistent with a *neutral* response to the statement that the learner had a good understanding of each of the 11 pulmonary medicine core concepts. The average score on the postintervention survey was 4.4, consistent with an *agree*/*strongly agree* response to the questions related to Pulmonopoly, reinforcing the core pulmonary concepts. In the preintervention survey, the areas where students felt their understanding was weakest included acid-base physiology, acid-base pathophysiology, inhaler pharmacology, and smoking cessation. A comparison of the pre- and postintervention surveys revealed that these four concepts had the most significant increases in Likert scores after the intervention. Every question in the postintervention survey involving a pulmonary medicine concept increased by at least 1 point.

**Figure 1.**
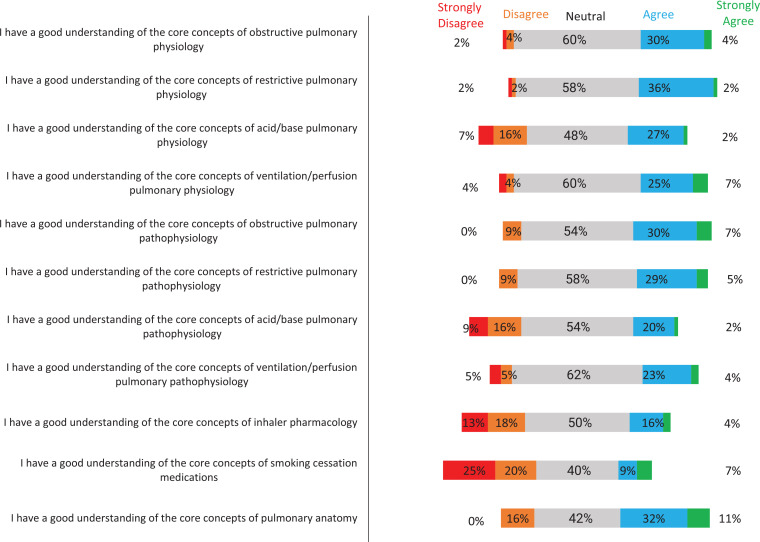
Horizontal diverging bar chart displaying the distribution of medical student responses to each of the statements on the preintervention survey rated on a 5-point Likert scale (1 = *strongly disagree*, 5 = *strongly agree*).

**Figure 2.**
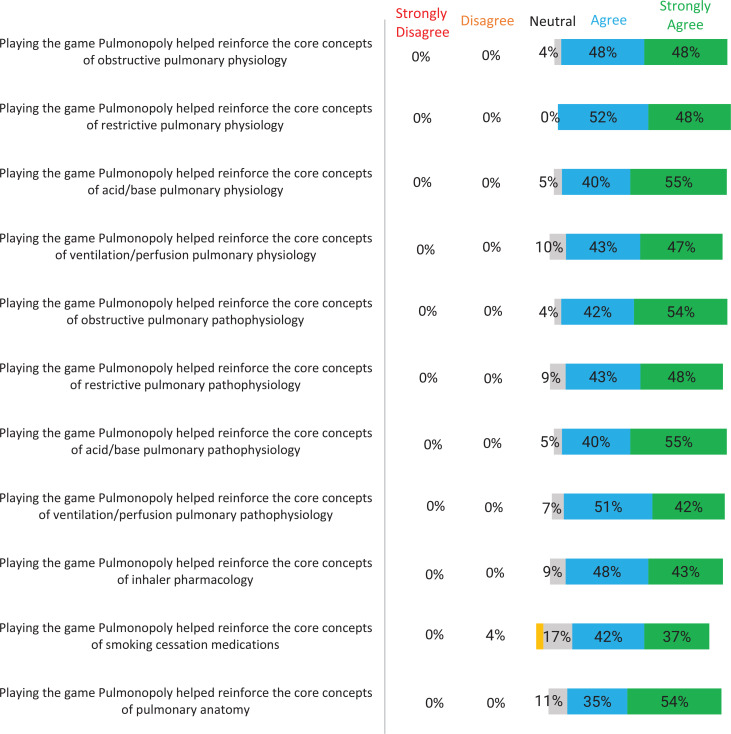
Horizontal diverging bar chart displaying the distribution of medical student responses to each of the statements on the postintervention survey rated on a 5-point Likert scale (1 = *strongly disagree*, 5 = *strongly agree*).

Furthermore, on the preintervention survey, the percentage of students who *agreed* or *strongly agreed* that they had a good understanding of each core concept ranged from 16% to 43%. The majority of responses were *strongly disagree/disagree* or *neutral*. In the postintervention survey, the percentage of students who *agreed* or *strongly agreed* that Pulmonopoly reinforced the 11 core concepts ranged from 79% to 100% (Figures 1 and 2).

Using the Kirkpatrick model to evaluate the effectiveness of Pulmonopoly as a learning tool, we assessed learners at levels 1 and 2 (reaction and learning, respectively).^12^ The game also elicited unsolicited positive feedback. Examples are included below. Several students also wished to attend multiple game sessions (though they were only allowed to fill out the surveys in the first session they attended).

• “It was a really engaging way to do practice questions, and I hope this is done in future years as well!”
• “Is there any way that Pulmonopoly can be incorporated into exam reviews? This would be a really useful tool to study for the exam.”
• “Can I borrow Pulmonopoly or sign it out at the library?”
• “I really enjoyed Pulmonopoly!”
• “Will there be more sessions of Pulmonopoly?”
• “We purposely avoided gaining that property so the game wouldn't end and we could keep playing.”

## Discussion

Our board game, Pulmonopoly, is a pedagogical innovation that strengthens core concepts in pulmonary medicine and generates enthusiasm and collaboration when played by medical students. Our postintervention survey showed that students felt the core pulmonary concepts were reinforced significantly.

The primary goal of the curriculum is to reinforce the learning objectives by encouraging students to apply foundational concepts through recall and repetition and use these concepts to advance clinical reasoning with question-based learning. The game content was developed based on a needs assessment of prior student performance on multiple-choice exams and aligned with the learning objectives from the pulmonary block preclinical lectures. Therefore, one intended outcome is improved student performance on standardized tests. Additionally, the game can foster clinically relevant discussions through interactive gameplay moderated by resident physicians (when moderators are present). This setting allows learners to contextualize questions within real-world clinical scenarios, enhancing their understanding of patient conditions and treatment approaches as they prepare for clinical rotations.

This innovation uncovered considerable differences in survey scores postintervention in the students understanding of concepts of acid-base physiology and pathophysiology, smoking cessation pharmacology, and inhaler pharmacology. Notably, these were also the four lowest scoring concepts identified on the preintervention survey, which suggests that before the intervention, these were the areas in which the students felt their comprehension was the weakest. Pulmonopoly was particularly successful at teaching and reinforcing these subjects—subjects that we postulate may have been more arduous for learners to grasp in a conventional classroom setting. As supported by prior publications, a game-based approach can make learning traditionally challenging topics both practical and compelling.^13–15^

Pulmonopoly is a low-cost learning innovation with an easy setup and requires minimal preparation time. The resources required to run the game are all available for free in the appendices and can be printed. Moderators require no prerequisite training. The game sessions are replicable, the format fosters a competitive spirit, and the interactive discussion arising from questions solidifies learning. Each game requires only 1 hour of in-person curricular time but can be played multiple times to expose students to the breadth of questions available. Prior medical education board games, such as LiverLand, The Bloody Board Game, Candy Gland, Learning to Beat the Shock Clock, and L&D in the ED, similarly demonstrate effectiveness in conveying knowledge and in participant satisfaction.^1,2,6,16,17^ Educational innovations using board games such as ours share a common limitation in that they demonstrate the ability to facilitate learning and enthusiasm (Kirkpatrick levels 1 and 2), but they do not measure changes in behavior or performance in examinations or the clinical space (Kirkpatrick levels 3 and 4).^18^

In the assessment of Kirkpatrick level 2 knowledge acquisition, a limitation we encountered was the lack of formal pre/post knowledge assessment. As a future direction, an analysis of participant versus nonparticipant test scores in the pulmonary system examinations would provide an objective measure assessing whether our innovation achieved a Kirkpatrick level 2 change in student learning and knowledge. Another limitation is that due to the large volume of content and the limited time available, learners may not fully cover or engage with all intended learning objectives within a single 1-hour session. With only a portion of the 200 questions addressed in the time frame, some critical topics or concepts may be missed, leading to variability in what students take away from the experience. As such, this game should be used to supplement an existing curriculum rather than replacing it. Its value as a learning tool rises if students have a background understanding of basic pulmonary concepts.

Several learners expressed the desire to return to play multiple sessions so they could have the opportunity to review more questions. This raises the opportunity to design this innovation as a future self-study tool. A potential limitation is the challenge of implementing board games in virtual- and/or hybrid-classroom settings. Our educational session was conducted in person; however, Pulmonopoly game mechanics would be conducive for virtual play. For example, Zoom breakout rooms could be used, with the moderator in charge of the physical board and cards.

We found that having interns and residents as moderators was particularly helpful in adding clinical context and connections to the answers, as well as guiding discussions between the learners. While the answer key included in the game is sufficient for the game to be played with minimal moderator involvement, the moderators have the impact of adding to the depth of discussion and learning.

Aside from board games, other gamification-based strategies have been highlighted in the medical education literature. One example uses escape rooms to simulate medical scenarios and foster the use of team dynamics.^19,20^ The interactive situations presented in these games stimulate higher-order critical thinking and enhance team collaboration. They also facilitate evaluation at Kirkpatrick level 3 by measuring changes in behavior. However, a disadvantage is that these scenario-based games cannot be readily reproduced and translated to other institutions and may require significant specialized setup and preparation. In contrast, Pulmonopoly can be easily implemented at other institutions and for various levels of learners. Each game set includes all the necessary components that can be printed to play immediately, and the game can be adapted to varying group sizes by either increasing the number of game boards or having pairs of players per team.

We hope to amplify the implementation of Pulmonopoly in our medical school curriculum as a reinforcement and learning tool for preclinical and third-year medical students on their internal medicine clerkships. In the future, we also aim to create expanded question banks targeted at the knowledge level required of internal medicine residents and pulmonary and critical care fellows, which we hope to share and collaborate with other institutions.

## Appendices


Pulmonopoly Board.pdfQuestion Cards.docxProperty Cards, Modifier Cards, and Player Pieces.pdfQuestion and Answer Key.docxGame Rules.docxPre- and Postintervention Surveys.docx
*All appendices are peer reviewed as integral parts of the Original Publication.*
