# Supplementary material for: Pulmonopoly: A Game-Based Approach to Teach and Reinforce Basic Concepts of Pulmonary Medicine to Medical Students
Source: MedEdPORTAL. 2025 Feb 21;21:11493. doi: 10.15766/mep_2374-8265.11493 (PMC11842520; doi:10.15766/mep_2374-8265.11493)
Supplement: Supplementary file 1 — Pulmonopoly Board.pdfQuestion Cards.docxProperty Cards, Modifier Cards, and Player Pieces.pdfQuestion and Answer Key.docxGame Rules.docxPre- and Postintervention Surveys.docx [file mep_2374-8265.11493-s001.zip › A. Pulmonopoly Board.pdf]

Asthma Attack!

False Alarm

Bronchial Boulevard
Physiology

Attempt a Modifier Draw

Phrenic Parkway
Anatomy & Pharmacology

Spacer Station
Patho-physiology

Modifier Draw

Obstruction Overpass
Patho-physiology

Diaphragmatic Diner
Anatomy & Pharmacology

Attempt a Modifier Draw

Auscultation Alley
Physiology

Attempt a

Modifie

PULMON

Attempt a

Alveolar Avenue

Physiology

SABA Square

Anatomy & Pharmacology

Attempt a Modifier Draw

Inhaler Island

Patho-physiology

Breath of Fresh Air: Draw One Free Modifier Card

Pleural Place

Physiology

Lake LABA

Anatomy & Pharmacology

Attempt a Modifier Draw

Pneumonia Pnational Park

Patho-physiology

Attempt a

er Cards

NOPOLY

Modifier Draw

Dyspnea Drive
Patho-physiology

Mucolytic Mountain
Anatomy & Pharmacology

Attempt a Modifier Draw

The Vagus Strip
Physiology

Anatomy & Pharmacology

Physiology

Refill Prescription: Draw One Free Modifier Card As You Pass

The Bronchs
Patho-physiology

Tracheal Terrace
Anatomy & Pharmacology

Attempt a Modifier Draw

Ventilator Vista
Physiology

Roll Again!

Modifier Draw

Roll Again!

CPET Coliseum

Physiology

Attempt a Modifier Draw

PFT Palace

Patho-physiology

Patho-physiology

Lost Your Inhaler! Go To Asthma Attack

Chronic Cough Canyon

Physiology

Attempt a Modifier Draw

The Sputum Sea

Patho-physiology

Attempt a Modifier Draw
