# Supplementary material for: Pulmonopoly: A Game-Based Approach to Teach and Reinforce Basic Concepts of Pulmonary Medicine to Medical Students
Source: MedEdPORTAL. 2025 Feb 21;21:11493. doi: 10.15766/mep_2374-8265.11493 (PMC11842520; doi:10.15766/mep_2374-8265.11493)
Supplement: Supplementary file 1 — Pulmonopoly Board.pdfQuestion Cards.docxProperty Cards, Modifier Cards, and Player Pieces.pdfQuestion and Answer Key.docxGame Rules.docxPre- and Postintervention Surveys.docx [file mep_2374-8265.11493-s001.zip › B. Question Cards.docx]

| What defines a “true” rib?   1. The rib attaches directly to the sternum 2. The rib is developed from the embryonic mesoderm 3. The rib is attached to the thoracic vertebrae 4. The rib forms the costal margin   Q1 | How many “false ribs” are there?  Q2 | The neurovascular bundles run along the ______ aspect of the rib.  Q3 | What are the three components that make up the sternum?  Q4 |
| --- | --- | --- | --- |
| At what thoracic level does the inferior vena cava pass through the diaphragm?  Q5 | At what thoracic level does the esophagus pass through the diaphragm?  Q6 | At what thoracic level does the aorta pass posterior to the diaphragm?  Q7 | What do you call the paradoxical movement of the thoracic wall that occurs in the setting of multiple rib fractures?  Q8 |
| The neurovascular bundles travel in costal grooves in a vertical arrangement. What is the typical order of these structures from superior to inferior?   1. Nerve (superior) – Artery (middle) – Vein (inferior) 2. Artery – Nerve – Vein 3. Vein – Artery – Nerve 4. Nerve – Vein – Artery   Q9 | Venous blood from the thoracic wall returns to the heart via the thoracic veins and the _______ ?   1. Azygos system 2. Mesenteric system 3. Portal system 4. Vertebral system   Q10 | Arterial blood supply to the thoracic wall originates from the internal thoracic arteries and ______?   1. Ascending aorta 2. Descending aorta 3. Subclavian artery 4. Vertebral artery   Q11 | To trigger inspiration does intrathoracic pressure increase or decrease?  Q12 |
| Which nerve innervates the diaphragm?  Q13 | What is the term that describes an accumulation of lymph in the pleural cavity?  Q14 | What is the term that describes accumulation of both air and serous fluid in the pleural cavity?  Q15 | How many total lung lobes are there?  Q16 |
| Anatomy & Pharmacology | Anatomy & Pharmacology | Anatomy & Pharmacology | Anatomy & Pharmacology |
| Anatomy & Pharmacology | Anatomy & Pharmacology | Anatomy & Pharmacology | Anatomy & Pharmacology |
| Anatomy & Pharmacology | Anatomy & Pharmacology | Anatomy & Pharmacology | Anatomy & Pharmacology |
| Anatomy & Pharmacology | Anatomy & Pharmacology | Anatomy & Pharmacology | Anatomy & Pharmacology |
| Parasympathetic innervation of the lungs comes primarily from which nerve?  Q17 | Respiratory centers of the brain that initiate breathing are located in which region?   1. Cerebral cortex 2. Amygdala 3. Hypothalamus 4. Medulla Oblongata   Q18 | The reflex to cough in response to chemical or mechanical irritants in the lungs is primarily mediated by which nerve?   1. Hypoglossal nerve 2. Vagus nerve 3. Facial nerve 4. Thoracic nerves   Q19 | Arterial chemoreceptors that respond to low pH and PaO2 are found in “bodies” located near which of the following vessels?   1. Pulmonary artery 2. Subclavian artery 3. Carotid artery 4. Descending aorta   Q20 |
| The main mechanism of movement of pleural fluid out of the pleural space is via   1. Venous recirculation 2. Lymphatic recirculation 3. Osmosis through the parietal pleural membrane 4. Absorption from resident macrophages and phagocytes   Q21 | The respiratory system is derived from the:   1. Embryonic gut 2. Embryonic neural tube 3. Embryonic heart 4. Yolk sac   Q22 | Which of the following is a complication of tracheo-esophageal fistulas in fetal development?   1. Intra-uterine growth restriction 2. Gestational diabetes 3. Pre-eclampsia 4. Polyhydramnios   Q23 | Alpha-1 anti-trypsin deficiency associated emphysema is typically:   1. Upper lobe predominant 2. Lower lobe predominant 3. Centrilobular predominant 4. Right lower lobe predominant   Q24 |
| A patient with cirrhosis has a complication of hydrothorax. This fluid collection is usually located in the:   1. Left pleural space 2. Right pleural space 3. Bilateral pleural spaces 4. Mediastinum   Q25 | Sarcoidosis is diagnosed by (1) typical clinical and radiological manifestations, (2) the presence of noncaseating granulomas, and (3) exclusion of alternative diseases. In the case of pulmonary sarcoidosis, which lymph nodes are classically involved?   1. Sub-pleural 2. Lower lobe 3. Peripheral 4. Tracheal 5. Hilar   Q26 | Rheumatoid arthritis related lung fibrosis often affects which areas of the lung preferentially?   1. Upper lobes 2. Hilar 3. Tracheal 4. Basilar and subpleural 5. Right lower lobe   Q27 | What is the mechanism of action of albuterol?  Q28 |
| What is the mechanism of action of Omalizumab, for treatment of asthma?   1. Monoclonal antibody to IgE 2. Monoclonal antibody to IL-5 3. Monoclonal antibody to IL-4 4. Monoclonal antibody to IL-13   Q29 | This class of medication targets the parasympathetic nervous system to relax airways   1. Beta 2 agonist 2. Anti-cholinergic 3. Inhaled corticosteroid 4. Alpha 2 agonist   Q30 | Roflumilast is a medication used to decrease exacerbations in severe COPD, what is its mechanism of action?   1. Anti-cholinergic 2. Inhaled corticosteroid 3. Phosphodiesterase 4 inhibitor 4. Monoclonal antibody to IL-5   Q31 | What type of adrenergic receptor action leads to bronchorelaxation?   1. Beta 1 agonist 2. Beta 2 agonist 3. Alpha 1 agonist 4. Beta 1 antagonist   Q32 |
| Anatomy & Pharmacology | Anatomy & Pharmacology | Anatomy & Pharmacology | Anatomy & Pharmacology |
| Anatomy & Pharmacology | Anatomy & Pharmacology | Anatomy & Pharmacology | Anatomy & Pharmacology |
| Anatomy & Pharmacology | Anatomy & Pharmacology | Anatomy & Pharmacology | Anatomy & Pharmacology |
| Anatomy & Pharmacology | Anatomy & Pharmacology | Anatomy & Pharmacology | Anatomy & Pharmacology |
| A patient with pulmonary lymphoma is being treated with a regimen including rituximab. Which of the following is true about its mechanism of action?   1. Binds to soluble and transmembranous forms of TNF-alpha 2. Binds to CD20 receptors on cell surfaces 3. Binds to BCR-ABL receptors on cell surfaces 4. Inhibits topoisomerase II activity   Q33 | Which of the following are NOT drugs used for treatment of tuberculosis?   1. Isoniazid 2. Ethambutol 3. Levofloxacin 4. Streptomycin 5. Piperacillin   Q34 | Which of the following is true about treatment of active tuberculosis?   1. Standard of care is treatment with 2 drugs for 9 months 2. Standard of care is treatment with 4 drugs for 2 months, then 2 drugs for 4 months 3. You do not need to avoid drugs that the patient has been exposed to in the past 4. Extensively drug resistant TB is defined as resistance to only a fluoroquinolone   Q35 | In treatment of Tuberculosis, what supplement is given with Isoniazid to prevent peripheral neuropathy side effects?   1. Vitamin B1 2. Vitamin B6 3. Thiamine 4. Folic acid   Q36 |
| Which of the following treatments of Tuberculosis lead to a yellow-orange color to urine, sweat, tears in the patient?   1. Ethambutol 2. Pyrazinamide 3. Rifampin 4. Isoniazid   Q37 | When covering for vancomycin resistant organisms, such as vancomycin resistant enterococcus, which of the following drugs does not have good lung penetration?   1. Linezolid 2. Doxycycline 3. Daptomycin 4. Rifampin   Q38 | A PCP is discussing Bupropion for treatment of nicotine addiction. Which of the following are notable side effects of Bupropion?   1. Lowered seizure threshold 2. Depression 3. Anhedonia 4. Weight gain   Q39 | Which of the following is the most effective studied pharmacotherapy for nicotine addiction?   1. Nicotine patch or gum replacement therapy 2. Buproprion 3. Varenicline 4. Selective serotonin reuptake inhibitors   Q40 |
| What is the mechanism of Varenicline for treatment of nicotine addiction?   1. Dual inhibition of norepinephrine and dopamine reuptake 2. Partial agonist of alpha 4 beta 2 subtype nicotinic cholinergic receptor 3. Selective serotonin reuptake inhibitor 4. Partial agonist of dopamine receptors   Q41 | Which of the following is the preferred pharmacotherapy for long term anticoagulation for a pregnant patient with pulmonary embolism?   1. Warfarin 2. Unfractionated heparin 3. Rivaroxiban 4. Low molecular weight heparin   Q42 | A patient hospitalized with COVID-19 is started on Remdesivir. What is the mechanism of action of remdesivir?   1. Adenosine analog that inhibits RNA-dependent RNA polymerase to inhibit viral replication 2. Spike protein antibody mediated destruction 3. Integrase inhibitor to inhibit viral replication 4. Reverse transcriptase inhibitor to inhibit viral DNA replication   Q43 | A patient is being treated with oseltamivir for influenza. What is the mechanism of action?   1. Neuraminidase inhibitor to prevent release of budding viral progeny from the cell membrane 2. Fusion and entry inhibitor to prevent viral entry into the cell 3. Reverse transcriptase inhibitor to prevent viral replication 4. Integrase inhibitor to prevent viral replication within the nucleus   Q44 |
| What is the recommended time frame from symptom onset for treatment of influenza with antiviral therapy?   1. 24 hours 2. 48 hours 3. 72 hours 4. 96 hours   Q45 | First line treatment for a patient diagnosed with acute allergic bronchopulmonary aspergillosis includes glucocorticoids and ______   1. Amphotericin B 2. Anidulafungin 3. Rifampin and Isoniazid 4. Ceftriaxone and Azithromycin 5. Itraconazole   Q46 | Patients with cystic fibrosis should use several treatments to enhance airway clearance. Which of the following is NOT a typical agent used for this?   1. Inhaled Albuterol 2. Inhaled Hypertonic saline 3. Inhaled DNase 4. Inhaled Mannitol 5. All of the above are typical agents for enhancing airway clearance in CF patients   Q47 | A patient with cystic fibrosis is admitted with concern for pneumonia. Which of the following antibiotics would not be appropriate?   1. Cefepime 2. Ceftriaxone 3. Ciprofloxacin 4. Meropenem   Q48 |
| Anatomy & Pharmacology | Anatomy & Pharmacology | Anatomy & Pharmacology | Anatomy & Pharmacology |
| Anatomy & Pharmacology | Anatomy & Pharmacology | Anatomy & Pharmacology | Anatomy & Pharmacology |
| Anatomy & Pharmacology | Anatomy & Pharmacology | Anatomy & Pharmacology | Anatomy & Pharmacology |
| Anatomy & Pharmacology | Anatomy & Pharmacology | Anatomy & Pharmacology | Anatomy & Pharmacology |
| Which of the following treatments does not have benefit in acute COPD exacerbation?   1. Intravenous magnesium 2. Albuterol-ipratropium 3. Methylprednisolone 4. Azithromycin   Q49 | Which of the following can increase the risk of pneumonia in patients with COPD?   1. Inhaled Tiotropium 2. Inhaled Albuterol 3. Inhaled Fluticasone 4. Inhaled Heli-ox 5. Montelukast   Q50 | Which of the following is NOT an etiology for transudative pleural effusions?   1. Heart failure 2. Decompensated liver failure 3. Malignancy 4. Atelectasis   Q1 | Which of the following mechanisms contributes to development of pleural effusions?   1. Decreased pleural pressure 2. Increased plasma oncotic pressure 3. Decreased microvascular pressure 4. Decreased membrane permeability   Q2 |
| Which of the following is NOT part of Light’s Criteria for exudative pleural effusions?   1. Pleural fluid protein divided by serum protein is greater than 0.5 2. Pleural fluid LDH divided by serum LDH is greater than 0.6 3. Pleural fluid protein is greater than two-thirds the upper limit of normal for serum protein 4. Pleural fluid LDH is greater than two-thirds the upper limit of normal for serum LDH   Q3 | Hypoxemic respiratory failure is defined as:   1. Pulse oximetry below 94% on room air 2. PaO2 below 60 mmHg on room air 3. PaCO2 greater than 50 on room air 4. PH less than 7.3 with PaO2 below 70 mmHg on room air   Q4 | The A-a gradient is defined as:   1. Partial pressure of arterial O2 – partial pressure of alveolar O2 2. Partial pressure of arterial O2 – fraction of inspired oxygen 3. Partial pressure of alveolar O2 – partial pressure of arterial O2 4. Partial pressure of alveolar O2 – arterial concentration of carbon dioxide   Q5 | Which of the following etiologies of hypoxemia would still have a normal A-a gradient?   1. Ventilation – perfusion mismatch 2. Hypoventilation 3. Diffusion abnormality 4. Shunt   Q6 |
| A patient with aero-hypoxia [(ex) in high altitude] has a normal A-a gradient?   1. True 2. False   Q7 | Oxygen therapy does not help in which etiologies of hypoxemia?   1. Aero-hypoxia, such as in high altitude 2. Diffusion abnormality 3. Shunt 4. Ventilation – perfusion mismatch   Q8 | Nicotine binds nicotinic cholinergic receptors, especially alpha 4 beta 2, and this binding leads to:   1. Glutamate release which promotes dopamine release 2. Upregulation of monoamine oxidases 3. GABA release and upregulation 4. Inhibition of NMDA   Q9 | In a patient with massive pulmonary embolism, transthoracic echocardiography can demonstrate McConnell’s sign. Which of these is characteristic of this sign?   1. Tricuspid regurgitation 2. Regional wall motion abnormality in the right ventricle that spares the apex 3. Left ventricular hypokinesis 4. Right atrial dilation and hypokinesis   Q10 |
| A potential EKG finding associated with patients with pulmonary embolism is:   1. S1 Q3 T3 2. Q waves in II, III, aVF 3. Left axis deviation 4. S waves in V1 and R waves in V6 summing to > 35 mm   Q11 | A medical students auscultates for vocal resonance as a patient says “ninety-nine”. Which of the following is true?   1. There should be more resonance with pleural effusion 2. There should be more resonance with consolidation 3. There should be more resonance with bronchospasm 4. There should be more resonance with pneumothorax   Q12 | How do alpha 1 anti-tryptase and other proteolytic enzymes protect the lung in patients who smoke cigarettes?  a. Proteolytic enzymes stimulate alveolar macrophages to phagocytose cigarette toxin deposits  b. Proteolytic enzymes cleave nicotine from its receptors  c. Nicotine increases neutrophil secretion of elastase. Proteolytic enzymes inhibit elastase and prevent lung tissue destruction and development of emphysema  d. Proteolytic enzymes increase surfactant production and prevents surfactant degradation  Q13 | Which of the following is part of management of flash pulmonary edema due to hypertensive emergency?   1. Increase cardiac preload 2. Decrease cardiac afterload 3. Increase cardiac contractility 4. Hyperventilation   Q14 |
| Physiology | Physiology | Anatomy & Pharmacology | Anatomy & Pharmacology |
| Physiology | Physiology | Physiology | Physiology |
| Physiology | Physiology | Physiology | Physiology |
| Physiology | Physiology | Physiology | Physiology |
| How does nephrotic syndrome lead to development of a pleural effusion?   1. Increased RAAS blockade 2. Decreased intravascular oncotic pressure 3. Decreased intravascular hydostatic pressure 4. Increased protein accumulation in the pleural space   Q15 | A 30 year old patient is sedated with fentanyl and midazolam and given local anesthetic and intubated for a procedure. 5 minutes later, his vitals read T 98, HR 108, BP 120/80, SpO2 82% (previously was 98%). Breath sounds are clear bilaterally. Blood gas demonstrates pH 7.38, PaO2 115, PaCO2 44. What is the diagnosis?   1. Intubation of the right mainstem bronchus 2. Pulmonary embolism 3. Opioid overdose 4. Acute respiratory distress syndrome 5. Methemoglobinemia   Q16 | A 25 year old female presents to pulmonology clinic with shortness of breath on exertion. Vitals are normal and lungs are clear at this time. Her symptoms are episodic and mild, occur 1 time per week, do not wake her from sleep. Spirometry data demonstrates:  Prebronchodilator: FEV1/FVC ratio 55%  Postbronchodilator: FEV1/FVC ratio 82%  What is the best therapy to start her on?   1. Fluticasone daily 2. Budesonide-formoterol as needed 3. Ipratropium as needed 4. Anti-IgE 5. Umeclidinium-vilanterol daily   Q17 | A 55 year old female with alcoholic cirrhosis is in the hospital with ascites and encephalopathy. ABG demonstrates pH 7.47 PaO2 105 PaCO2 27 with bicarbonate 20 and anion gap 12. What is the cause of the primary acid base disorder?   1. Lactic acid accumulation 2. Lactulose mediated diarrhea and bicarbonate losses 3. Spontaneous bacterial peritonitis 4. Central stimulation of ventilation due to progesterone 5. Intracranial hemorrhage   Q18 |
| A 30 year old female presents with dyspnea and asthma-like symptoms. She had never had a diagnosis of asthma before and felt like the symptoms started 1 month ago after she painted the interior of her house. Spirometry demonstrates FEV1 85%, FVC 90%, FEV1/FVC 0.85. What is the next best test?   1. High resolution CT of the chest 2. Echocardiogram 3. Methacholine challenge testing 4. Cardiopulmonary rehabilitation 5. Bronchoscopy and bronchoalveolar lavage   Q19 | A 75 year old man presents with dyspnea. On physical exam there is dullness in the right lower lung, crackles on ausculatation, increased tactile fremitus, and egophany. There is 1+ edema bilaterally and he has a history of heart failure and COPD. What does the chest XR show?   1. Lobar Pneumonia 2. Pleural effusion 3. Pneumothorax 4. Pulmonary edema 5. Pulmonary embolism   Q20 | In lung point of care ultrasound, “B lines” are vertical “comet tail” hyperechoic lines extending from the pleura and are an artifact that indicate which of the following:   1. Pneumothorax 2. Pulmonary edema 3. Emphysema 4. Pleural effusion   Q21 | Which form of respiratory support device is most appropriate in management of a COPD exacerbation with hypercarbia?   1. Non-rebreather 2. Continuous positive aiway pressure 3. Bilevel positive airway pressure 4. Nasal cannula   Q22 |
| Which of the following is most likely to improve ventilation / perfusion matching?   1. Placing a patient in a prone position 2. Placing a patient in left lateral recumbant position 3. Hyperventilation 4. Moving to higher altitude   Q23 | What effect does decreasing intrathoracic pressure have on cardiac pre-load?   1. increased preload 2. decreased preload 3. no effect   Q24 | What effect will a bicarbonate infusion have on a patient’s respiratory acid-base relationship?   1. increased pCO2 2. decreased pCO2 3. no change in pCO2   Q25 | A 60 year old woman with COPD presents from nursing home with somnolent mental status. She is found to have pH 7.36, pCO2 62, pO2 69, Bicarbonate 36. What is the acid base relationship?  a. Respiratory acidosis with compensatory metabolic alkalosis  b. Respiratory acidosis without compensatory metabolic alkalosis  c. Metabolic acidosis with compensatory respiratory alkalosis  d. Metabolic acidosis without compensatory respiratory alkalosis  e. Compensated metabolic alkalosis  Q26 |
| A 24 year old man presents to the ED for severe anxiety after being trapped in an elevator for 4 hours. He is found to have pH 7.58, pCO2 20, pO2 90, Bicarbonate 18. What is the acid base relationship?  a. Respiratory alkalosis with compensatory metabolic acidosis  b. Respiratory alkalosis without compensatory metabolic acidosis  c. Metabolic alkalosis with compensatory respiratory acidosis  d. Metabolic alkalosis without compensatory respiratory acidosis  Q27 | A patient visiting Mexico had many Tequila Sunrises, but they were mixed with tapwater ice. Uh oh. The next few days were spent on the toilet. In the ED:  ABG pH 7.30 / pCO2 32 / pO2 90. Na 132, Chloride 110, Bicarb 16. What is the acid base disorder?   1. High anion gap metabolic acidosis 2. Non-gap metabolic acidosis 3. Respiratory acidosis 4. Metabolic alkalosis 5. Mixed gap-non gap metabolic acidosis   Q28 | A patient lost her insulin. She has abdominal pain, vomiting, and increased urinary frequency. ABG pH 7.27 / pCO2 23 / pO2 80. Corrected Na 140, Chloride 100, Bicarb 10, Cr 1.3, BUN 50, Lactate 2. What is the acid base disorder?   1. High anion gap metabolic acidosis 2. Non-gap metabolic acidosis 3. Respiratory acidosis 4. Mixed gap-non gap metabolic acidosis 5. Metabolic acidosis with mixed respiratory acidosis   Q29 | The patient ate some questionable fish. He had significant emesis. ABG pH 7.52 / pCO2 53 / pO2 90. Corrected Na 155, Chloride 100, Bicarb 45, Cr 3, BUN 6. What is the acid base disorder?   1. Respiratory alkalosis 2. Metabolic alkalosis with appropriate respiratory compensation 3. Metabolic alkalosis with mixed respiratory alkalosis 4. Metabolic alkalosis with mixed respiratory acidosis 5. Mixed metabolic alkalosis and metabolic acidosis   Q30 |
| Physiology | Physiology | Physiology | Physiology |
| Physiology | Physiology | Physiology | Physiology |
| Physiology | Physiology | Physiology | Physiology |
| Physiology | Physiology | Physiology | Physiology |
| A patient with history of DM2, CAD, HF, chronic COPD presents with acute viral GI illness with nausea/vomiting/diarrhea.  pH 7.25 / pCO2 60 / pO2 100. Na 140, Chloride 118, Bicarb 12. What is the acid base disorder?   1. Respiratory acidosis 2. High anion gap metabolic acidosis with respiratory compensation 3. Mixed Respiratory acidosis and metabolic acidosis 4. Respiratory acidosis with appropriate metabolic compensation 5. Mixed high anion gap and non gap metabolic acidosis   Q31 | A patient with alcoholic cirrhosis goes into withdrawal and starts retching. He presents to the ED with hematemesis and melena. He is found to be hypotensive, tachycardic, and tachypneic.  pH 7.1 / pCO2 18 / pO2 80. Na 130, Chloride 90 , Bicarb 10. What is the acid base disorder?   1. Mixed high anion gap and non-gap metabolic acidosis 2. Pure high anion gap metabolic acidosis 3. Primary Respiratory alkalosis with compensatory metabolic acidosis 4. Primary high anion gap metabolic acidosis with mixed respiratory alkalosis   Q32 | A patient with an acute COPD exacerbation is found to have pneumonia. He is treated with nebulizers, steroids, and antibiotics. An ABG is drawn:  pH 7.33 / pCO2 70 / pO2 60 with Bicarb 27. What is the acid base disorder?   1. Respiratory acidosis with metabolic acidosis 2. Respiratory acidosis with compensatory metabolic alkalosis 3. Metabolic acidosis with respiratory acidosis 4. Metabolic acidosis with respiratory alkalosis   Q33 | A patient was administered 3 boluses of fentanyl and 15 minutes later he was found to have respiratory rate of 5 per minute. A blood gas is drawn and the nurse starts ventilating for him with the bag valve mask.  pH 7.1 / pCO2 70 / pO2 45 with Bicarb 24. What is the acid base disorder?   1. Respiratory acidosis with metabolic compensation 2. Respiratory acidosis without metabolic compensation 3. Metabolic acidosis with mixed respiratory acidosis 4. Metabolic alkalosis with mixed respiratory acidosis   Q34 |
| A patient has a cardiac arrest at the dialysis unit. After appropriate cardiopulmonary resuscitation, return of spontaneous circulation is achieved. A blood gas is drawn at this time.  pH 7.1 / pCO2 60 / pO2 120. Na 140, Chloride 100, Bicarb 8. What is the acid base disorder?  a. Pure high anion gap metabolic acidosis  b. Respiratory acidosis with compensatory metabolic alkalosis  c. Mixed gap – non gap metabolic acidosis  d. Pure high anion gap metabolic acidosis with compensatory respiratory alkalosis  e. Mixed high anion gap metabolic acidosis with respiratory acidosis  Q35 | The patient ingests a mystery substance at a party. She has altered mental status and diarrhea. In the ED, a blood gas is drawn.  pH 7.3 / pCO2 15 / pO2 100. Na 140, Chloride 120, Bicarb 10. What is the acid base disorder?  a. Pure high anion gap metabolic acidosis  b. Mixed Non gap metabolic acidosis with respiratory alkalosis  c. Mixed Non gap metabolic acidosis with appropriate respiratory compensation  d. Mixed gap non gap metabolic acidosis with appropriate respiratory compensation  e. Mixed high anion gap metabolic acidosis with respiratory alkalosis  Q36 | A patient is intubated for pancreatitis ARDS. She has multiorgan failure and shock and has received many amps of Bicarb. A blood gas is drawn.  pH 6.9 / pCO2 80 / pO2 70. Na 140, Chloride 110, Bicarb 8. What is the acid base disorder?  a. High gap metabolic acidosis with mixed respiratory acidosis  b. Non gap metabolic acidosis with mixed respiratory acidosis  c. Mixed High gap and non-gap metabolic acidosis with respiratory acidosis  d. High gap metabolic acidosis with appropriate respiratory compensation  Q37 | A patient overdoses on aspirin and presents to the ED.  ABG pH 7.48 / pCO2 14 / pO2 90. Na 126, K 2.2, Chloride 98, Bicarb 8. What is the acid base disorder?  a. Respiratory alkalosis with compensatory metabolic acidosis  b. Primary respiratory alkalosis with mixed gap and non gap metabolic acidosis  c. Metabolic alkalosis with compensatory respiratory acidosis  d. Metabolic acidosis with compensatory respiratory alkalosis  Q38 |
| A patient was using marijuana for pain control developed hyperemesis syndrome. The patient is in the ED with uncontrolled pain, agitation, and panic. ABG pH 7.57 / pCO2 20 / pO2 80. Na 128, Chloride 78, Bicarb 23, Cr 1.8, BUN 60, Lactate 4. What is the acid base disorder?  a. Primary Respiratory alkalosis with compensatory metabolic acidosis  b. Mixed Respiratory alkalosis with metabolic acidosis  c. Mixed Respiratory alkalosis with mixed metabolic alkalosis  d. Mixed Respiratory alkalosis with mixed high anion gap metabolic acidosis and metabolic alkalosis  e. Mixed metabolic alkalosis with compensatory respiratory acidosis  Q39 | Which of the following lung function parameters can NOT be measured by spirometry?   1. Vital capacity (VC) 2. Forced Vital Capacity (FVC) 3. Forced expired volume in one second (FEV1) 4. Total lung capacity (TLC)   Q40 | A 70 year old female smoker presents to pulmonology clinic. What do the PFTs suggest?  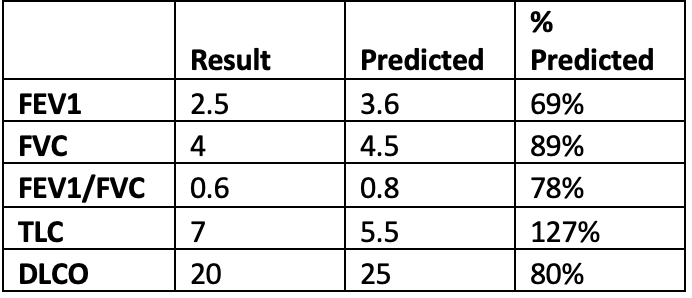   1. Acute bronchitis 2. Emphysema 3. Pneumonia 4. Neuromuscular disease 5. Upper airway obstruction   Q41 | A 55 year old female with rheumatoid arthritis and tobacco use disorder presents to pulmonology clinic. What do the PFTs suggest?  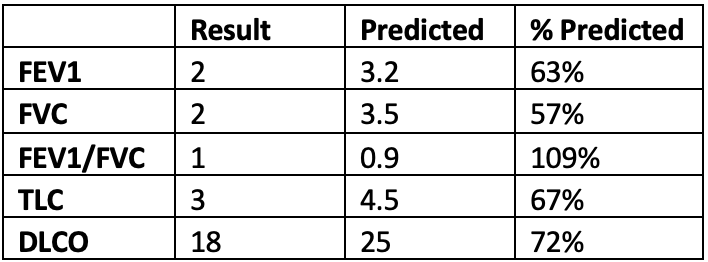   1. Interstitial lung disease 2. Emphysema 3. Asthma 4. Bronchiectasis   Q42 |
| Which of the following would not produce a restrictive spirometry pattern on PFTs?   1. Bronchitis 2. Hepatic hydrothorax 3. Neuromuscular diseases 4. Morbid obesity   Q43 | What does this flow volume loop suggest? (Restrictive disease, Obstructive disease, Fixed upper airway obstruction)  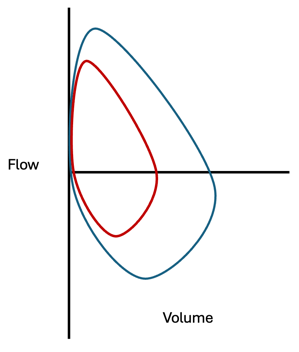  Q44 | What does this flow volume loop suggest? (Obstructive disease, restrictive disease, fixed upper airway obstruction)  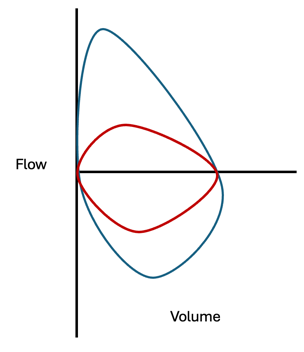  Q45 | What does this flow volume loop suggest? (Obstructive disease, restrictive disease, fixed upper airway obstruction)  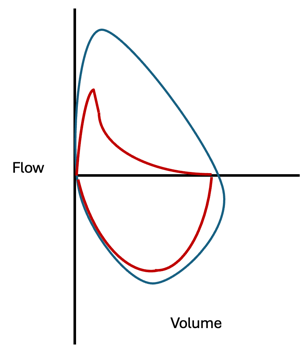  Q46 |
| Physiology | Physiology | Physiology | Physiology |
| Physiology | Physiology | Physiology | Physiology |
| Physiology | Physiology | Physiology | Physiology |
| Physiology | Physiology | Physiology | Physiology |
| In pulmonary function testing, a certain medication is given as a bronchoprovocation challenge to aid in the diagnosis of asthma. What is this medication?   1. Albuterol 2. Fluticasone 3. Prednisone 4. Tiotropium 5. Methacholine   Q47 | A patient develops cardiac arrest in the hospital. CPR is initiated and she is intubated. Capnography attached to the endotracheal tube demonstrates end tidal partial pressure of CO2 is 12 mmHg (EtCO2). What would you expect the capnography to show in the setting of poor quality compressions?   1. Gradual increase in EtCO2 2. Gradual decrease in EtCO2 3. Sudden increase in EtCO2 4. EtCO2 of > 30   Q48 | Which of the following is associated with a pleural effusion with a triglyceride level >110 mg/dL?   1. Chylothorax 2. Pulmonary embolism 3. Nephrotic syndrome 4. Connective tissue disease   Q49 | A patient develops cardiac arrest in the hospital. CPR is initiated and she is intubated. Capnography attached to the endotracheal tube demonstrates end tidal partial pressure of CO2 is 12 mmHg (EtCO2). After 10 minutes of resuscitation, the EtCO2 rapidly increases to 37 mmHg. What explains this capnography finding?   1. Poor quality compressions 2. Pneumothorax due to compressions 3. Right mainstem intubation 4. Return of spontaneous circulation 5. Esophageal intubation   Q50 |
| Which of the following mechanisms are least effective in the response to tuberculosis infection?   1. IL-12 2. Interferon—gamma 3. Tumor necrosis factor-alpha 4. CD20   Q1 | Which of the following are NOT a reason for pulmonary tuberculosis’s preference for the upper lung lobes?   1. Lymphatic clearance is decreased in the upper lobes 2. Regional high oxygen tension in the upper lobes 3. Relative over-ventilation in the upper lobes 4. Decreased T cell response in the upper lobes   Q2 | Which of the following facts are NOT true about Bacillus of Calmette and Guerin (BCG) vaccination for tuberculosis?  a. The vaccination is a live attenuated strain of Mycobacterium bovis  b. It is not used in the USA because of low TB risk and interference with PPD screening  c. Overall, it is approximately 50% effective  d. People who received BCG 10 years ago and are tuberculin skin test positive likely are immune and not infected.  Q3 | In a patient with hypercarbic respiratory failure due to a COPD exacerbation, which of the following changes will help?   1. Increase tidal volume 2. Decrease respiratory rate 3. Increase FiO2 4. Increase positive end-expiratory pressure   Q4 |
| What type of immune hypersensitivity reaction does the purified protein derivative tuberculin skin test elicit?   1. Type I 2. Type II 3. Type III 4. Type IV   Q5 | Which of the following is NOT a feature of a pleural effusion from rheumatoid arthritis?   1. Exudative 2. High glucose 3. Rheumatoid Factor positive 4. High protein   Q6 | Which of the following is NOT a feature of malignant pleural effusion?   1. Exudative 2. Low glucose 3. High pH 4. High LDH   Q7 | In a patient with hypercarbic respiratory failure due to a COPD exacerbation treated with BIPAP, which of the following is most appropriate?  a. Increase the inspiratory pressure, keep the expiratory pressure the same  b. Increase the inspiratory pressure and the expiratory pressure  c. Increase the expiratory pressure and keep the inspiratory pressure the same. d. Change to continuous positive airway pressure.  Q8 |
| Which of the following is the studied indication for CODEX dosing (dexamethasone 20mg x 5 days followed by 10mg x 5 days) for treatment of COVID-19?   1. Hypoxia requiring supplemental oxygen 2. Moderate to severe ARDS, as defined by Berlin criteria 3. ICU admission 4. Requiring antibiotics   Q9 | Which of the following is not typically a part of the laboratory workup for a new diagnosis of community acquired pneumonia being admitted to the hospital?   1. SARS-CoV-2 rapid PCR test 2. Legionella urine antigen test 3. Blood or sputum cultures 4. CMV antigen or PCR test   Q10 | A patient presents with community acquired pneumonia and no prior hospital exposures, all of the following are common organisms except:   1. Streptococcus pneumoniae 2. Haemophilus influenzae 3. Pseudomonas aeruginosa 4. Staphylococcus aureus   Q11 | All of the following disease processes cause diffusion abnormalities on pulmonary function testing except for:   1. Pneumonia 2. Congestive heart failure 3. Cystic fibrosis 4. Interstitial lung disease 5. Asthma   Q12 |
| Physiology | Physiology | Physiology | Physiology |
| Pathophysiology | Pathophysiology | Pathophysiology | Pathophysiology |
| Pathophysiology | Pathophysiology | Pathophysiology | Pathophysiology |
| Pathophysiology | Pathophysiology | Pathophysiology | Pathophysiology |
| Restrictive lung diseases have all the below characteristics on pulmonary function testing, except:   1. Normal residual volume 2. Low lung volumes 3. FEV1 to FVC ratio < 70% 4. No bronchodilator response   Q13 | What is the most common cause of Acute Respiratory Distress Syndrome (ARDS)?   1. Sepsis 2. Congestive heart failure 3. Blood product transfusion reaction 4. Drug toxicity 5. Injury from inhaled toxins or chemicals   Q14 | A patient is found to have acute respiratory distress syndrome (ARDS) and is intubated and mechanically ventilated. What should the target tidal volumes be? (in ml/kg of ideal body weight)   1. 6-8 2. 8-10 3. 10-12 4. 12-14   Q15 | The Berlin criteria for Acute Respiratory Distress Syndrome include all of the following except:   1. Acute onset, less than 1 week 2. Bilateral opacity on chest XR or CT scan 3. Ratio of the arterial partial oxygen tension, or PaO2, to the fraction of inspired oxygen, or FiO2 for severe ARDS is < 100 4. SpO2 less than 88% on room air   Q16 |
| All of the following are subtypes of Non-Small Cell Lung Cancer except:   1. Squamous 2. Endothelial 3. Adenocarcinoma 4. Large cell neuroendocrine carcinoma   Q17 | Which of the following symptoms is not typical for an Apical Pancoast lung cancer causing Horner’s Syndrome?   1. Lateral gaze palsy 2. Drooping upper eyelid 3. Constricted pupil 4. Anhidrosis   Q18 | The following are paraneoplastic syndromes commonly associated with Small cell carcinoma except:   1. Syndrome of inappropriate ADH 2. Eaton Lambert syndrome 3. Cushings syndrome 4. Nephrotic syndrome   Q19 | Which of the following are molecular targets for lung cancer treatment?   1. BCR-ABL 2. TNF-alpha 3. PD-L1 4. CD20   Q20 |
| All of the following are World Health Organization defined groups for pulmonary hypertension except:   1. Pulmonary arterial hypertension 2. Pulmonary hypertension due to left heart disease 3. Pulmonary hypertension due to liver disease 4. Pulmonary hypertension due to lung disease 5. Pulmonary hypertension due to chronic thromboembolic disease   Q21 | A patient without prior history of sarcoidosis presents with a high suspicion for pulmonary sarcoidosis that requires definitive diagnostic testing. Which of the following is the best approach?   1. Biopsy an enlarged cutaneous lesion thought to be also due to sarcoid 2. Transbronchial biopsy of a hilar lymph node 3. Transcutaneous biopsy of parenchymal lung tissue 4. Measurement of serum angiotensin converting enzyme   Q22 | Allergic bronchopulmonary aspergillosis is characterized by all of the following except:   1. Eosinophilic pneumonia 2. Mucoid impaction of the bronchi 3. Bronchocentric granulomatosis 4. Restrictive lung disease on pulmonary function testing   Q23 | Allergic bronchopulmonary aspergillosis usually occurs in patients with:   1. Asthma 2. COPD 3. Sarcoidosis 4. Tuberculosis   Q24 |
| Patients with cystic fibrosis are commonly colonized with bacteria in their respiratory system. Which of the following is NOT a common organism in these patients?   1. Staphylococcus aureus 2. Pseudomonas aeruginosa 3. Stenotrophomonas maltophila 4. Burkholderia cepacia complex 5. Tuberculosis mycobacterium   Q25 | All of the following are characteristics of cystic fibrosis pulmonary disease, except:   1. Neutrophilic release of elastase that overwhelms anti-proteases which increases tissue destruction 2. Bronchiectasis 3. Respiratory alkalosis 4. Impaired mucociliary clearance 5. Airway hyperreactivity responsive to bronchodilator therapy   Q26 | All of the following are associated conditions with cystic fibrosis, except:   1. Obstructive sleep apnea 2. Tracheomalacia 3. Allergic bronchopulmonary aspergillosis 4. Pulmonary hypertension 5. Small cell lung cancer   Q27 | A 20 year old patient with cystic fibrosis presents to your outpatient clinic for vaccinations. Which of the following vaccinations are appropriate for this patient?   1. Seasonal influenza 2. Pneumococcal vaccine 3. COVID-19 vaccine 4. HPV vaccine 5. All of the above   Q28 |
| Pathophysiology | Pathophysiology | Pathophysiology | Pathophysiology |
| Pathophysiology | Pathophysiology | Pathophysiology | Pathophysiology |
| Pathophysiology | Pathophysiology | Pathophysiology | Pathophysiology |
| Pathophysiology | Pathophysiology | Pathophysiology | Pathophysiology |
| The Global Initiative for Chronic Obstructive Lung Disease (GOLD) guidelines classify COPD based on all of the following except:   1. Exacerbation history / hospitalizations 2. Modified Medical Research Council dyspnea questionaire 3. COPD assessment test 4. DLCO   Q29 | Which of the following are signs of hypercapnia?   1. Asterixis 2. Confusion 3. Somnolence 4. Increased respiratory rate 5. All of the above   Q30 | A patient has a ground level fall and a resulting radial fracture. An arterial blood gas checked after she presented to the ED was pH 7.50, pO2 140, pCO2 26, HCO3 21. Which of the following etiologies is consistent with this blood gas in this clinical setting?   1. Tachypnea from pain 2. Submassive Pulmonary embolism 3. Asthma exacerbation 4. Pulmonary edema   Q31 | The most common mutation for Cystic Fibrosis is the CF transmembrane conductance regulator (CFTR) gene mutation delta-F508 on chromosome:   1. 3 2. 5 3. 7 4. 9   Q32 |
| An abnormal enlargement and decreased function of the right ventricle as a consequence of increased pulmonary hypertension is termed:   1. Pulmonic regurgitation 2. Cor Pulmonale 3. Ventricular septal defect 4. Takutsubo cardiomyopathy   Q33 | WHO Group 2 pulmonary hypertension is due to this etiology:   1. Lung disease 2. Left heart disease 3. Chronic thromboembolic lung disease 4. Pulmonary arterial hypertension   Q34 | WHO Group 4 pulmonary hypertension is due to this etiology:   1. Lung disease 2. Left heart disease 3. Chronic thromboembolic lung disease 4. Pulmonary arterial hypertension   Q35 | WHO Group 3 pulmonary hypertension is due to this etiology:   1. Lung disease 2. Left heart disease 3. Chronic thromboembolic lung disease 4. Pulmonary arterial hypertension   Q36 |
| A 80 year old male with COPD and alcohol use disorder complains of 6 weeks of progressively worsening dyspnea and productive cough. Chest XR demonstrates a right lower lobe cavitary lesion with an air fluid level in the right lower lobe. Which of the following is the best treatment to initiate?   1. Rifampin, Isoniazid, Pyrazinamide, Ethambutol 2. IV ampicillin-sulbactam 3. IV metronidazole 4. IV Ceftaroline 5. IV Vancomycin   Q37 | Which of the following arterial blood gases is most consistent with a patient with chronic COPD, presenting with baseline dyspnea on exertion and vitals T 37.1, BP 140/95, HR 88, respirations 20/min.  a. PH 7.3, bicarbonate 33, anion gap 9  b. PH 7.3, bicarbonate 24, anion gap 12  c. PH 7.3, bicarbonate 18, anion gap 16  d. PH 7.46, bicarbonate 24, anion gap 12  e. PH 7.46, bicarbonate 33, anion gap 9  Q38 | A 32 year old male presents with dyspnea and decreased functional status that is progressively worsening over months. He does not smoke cigarettes, he works a desk job, has no previous lung disease. Chest XR imaging demonstrates basilar emphysematous changes. Spirometry shows FEV1 58%, FVC 70%, FEV1/FVC 62% and postbronchodilator FEV1 58%. Labs are unremarkable except for mild transaminitis. What is the best next test?   1. Alpha-1 antitrypsin testing 2. Repeat pulmonary function testing in 3 months 3. Transthoracic echocardiogram 4. Empiric initiation of asthma treatment 5. High resolution CT of the chest   Q39 | All of the following are findings consistent with obesity hypoventilation syndrome and sleep apnea except:   1. Right heart dilation 2. Bicarbonate elevation 3. Hypercarbia 4. Anemia 5. FEV1/FVC ratio of 88%   Q40 |
| Interstitial lung disease associated with polymyositis or dermatomyositis is heavily correlated with the presence of which of the following antibodies?   1. Anti-Jo 1 2. Anti-Mitochondrial 3. Anti-double stranded DNA 4. Anti-IgE 5. Anti-SSA   Q41 | Which of the following autoimmune disorders is the most common cause of pulmonary-renal syndrome?   1. Eosinophilic granulomatosis with polyangiitis (Churg Strauss) 2. Microscopic polyangiitis 3. Rheumatoid arthritis 4. SLE 5. Takayasu arteritis   Q42 | A patient is being treated with high dose prednisone for Giant cell arteritis and presents with cough. Chest CT demonstrates ground glass opacities and cavitary nodules. Galactomannan positive in the serum. What is the etiology?   1. Aspergillosis 2. Histoplasmosis 3. Mycobacterial tuberculosis 4. Mycobacterium avium complex 5. Small cell lung cancer   Q43 | Honeycombing is a radiographic finding of clustered cystic air spaces (usually 3-10 mm in diameter) that are usually basilar, peripheral, and subpleural. This finding is most commonly associated with:   1. Small cell lung cancer 2. Alpha-1 antitrypsin deficiency 3. Usual interstitial pneumonia (UIP) 4. Asthma 5. Pulmonary embolism   Q44 |
| Pathophysiology | Pathophysiology | Pathophysiology | Pathophysiology |
| Pathophysiology | Pathophysiology | Pathophysiology | Pathophysiology |
| Pathophysiology | Pathophysiology | Pathophysiology | Pathophysiology |
| Pathophysiology | Pathophysiology | Pathophysiology | Pathophysiology |
| Eosinophilic granulomatosis with polyangiitis is an ANCA vasculitis characterized by multiorgan involvement. Asthma is a cardinal feature, which of the following is the next most common clinical feature?   1. Gastritis 2. Renal artery stenosis 3. Rhinosinusitis 4. Monoarticular arthritis 5. Aortic root dilation   Q45 | In a patient with heparin induced thrombocytopenia and new diagnosis of pulmonary embolism, which of the following is the best management?   1. Warfarin 2. Low molecular weight heparin 3. Argatroban 4. Inferior vena cava filter placement   Q46 | A 30 year old female has a pulmonary embolus diagnosed in the setting of a COVID infection. Which of the following is the most appropriate therapy?   1. Apixaban for 1 month 2. Rivaroxaban for 3 months 3. Apixaban for 1 year 4. Heparin for 6 months 5. Lovenox for 1 year   Q47 | A 20 year old patient is diagnosed with narcolepsy due to severe daytime somnolence, sleep paralysis, disrupted nocturnal sleep. What is the best next step in management?   1. Modafinil 2. CPAP 3. Improve sleep hygiene 4. Methylphenidate   Q48 |
| Loffler syndrome is characterized by all of the following except:   1. Helminth infection with transpulmonary migration 2. Peripheral blood eosinophilia 3. Hemoptysis 4. Transient pulmonary radiographic opacities   Q49 | The following are contra-indications to thrombolytic therapy for patients with pulmonary embolism, except:   1. Suspected aortic dissection 2. Malignant intracranial neoplasm 3. Ischemic stroke within the last three months 4. Hemodynamic instability   Q50 | Which of the following treatments for Tuberculosis cause a reversible and dose dependent decrease in your green-red visual discrimination?   1. Ethambutol 2. Pyrazinamide 3. Rifampin 4. Isoniazid   Q1 | Which of the following is the preferred treatment for Mycobacterium Avium Complex in HIV patients?  a. Rifampin and Isoniazid for 9 months  b. Isoniazid, Rifampin, Pyrazinamide, and Ethambutol for 2 months then Isoniazid and Rifampin for 4 months  c. Clarithromycin and ethambutol for 12 months  d. Ampicillin-Sulbactam and Clarithromycin for 6 weeks with followup CT chest imaging  Q2 |
| In which stage of airway and respiratory development do true alveoli form?   1. Embryonic 2. Pseudoglandular (approximate gestational week 5 – 18) 3. Canalicular (approximate gestational week 16 – 27) 4. Saccular (approximate gestational week 24 – birth)   Q3 | In which stage of airway and respiratory development does surfactant first start being produced?   1. Embryonic 2. Pseudoglandular (approximate gestational week 5 – 18) 3. Canalicular (approximate gestational week 16 – 27) 4. Saccular (approximate gestational week 24 – birth)   Q4 | The CURB-65 score is used to estimate mortality of community acquired pneumonia to help guide decision-making for inpatient admission vs outpatient management. Which of the following is not a component of this score?   1. Age 2. Altered mental status 3. BUN 4. Blood pressure 5. Respiratory acidosis   Q5 | A patient in the post-anesthesia care unit is found to be cyanotic and apneic, with a pulse and normotension. After starting bag valve mask ventilation, supplemental oxygen, and preparing for intubation, which of the following actions should be taken?   1. Serum and urine drug screen for opiates 2. Give Narcan empirically 3. Start a pressor such as norepinephrine 4. Give antibiotics empirically 5. Order a bedside echocardiogram   Q6 |
| A patient with cirrhosis (not a transplant candidate) and persistent hepatic hydrothorax presents with shortness of breath. Which of the following is the most appropriate management of the hydrothorax?   1. Chest tube placement 2. Serial thoracentesis based on symptoms 3. Pleurodesis 4. Long term prophylactic antibiotics   Q7 | In Guillain-Barre syndrome, which of the following methods is the best monitoring to determine if a patient requires ventilatory support?   1. PaO2 2. SpO2 3. Forced Vital Capacity 4. Forced expiratory volume in 1 second 5. Total lung capacity   Q8 | Ashbaugh and Petty first named the syndrome of respiratory failure, “Acute Respiratory Distress Syndrome”, in 1967 in *Lancet*. In what setting was ARDS most notably described and studied in the 1960s and 70s?   1. Lung transplant surgery 2. Study of congestive heart failure 3. Wartime lung injury from surgery and blood transfusions 4. Inhalation injury from fires, studied in firefighters and victims   Q9 | All of the following ventilator strategies are utilized for management of Acute Respiratory Distress Syndrome except:   1. Permissive hypercapnea 2. High tidal volume goal of 10-12 cc/kg ideal body weight 3. Minimize volume overload with diuresis 4. Use of sedatives and paralytics to reduce work of breathing and improve compliance with the ventilator   Q10 |
| Pathophysiology | Pathophysiology | Pathophysiology | Pathophysiology |
| Modifier | Modifier | Pathophysiology | Pathophysiology |
| Modifier | Modifier | Modifier | Modifier |
| Modifier | Modifier | Modifier | Modifier |
|  |  |  |  |
|  |  |  |  |
|  |  |  |  |
|  |  |  |  |
| Modifier | Modifier | Modifier | Modifier |
| Modifier | Modifier | Modifier | Modifier |
| Modifier | Modifier | Modifier | Modifier |
| Modifier | Modifier | Modifier | Modifier |
| Which of the following is the best explanation for why slowing down the respiratory rate of a patient with severe asthma exacerbation can improve their respiratory status?   1. Prolong expiratory time and improve hyperinflation 2. Increases the patient’s total lung capacity 3. Improves gas exchange 4. Decreases the patient’s FEV1   Q11 | The US preventative services task force (USPSTF) would recommend a low dose CT scan for lung cancer screening in your 60 year old patient with:   1. 15 pack-year smoking history and current smoker 2. 30 pack-year smoking history who quit smoking 20 years ago 3. 20 pack-year smoking history, current smoker, advanced pancreatic cancer with 6 month life expectancy 4. 20 pack-year smoking history who quit smoking 5 years ago   Q12 | The Pulmonary Embolism Severity Index (PESI) is a scoring system to guide prognosis after pulmonary embolism. All of the following are components of this system except:   1. History of cancer 2. Heart failure 3. Blood pressure 4. Current use of anticoagulation   Q13 | Which of the following is NOT a criteria for acute respiratory distress syndrome (ARDS)?   1. Onset of respiratory symptoms within 1 week of insult 2. Bilateral lung opacities 3. Signs of cardiac failure and fluid overload 4. PaO2/FiO2 ratio < 300   Q14 |
| A patient with newly diagnosed COVID-19 has a 2L oxygen requirement. Their D-dimer is normal. They have no history of acute ischemic stroke, myocardial infarction, deep vein thrombosis or pulmonary embolism. Their labs are otherwise unremarkable. What is the best approach to anticoagulation?  a. Prophylactic anticoagulation with low molecular weight heparin  b. Therapeutic anticoagulation with unfractionated heparin  c. Therapeutic anticoagulation with low molecular weight heparin  d. Therapeutic anticoagulation with apixaban or rivaroxaban  Q15 | A patient with allergic bronchopulmonary aspergillosis is unable to taper off of prednisone despite treatment with antifungal drugs and management of asthma. What is the next best approach?   1. Start anti- IL 5 agent such as mepolizumab 2. Start anti- TNF alpha agent such as infliximab 3. Change to dexamethasone 4. Start empiric antibiotics   Q16 | A patient has persistent cough after a viral respiratory illness. She complains of a nonpurulent cough for 2 weeks that initially had productive yellow sputum and now has thin white mucous. There is no fever, chills, vital sign abnormalities and the lungs are clear. What is the next best step in management?   1. Chest CT 2. Moxifloxacin 3. Sputum cultures 4. Albuterol nebulizer   Q17 | A patient is diagnosed with invasive aspergillosis characterized by several cavitary lung nodules and hemoptysis in the setting of immunosuppression. In addition to reducing immunosuppression, what is the next best therapy?   1. Rifampin 2. Anidulafungin 3. Voriconazole 4. Vancomycin   Q18 |
| Patients with cystic fibrosis should undergo genotyping to guide treatment for:   1. Antibiotic therapy resistances 2. CF transmembrane conductance regulator modulators 3. Anti-IL 5 therapy 4. Anti-Ig E therapy   Q19 | A patient presents to your office for followup of COPD. Her resting PaO2 is 55mmHg and oxygen saturation is 88%. Her FEV1/FVC is 55%. Which of the following treatments can prolong survival?   1. Long-term oxygen therapy 2. Inhaled long acting beta agonist combined with corticosteroid 3. Inhaled long acting muscarinic antagonist 4. Inhaled short acting beta agonist 5. Nightly CPAP   Q20 | Most COPD patients target pulse ox of at least 88%. Which of the following comorbid conditions will raise your targeted pulse ox goal to 90%?   1. Aortic stenosis 2. Sarcoidosis 3. Cor pulmonale 4. Asthma 5. Chronic kidney disease   Q21 | A patient with COPD presents with an exacerbation. He is started on appropriate medical therapy and nebulizers. 3 hours later, his Arterial blood gas demonstrates pH 7.22, PaCO2 82, PaO2 72. He is lethargic but awake and oriented. Which of the following is the best next step in management?   1. Increase oxygen supplementation 2. Intubation and mechanical ventilation 3. Continuous albuterol 4. Bilevel positive airway pressure ventilation   Q22 |
| A patient with history of hypertension presents with progressive dyspnea and is found to have diastolic heart failure. Echocardiogram shows a left ventricular ejection fraction of 55%. Which of the following medications is indicated for this condition and reduced the combined risk of cardiovascular death and hospitalization?   1. Metoprolol 2. Valsartan-sacubitril 3. Empagliflozin 4. Apixaban   Q23 | A 60 year old man with 40 pack year smoking history presents to clinic for an incidentally discovered 2.4cm spiculated nodule in the periphery of the right lung. He has no B symptoms and vitals are normal. No family history of cancer. What is the best management of the nodule?   1. Sputum culture and cytology 2. Surgical wedge resection 3. Bronchoscopy with transbronchial biopsy 4. Repeat CT scan in 6 months 5. Smoking cessation   Q24 | A 75 year old patient presents to the ED with shortness of breath. He was discharged to a nursing home 3 days ago after being treated for pneumonia and completed his antibiotics. Presenting vitals T100.4 HR 110 BP 110/80 SpO2 89% on room air. Chest XR shows a resolving density in the right lung base. Lungs are clear to your auscultation. What is the best next step?   1. V/Q scan 2. CT pulmonary angiography 3. Antibiotics 4. High resolution CT chest   Q25 | In eosinophilic granulomatosis with polyangiitis (EGPA), the prodromal phase is characterized by asthma and atopic diseases, the eosinophilic phase is notable for extravascular eosinophila to organs such as the lungs and GI tract. The vasculitis phase is characterized by all of the following except:   1. Myocarditis 2. ANCA associated glomerulonephritis 3. Palpable purpura 4. Aortic dissection   Q26 |
| Modifier | Modifier | Modifier | Modifier |
| Modifier | Modifier | Modifier | Modifier |
| Modifier | Modifier | Modifier | Modifier |
| Modifier | Modifier | Modifier | Modifier |
| A 32 year old African American male presents with months of progressive fatigue, nonproductive cough, fevers, night sweats. Labs are unremarkable. He has erythema nodosum on physical exam and lungs are clear bilaterally. Chest imaging demonstrates bilateral hilar lymphadenopathy and no parenchymal consolidations. What is the best next step?   1. Biopsy of the erythema nodosum skin lesion 2. Endobronchial biopsy of a hilar lymph node 3. Surgical resection of a complete lymph node 4. High resolution CT imaging of the chest   Q27 | A 50 year old male in the surgical ICU is intubated after an extensive bowel surgery and is post operative day 1. She is on IV antibiotics, fentanyl, midazolam and you are called to bedside because she is hypertensive to 190/120, agitated, and there is ventilator dyssynchrony. Other vitals are T37 HR 110 RR 35 and SpO2 98%. Arterial blood gas: pH 7.50, PaO2 110, PaCO2 25. The labs are all unchanged from prior. Chest imaging is unremarkable. What is the best next step?   1. Start a titratable anti-hypertensive agent 2. Decrease the tidal volume 3. Increase fentanyl dose 4. Start a paralytic to improve vent synchrony   Q28 | A patient with COPD on long term oxygen therapy of 1L by nasal cannula plans on flying from Virginia to New York to visit family. She needs 1L of oxygen at rest and maintains SpO2 of 96%. What should be done for this patient for her flight?   1. Pulmonary function testing 2. Pharmacologic stress test 3. Increase supplemental oxygen by 2L/min for the flight 4. Six minute walk test 5. No changes are needed   Q29 | A 55 year old man is admitted with pneumonia. Chest imaging was notable for a moderate to large right sided pleural effusion. A diagnostic thoracentesis was performed and notable for pH 7.15, glucose 36, and gram stain with many neutrophils and bacteria. What is the next best step?   1. Repeat thoracentesis tomorrow 2. Repeat chest XR tomorrow to guide whether to perform thoracentesis 3. Chest tube placement immediately 4. Thoracic surgery consult for video assisted thorascopic surgery and pleurodesis   Q30 |
| An 80 year old male is intubated and mechanically ventilated after his pneumonia and sepsis progressed to ARDS. The ventilator is on assist control mode with RR 16, Tidal volume 480, Positive end expiratory pressure (PEEP) 5mmHg and FiO2 75%. ABG shows pH 7.32, pCO2 48, pO2 45. What is the next best step?   1. Increase PEEP 2. Increase Tidal volume 3. Increase Respiratory rate 4. Change to Pressure control ventilatory mode   Q31 | A 55 year old female with history of COPD presents to clinic with worsening dyspnea over the past months. She is only using an albuterol inhaler several times a week. Spirometry shows FEV1 55%, FVC 90% and FEV1/FVC 65% with no bronchodilator improvement. Chest imaging does not show any infiltrates, vitals are normal, lung exam with mild expiratory wheezing. He has no hospitalizations or major exacerbations. What is the best therapy to start (in addition to his albuterol inhaler)?   1. ICS 2. LABA-ICS 3. Long acting muscarinic antagonist 4. Long term oxygen therapy   Q32 | A 65 year old man presents with dyspnea after a coronary artery bypass procedure 3 months ago. He has worsened dyspnea when supine. SpO2 upright is 97% and supine is 90%. Vitals are otherwise normal and labs are normal. Jugular venous pressure is at 5cm H2O and lung exam has dullness to percussion in the left base. Spirometry is consistent with a restrictive picture, FEV1/FVC ratio of 1.00. What is the cause of the dyspnea?   1. Heart failure exacerbation 2. Pulmonary embolism 3. Pneumonia 4. Paralyzed left hemidiaphragm   Q33 | An immunocompetent 25 year old patient is intubated with ARDS secondary to necrotizing alcoholic pancreatitis. He is started on appropriate broad anti-bacterial agents and fluid resuscitation. His sputum culture has light growth candida species and chest XR shows persistent bilateral infiltrates. What is the next best step in management of the sputum culture findings?   1. Itraconazole 2. Fluconazole 3. Anidulafungin 4. No antifungal drug treatment   Q34 |
| A patient with status asthmaticus is intubated after symptoms were refractory to appropriate initial management. In addition to continuing the albuterol and steroids and proper sedation, which of the following treatments is most appropriate at this time?   1. Ketamine 2. Benadryl 3. Naproxen 4. Sodium bicarbonate   Q35 | A patient is interested in smoking cessation. Which of the following considerations need to be assessed before starting varenicline?   1. Cancer history 2. Pulmonary hypertension 3. Liver disease 4. Kidney disease   Q36 | A young patient with no medical history is involved in a motor vehicle crash leading to a humerus fracture. He is brought to the OR and is transfused 2 liters of normal saline and 1 unit of packed RBC. He is extubated and brought to the PACU in stable condition. 3 hours later he has acute onset of hypoxemia requiring re-intubation. Chest XR has new patchy bilateral alveolar infiltrates. What is the cause of his respiratory failure?   1. Fat embolism 2. Congestive heart failure and volume overload 3. Transfusion-associated acute lung injury 4. Myocardial infarction   Q37 | A patient is hospitalized with a community acquired pneumonia and treated with 3 days of ceftriaxone and azithromycin before being discharged with a fluoroquinolone to finish a 7 day course. 6 weeks later she has full resolution of symptoms and normal vitals. What is the next best step?   1. Chest XR 2. Chest CT 3. C-reactive protein and erythrocyte sedimentation rate levels 4. Pro-calcitonin testing 5. No additional testing   Q38 |
| A patient with recent diagnosis of right sided breast cancer was treated with lumpectomy and radiation which was completed 8 weeks ago. She presents with dry cough, pleuritic pain, dyspnea and was treated for 7 days with moxifloxacin without improvement. Chest XR demonstrates a right lower lobe opacity. Her vitals are normal and labs are unremarkable except for WBC 11k. TTE is normal. What is the best next step in management?   1. Lasix 2. Ceftriaxone and Azithromycin 3. Prednisone 4. Fluconazole   Q39 | A 20 year old female is on maximal asthma therapy with good compliance yet has had repeated episodes of asthma exacerbations. She has been intubated twice and has needed many courses of prednisone bursts. The attacks are triggered by stress, dust, cigarette smoke, exercise without seasonal variations. Her vitals are normal and she says she feels throat tightness occasionally. Spirometry demonstrates FEV1 95% predicted and FVC 100% predicted with FEV1/FVC 0.95. Lung volumes and diffusion capacity are normal. Chest XR is normal. What is the best next step?   1. Chest CT 2. Transthoracic echocardiogram 3. Repeat spirometry with a flow volume loop 4. Start an anxiolytic   Q40 | A patient with asthma and chronic rhinosinusitis has an asthma exacerbation after starting over the counter aspirin and ibuprofen for sinus headaches and congestion. Within 2 hours he started having facial flushing, rhinorrhea, conjunctival injection and worsening dyspnea. He was treated with prednisone and albuterol nebulizers with improvement. What else would benefit this patient in addition to discontinuation of the NSAIDs and Aspirin?   1. Azithromycin 2. Tiotropium 3. Anti-IgE therapy 4. Montelukast   Q41 | In a patient with hemoptysis, how should they be positioned?   1. Bad lung down 2. Bad lung up 3. Supine 4. Prone 5. 60 degrees head of the bed incline   Q42 |
| Modifier | Modifier | Modifier | Modifier |
| Modifier | Modifier | Modifier | Modifier |
| Modifier | Modifier | Modifier | Modifier |
| Modifier | Modifier | Modifier | Modifier |
| A 50 year old female presents with a left sided pneumonia and is intubated for hypoxic respiratory failure. Capnography waveform is normal, colorimetric end tidal CO2 device has a color change, the endotracheal tube is at 25cm at the teeth. Pre intubation SpO2 is 82% and post intubation SpO2 is 85% on 100% FiO2 and PEEP 10. There are reduced lung sounds on the left. What is the next best step?   1. Needle decompression of the left chest 2. Increase PEEP 3. Increase respiratory rate 4. Retract the endotracheal tube by 3-5cm 5. Broaden antibiotics   Q43 | Which of the following is the best method for diagnosis of idiopathic pulmonary fibrosis?   1. Bronchoscopy with transbronchial biopsy 2. Transesophageal echocardiogram 3. High resolution chest CT scan 4. Chest CT scan with IV contrast 5. Pulmonary function testing   Q44 | Pulmonary involvement can occur in c-antineutrophil cytoplasmic antibody mediated diseases. Which of the following is a c-ANCA associated disease?   1. Granulomatosis with polyangiitis 2. Rheumatoid arthritis 3. Sjogren’s disease 4. Primary sclerosing cholangitis 5. Polyarteritis nodosa   Q45 | The patient has a peripherally located spiculated nodule and a right sided pleural effusion. A thoracentesis is performed and 20ml are sent with exudative fluid studies and negative cytology. What is the next best step for diagnosis?   1. Sputum cultures 2. Bronchoscopy with lavage 3. Repeat thoracentesis up to three times with cytology 4. High resolution CT scan 5. Followup in 3-6 months   Q46 |
| A patient presents with acute shortness of breath and a cardiac point of care ultrasound demonstrates right ventricular enlargement with akinesis of the mid free wall and a more dynamic RV apex. Lung POCUS demonstrates normal lung sliding and A lines. What pathology do these findings suggest?   1. Pneumothorax 2. Pulmonary embolism 3. Congestive heart failure 4. Pneumonia 5. Acute respiratory distress syndrome (ARDS)   Q47 | In lung point of care ultrasound, an anechoic space located superior to the diaphragm indicates which of the following:   1. Pneumothorax 2. Pulmonary edema 3. Consolidation 4. Emphysema 5. Pleural effusion   Q48 | A thoracentesis is performed and the fluid studies demonstrates Protein 3.5, Glucose 12, LDH 1250, cell count 1000 / uL with 88% lymphocytes. What is the most likely etiology of the pleural effusion?   1. Empyema 2. Pulmonary embolism 3. Pancreatitis 4. Rheumatoid pleurisy 5. Congestive heart failure 6. Nephrotic syndrome   Q49 | A patient with a severe asthma exacerbation has refractory symptoms despite albuterol, IV methylprednisolone, and escalating oxygen support. Which of the following would be most appropriate at this time?   1. Epinephrine 2. Budesonide 3. IV Phosphate 4. IV Immunoglobulin 5. Hydroxyzine   Q50 |
|  |  |  |  |
|  |  |  |  |
| Modifier | Modifier | Modifier | Modifier |
| Modifier | Modifier | Modifier | Modifier |
|  |  |  |  |
|  |  |  |  |
