# Supplementary material for: Pulmonopoly: A Game-Based Approach to Teach and Reinforce Basic Concepts of Pulmonary Medicine to Medical Students
Source: MedEdPORTAL. 2025 Feb 21;21:11493. doi: 10.15766/mep_2374-8265.11493 (PMC11842520; doi:10.15766/mep_2374-8265.11493)
Supplement: Supplementary file 1 — Pulmonopoly Board.pdfQuestion Cards.docxProperty Cards, Modifier Cards, and Player Pieces.pdfQuestion and Answer Key.docxGame Rules.docxPre- and Postintervention Surveys.docx [file mep_2374-8265.11493-s001.zip › F. Pre- and Postintervention Surveys.docx]

**Appendix F: Pre- and PostIntervention Surveys**

**Pre-Intervention Survey (Scale from 1-5, 1=Strongly Disagree, 2=Disagree, 3=Neutral, 4= Agree, 5=Strongly Agree)**

1. I have a good understanding of the core concepts of obstructive pulmonary physiology.
2. I have a good understanding of the core concepts of restrictive pulmonary physiology.
3. I have a good understanding of the core concepts of acid/base pulmonary physiology.
4. I have a good understanding of the core concepts of ventilation/perfusion pulmonary physiology.
5. I have a good understanding of the core concepts of obstructive pulmonary pathophysiology.
6. I have a good understanding of the core concepts of restrictive pulmonary pathophysiology.
7. I have a good understanding of the core concepts of acid/base pulmonary pathophysiology.
8. I have a good understanding of the core concepts of ventilation/perfusion pulmonary pathophysiology.
9. I have a good understanding of the core concepts of inhaler pharmacology.
10. I have a good understanding of the core concepts of smoking cessation medications.
11. I have a good understanding of the core concepts of pulmonary anatomy.

**Post-Intervention Survey (Scale from 1-5, 1=Strongly Disagree, 2=Disagree, 3=Neutral, 4= Agree, 5=Strongly Agree)**

1. Playing the game *Pulmonopoly* helped reinforce the core concepts of obstructive pulmonary physiology.
2. Playing the game *Pulmonopoly* helped reinforce the core concepts of restrictive pulmonary physiology.
3. Playing the game *Pulmonopoly* helped reinforce the core concepts of acid/base pulmonary physiology.
4. Playing the game *Pulmonopoly* helped reinforce the core concepts of ventilation/perfusion pulmonary physiology.
5. Playing the game *Pulmonopoly* helped reinforce the core concepts of obstructive pulmonary pathophysiology.
6. Playing the game *Pulmonopoly* helped reinforce the core concepts of restrictive pulmonary pathophysiology.
7. Playing the game *Pulmonopoly* helped reinforce the core concepts of acid/base pulmonary pathophysiology.
8. Playing the game *Pulmonopoly* helped reinforce the core concepts of ventilation/perfusion pulmonary pathophysiology.
9. Playing the game *Pulmonopoly* helped reinforce the core concepts of inhaler pharmacology.
10. Playing the game *Pulmonopoly* helped reinforce the core concepts of smoking cessation medications.
11. Playing the game *Pulmonopoly* helped reinforce the core concepts of pulmonary anatomy.
