# Supplementary material for: Pulmonopoly: A Game-Based Approach to Teach and Reinforce Basic Concepts of Pulmonary Medicine to Medical Students
Source: MedEdPORTAL. 2025 Feb 21;21:11493. doi: 10.15766/mep_2374-8265.11493 (PMC11842520; doi:10.15766/mep_2374-8265.11493)
Supplement: Supplementary file 1 — Pulmonopoly Board.pdfQuestion Cards.docxProperty Cards, Modifier Cards, and Player Pieces.pdfQuestion and Answer Key.docxGame Rules.docxPre- and Postintervention Surveys.docx [file mep_2374-8265.11493-s001.zip › D. Question and Answer Key.docx]

**Pulmonopoly Question Bank Answer Sheet**

**(Anatomy/Pharmacology, Physiology, Pathophysiology, Modifier) 50 questions per section**

**Anatomy/Pharmacology**

Anatomy/Pharmacology Q1

What defines a “true” rib?

1. The rib attaches directly to the sternum
2. The rib is developed from the embryonic mesoderm
3. The rib is attached to the thoracic vertebrae
4. The rib forms the costal margin

ANSWER: a. The rib attaches directly to the sternum

Anatomy/Pharmacology Q2

How many “false ribs” are there?

ANSWER: 5 false ribs that have no direct attachment to the sternum

Anatomy/Pharmacology Q3

The neurovascular bundles run along the ______ aspect of the rib.

ANSWER: inferior

Anatomy/Pharmacology Q4

What are the three components that make up the sternum?

ANSWER: manubrium, body, xiphoid process

Anatomy/Pharmacology Q5

At what thoracic level does the inferior vena cava pass through the diaphragm?

ANSWER: T8 level

Anatomy/Pharmacology Q6

At what thoracic level does the esophagus pass through the diaphragm?

ANSWER: T10 level

Anatomy/Pharmacology Q7

At what thoracic level does the aorta pass posterior to the diaphragm?

ANSWER: T12 level

Anatomy/Pharmacology Q8

What do you call the paradoxical movement of the thoracic wall that occurs in the setting of multiple rib fractures?

ANSWER: Flail Chest

Anatomy/Pharmacology Q9

The neurovascular bundles travel in costal grooves in a vertical arrangement. What is the typical order of these structures from superior to inferior?

1. Nerve (superior) – Artery (middle) – Vein (inferior)
2. Artery – Nerve – Vein
3. Vein – Artery – Nerve
4. Nerve – Vein – Artery

ANSWER: c. Vein – Artery – Nerve

Anatomy/Pharmacology Q10

Venous blood from the thoracic wall returns to the heart via the thoracic veins and the _______?

1. Azygos system
2. Mesenteric system
3. Portal system
4. Vertebral system

ANSWER: a. Azygos system

Anatomy/Pharmacology Q11

Arterial blood supply to the thoracic wall originates from the internal thoracic arteries and ______?

1. Ascending aorta
2. Descending aorta
3. Subclavian artery
4. Vertebral artery

ANSWER: b. Descending aorta

Anatomy/Pharmacology Q12

To trigger inspiration does intrathoracic pressure increase or decrease?

ANSWER: Decrease of intrathoracic pressure triggers inspiration

As the diaphgram drops, the volume of the thoracic cavity increases and intrathoracic pressure decreases, which leads to inspiration of air.

Anatomy/Pharmacology Q13

Which nerve innervates the diaphragm?

ANSWER: phrenic nerve

Anatomy/Pharmacology Q14

What is the term that describes an accumulation of lymph in the pleural cavity?

ANSWER: Chylothorax

Anatomy/Pharmacology Q15

What is the term that describes accumulation of air and serous fluid in the pleural cavity?

ANSWER: hydropneumothorax

Anatomy/Pharmacology Q16

How many total lung lobes are there?

ANSWER: 5

Anatomy/Pharmacology Q17

Parasympathetic innervation of the lungs comes primarily from which nerve?

ANSWER: Vagus nerve

Anatomy/Pharmacology Q18

Respiratory centers of the brain that initiate breathing are located in which region?

1. Cerebral cortex
2. Amygdala
3. Hypothalamus
4. Medulla Oblongata

ANSWER: d. Medulla Oblongata

Anatomy/Pharmacology Q19

The reflex to cough in response to chemical or mechanical irritants in the lungs is primarily mediated by which nerve?

1. Hypoglossal nerve
2. Vagus nerve
3. Facial nerve
4. Thoracic nerves

ANSWER: b. Vagus nerve

Anatomy/Pharmacology Q20

Arterial chemoreceptors that respond to low pH and PaO2 are found in “bodies” located near which of the following vessels?

1. Pulmonary artery
2. Subclavian artery
3. Carotid artery
4. Descending aorta

ANSWER: c. Carotid artery

Anatomy/Pharmacology Q21

The main mechanism of movement of pleural fluid out of the pleural space is via

1. Venous recirculation
2. Lymphatic recirculation
3. Osmosis through the parietal pleural membrane
4. Absorption from resident macrophages and phagocytes

ANSWER: b. Lymphatic recirculation

Anatomy/Pharmacology Q22

The respiratory system is derived from the:

1. Embryonic gut
2. Embryonic neural tube
3. Embryonic heart
4. Yolk sac

ANSWER: a. Embryonic gut

Anatomy/Pharmacology Q23

Which of the following is a complication of tracheo-esophageal fistulas in fetal development?

1. Intra-uterine growth restriction
2. Gestational diabetes
3. Pre-eclampsia
4. Polyhydramnios

ANSWER: d. Polyhydramnios

Anatomy/Pharmacology Q24

Alpha-1 anti-trypsin deficiency associated emphysema is typically:

1. Upper lobe predominant
2. Lower lobe predominant
3. Centrilobular predominant
4. Right lower lobe predominant

ANSWER: b. Lower lobe predominant

Alpha-1 anti-trypsin deficiency related lung disease often presents in young patients in their 30’s and 40’s. The pattern is most often a panacinar emphysema most pronounced in the lung bases. However, diffuse and upper lobe emphysema still can occur.

Anatomy/Pharmacology Q25

A patient with cirrhosis has a complication of hydrothorax. This fluid collection is usually located in the:

1. Left pleural space
2. Right pleural space
3. Bilateral pleural spaces
4. Mediastinum

ANSWER: b. Right pleural space

Cirrhosis and portal hypertension cause the accumulation of fluid in the pleural space, as well as the peritoneal space. The cause of the hydrothorax is thought to be due to small defects in the diaphragm that allow for direct passage of ascitic fluid. Other contributing factors include hypoalbuminemia, lymphatic drainage across the diaphragm, and azygos vein hypertension.

Anatomy/Pharmacology Q26

Sarcoidosis is diagnosed by (1) typical clinical and radiological manifestations, (2) the presence of noncaseating granulomas, and (3) exclusion of alternative diseases. In the case of pulmonary sarcoidosis, which lymph nodes are classically involved?

1. Sub-pleural
2. Lower lobe
3. Peripheral
4. Tracheal
5. Hilar

ANSWER: e. Hilar

Some studies suggest that as high as 75-90% of patients with sarcoidosis will have mediastinal and hilar lymphadenopathy.

Anatomy/Pharmacology Q27

Rheumatoid arthritis related lung fibrosis often affects which areas of the lung preferentially?

1. Upper lobes
2. Hilar
3. Tracheal
4. Basilar and subpleural
5. Right lower lobe

ANSWER: d. Basilar and subpleural

Rheumatoid arthritis related lung fibrosis can be diagnosed by high resolution CT (HRCT) imaging. These interstitial lung disease findings on HRCT are often in a usual insterstitial pneumonia (UIP) or nonspecific interstitial pneumonia (NSIP) pattern with fibrotic changes predominantly in the basilar and subpleural areas.

Anatomy/Pharmacology Q28

What is the mechanism of action of albuterol?

1. Beta 2 antagonist
2. Beta 2 agonist
3. Beta 1 antagonist
4. Beta 1 agonist

ANSWER: Beta-2 agonist

Beta-2 agonists stimulate the adrenergic receptors, increase cyclic AMP, and lead to airway smooth muscle relaxation and bronchodilation.

Anatomy/Pharmacology Q29

What is the mechanism of action of Omalizumab, for treatment of asthma?

1. Monoclonal antibody to IgE
2. Monoclonal antibody to IL-5
3. Monoclonal antibody to IL-4
4. Monoclonal antibody to IL-13

ANSWER: a. Monoclonal antibody to IgE

Omalizumab inhibits binding of IgE to receptors on basophils and mast cells, which decreases cellular and cytokine responses that contribute to allergic responses. Thus, omalizumab is indicated for severe allergic asthma.

Anatomy/Pharmacology Q30

This class of medication targets the parasympathetic nervous system to relax airways

1. Beta 2 agonist
2. Anti-cholinergic
3. Inhaled corticosteroid
4. Alpha 2 agonist

ANSWER: b. Anti-cholinergic

Inhaled anti-cholinergic medications such as tiotropium and ipratropium block the parasympathetic responses that cause bronchoconstriction. The muscarinic receptor antagonists inhibit acetylcholine mediated bronchospasm by blocking the muscarinic receptors in the airways.

Anatomy/Pharmacology Q31

Roflumilast is a medication used to decrease exacerbations in severe COPD, what is its mechanism of action?

1. Anti-cholinergic
2. Inhaled corticosteroid
3. Phosphodiesterase 4 inhibitor
4. Monoclonal antibody to IL-5

ANSWER: c. Phosphodiesterase 4 inhibitor

Roflumilast’s actions lead to a decrease in the inflammatory pathways underlying the pathogenesis of COPD and emphysema.

Anatomy/Pharmacology Q32

What type of adrenergic receptor action leads to bronchorelaxation?

1. Beta 1 agonist
2. Beta 2 agonist
3. Alpha 1 agonist
4. Beta 1 antagonist

ANSWER: b. Beta 2 agonist

Beta-2 agonists stimulate the adrenergic receptors, increase cyclic AMP, and lead to airway smooth muscle relaxation and bronchodilation.

Anatomy/Pharmacology Q33

A patient with pulmonary lymphoma is being treated with a regimen including rituximab. Which of the following is true about its mechanism of action?

1. Binds to soluble and transmembranous forms of TNF-alpha
2. Binds to CD20 receptors on cell surfaces
3. Binds to BCR-ABL receptors on cell surfaces
4. Inhibits topoisomerase II activity

ANSWER: b. binds to CD20 receptors on cell surfaces

Rituximab leads to death of B lymphocytes by antibody dependent cellular cytotoxicity, complement mediated cytotoxicity, and apoptosis.

Anatomy/Pharmacology Q34

Which of the following are NOT drugs used for treatment of tuberculosis?

1. Isoniazid
2. Ethambutol
3. Levofloxacin
4. Streptomycin
5. Piperacillin

ANSWER: e. Piperacillin

The other drugs are all used for treatment of tuberculosis. Of note, streptomycin was the first effective anti-tuberculosis drug but is less commonly used in regimens now because it requires delivery by intramuscular injection.

Anatomy/Pharmacology Q35

Which of the following is true about treatment of active tuberculosis?

1. Standard of care is treatment with 2 drugs for 9 months
2. Standard of care is treatment with 4 drugs for 2 months, then 2 drugs for 4 months
3. You do not need to avoid drugs that the patient has been exposed to in the past
4. Extensively drug resistant TB is defined as resistance to only a fluoroquinolone

ANSWER: b. Standard of care is treatment with 4 drugs for 2 months, then 2 drugs for 4 months

The RIPE TB treatment regimen uses Rifampin, Isoniazid, Pyrazinamide, and Ethambutol.

Anatomy/Pharmacology Q36

In treatment of Tuberculosis, what supplement is given with Isoniazid to prevent peripheral neuropathy side effects?

1. Vitamin B1
2. Vitamin B6
3. Thiamine
4. Folic acid

ANSWER: b. Vitamin B6

Anatomy/Pharmacology Q37

Which of the following treatments of Tuberculosis lead to a yellow-orange color to urine, sweat, tears in the patient?

1. Ethambutol
2. Pyrazinamide
3. Rifampin
4. Isoniazid

ANSWER: c. Rifampin

The effect is harmless and usually will resolve with discontinuation of the medication, however the discoloration could be permanent.

Anatomy/Pharmacology Q38

When covering for vancomycin resistant organisms, such as vancomycin resistant enterococcus, which of the following drugs does not have good lung penetration?

1. Linezolid
2. Doxycycline
3. Daptomycin
4. Rifampin

ANSWER: c. Daptomycin

Anatomy/Pharmacology Q39

A PCP is discussing Bupropion for treatment of nicotine addiction. Which of the following are notable side effects of Bupropion?

1. Lowered seizure threshold
2. Depression
3. Anhedonia
4. Weight gain

ANSWER: a. Lowered seizure threshold

Anatomy/Pharmacology Q40

Which of the following is the most effective studied pharmacotherapy for nicotine addiction?

1. Nicotine patch or gum replacement therapy
2. Buproprion
3. Varenicline
4. Selective serotonin reuptake inhibitors

ANSWER: c. Varenicline

Varenicline (Chantix) is more efficacious than buproprion or nicotine replacement therapy for smoking cessation. SSRI’s are not indicated for smoking cessation.

Anatomy/Pharmacology Q41

What is the mechanism of Varenicline for treatment of nicotine addiction?

1. Dual inhibition of norepinephrine and dopamine reuptake
2. Partial agonist of alpha 4 beta 2 subtype nicotinic cholinergic receptor
3. Selective serotonin reuptake inhibitor
4. Partial agonist of dopamine receptors

ANSWER: b. Partial agonist of alpha 4 beta 2 subtype nicotinic cholinergic receptor

Varenicline’s (Chantix) actions on the receptor results in a dopamine release that is approximately half of that released by the pure agonist nicotine. It also has a long half-life and prevents the receptors from being further stimulated by nicotine.

Anatomy/Pharmacology Q42

Which of the following is the preferred pharmacotherapy for long term anticoagulation for a pregnant patient with pulmonary embolism?

1. Warfarin
2. Unfractionated heparin
3. Rivaroxiban
4. Low molecular weight heparin

ANSWER: d. Low molecular weight heparin

Warfarin is contraindicated in pregnancy. Unfractionated heparin is not practical because it requires a continuous infusion. Rivaroxiban and other DOACs are associated with higher rate of fetal abnormality and loss compared with low molecular weight heparin.

Anatomy/Pharmacology Q43

A patient hospitalized with COVID-19 is started on Remdesivir. What is the mechanism of action of remdesivir?

1. Adenosine analog that inhibits RNA-dependent RNA polymerase to inhibit viral replication
2. Spike protein antibody mediated destruction
3. Integrase inhibitor to inhibit viral replication
4. Reverse transcriptase inhibitor to inhibit viral DNA replication

ANSWER: a. Adenosine analog that inhibits RNA-dependent RNA polymerase to inhibit viral replication

Anatomy/Pharmacology Q44

A patient is being treated with oseltamivir for influenza. What is the mechanism of action?

1. Neuraminidase inhibitor to prevent release of budding viral progeny from the cell membrane
2. Fusion and entry inhibitor to prevent viral entry into the cell
3. Reverse transcriptase inhibitor to prevent viral replication
4. Integrase inhibitor to prevent viral replication within the nucleus

ANSWER: a. Neuraminidase inhibitor to prevent release of budding viral progeny from the cell membrane

Anatomy/Pharmacology Q45

What is the recommended time frame from symptom onset for treatment of influenza with antiviral therapy?

1. 24 hours
2. 48 hours
3. 72 hours
4. 96 hours

ANSWER: b. 48 hours

Late initiation of oseltamivir beyond 48 hours had minimal effect on symptoms.

Anatomy/Pharmacology Q46

First line treatment for a patient diagnosed with acute allergic bronchopulmonary aspergillosis includes glucocorticoids and ______ .

1. Amphotericin B
2. Anidulafungin
3. Rifampin and Isoniazid
4. Ceftriaxone and Azithromycin
5. Itraconazole

ANSWER: e. Itraconazole

Amphotericin B is used for anti-fungal coverage when there is treatment failure.

Anatomy/Pharmacology Q47

Patients with cystic fibrosis should use several treatments to enhance airway clearance. Which of the following is NOT a typical agent used for this?

1. Inhaled Albuterol
2. Inhaled Hypertonic saline
3. Inhaled DNase
4. Inhaled Mannitol
5. All of the above are typical agents for enhancing airway clearance in CF patients

ANSWER: e. All of the above are typical agents for enhancing airway clearance in CF patients

Anatomy/Pharmacology Q48

A patient with cystic fibrosis is admitted with concern for pneumonia. Which of the following antibiotics would not be appropriate?

1. Cefepime
2. Ceftriaxone
3. Ciprofloxacin
4. Meropenem

ANSWER: b. Ceftriaxone

Pseudomonas is a very prelavent organism in patients with cystic fibrosis. Of these antibiotics, Ceftriaxone is the only one that does not have pseudomonal coverage.

Anatomy/Pharmacology Q49

Which of the following treatments does not have benefit in acute COPD exacerbation?

1. Intravenous magnesium
2. Albuterol-ipratropium
3. Methylprednisolone
4. Azithromycin

ANSWER: a. Intravenous magnesium

IV magnesium is a treatment for acute asthma exacerbation and is believed to have benefit due to anti-inflammatory and bronchodilating effects. Studies have not demonstrated a significant benefit for using IV magnesium in COPD exacerbations.

Anatomy/Pharmacology Q50

Which of the following can increase the risk of pneumonia in patients with COPD?

1. Inhaled Tiotropium
2. Inhaled Albuterol
3. Inhaled Fluticasone
4. Inhaled Heli-ox
5. Montelukast

ANSWER: c. Inhaled Fluticasone

As a class, inhaled corticosteroids increase the risk of pneumonia in patients with COPD.

**Physiology**

Physiology Q1

Which of the following is NOT an etiology for transudative pleural effusions?

1. Heart failure
2. Decompensated liver failure
3. Malignancy
4. Atelectasis

ANSWER: c. Malignancy

Transudative pleural effusions are caused by increased intravascular hydrostatic pressure or decreased oncotic pressure leading to fluid moving to extravascular spaces. Malignancy is commonly associated with exudative effusions due to effects of the cancer cells disrupting the permeability of the layer and also causing an inflammatory mileau.

Physiology Q2

Which of the following mechanisms contributes to development of pleural effusions?

1. Decreased pleural pressure
2. Increased plasma oncotic pressure
3. Decreased microvascular pressure
4. Decreased membrane permeability

ANSWER: a. decreased pleural pressure

The difference in pressure (hydrostatic and oncotic) between the intravascular and extravascular spaces dictate the movement of fluid.

Physiology Q3

Which of the following is NOT part of Light’s Criteria for exudative pleural effusions?

1. Pleural fluid protein divided by serum protein is greater than 0.5
2. Pleural fluid LDH divided by serum LDH is greater than 0.6
3. Pleural fluid protein is greater than two-thirds the upper limit of normal for serum protein
4. Pleural fluid LDH is greater than two-thirds the upper limit of normal for serum LDH

ANSWER: c. Pleural fluid protein is greater than two-thirds the upper limit of normal for serum protein. This criteria had a diagnostic sensitivity of 99% and specificity of 98% for exudative effusions.

Physiology Q4

Hypoxemic respiratory failure is defined as:

1. Pulse oximetry below 94% on room air
2. PaO2 below 60 mmHg on room air
3. PaCO2 greater than 50 on room air
4. PH less than 7.3 with PaO2 below 70 mmHg on room air

ANSWER: b. PaO2 below 60 mmHg on room air. PaO2 is the partial pressure of oxygen in the arterial blood. Measurement of PaO2 is the gold standard for diagnosis of hypoxemic respiratory failure. However peripheral oxygen saturation (SpO2) can be measured non-invasively and a PaO2 of 60 mmHg correlates with an SpO2 of 90%.

Physiology Q5

The A-a gradient is defined as:

1. Partial pressure of arterial O2 – partial pressure of alveolar O2
2. Partial pressure of arterial O2 – fraction of inspired oxygen
3. Partial pressure of alveolar O2 – partial pressure of arterial O2
4. Partial pressure of alveolar O2 – arterial concentration of carbon dioxide

ANSWER: c. Partial pressure of alveolar O2 – partial pressure of arterial O2. This difference can help narrow the differential for hypoxemia because certain etiologies will not have an elevated A-a gradient.

Physiology Q6

Which of the following etiologies of hypoxemia would still have a normal A-a gradient?

1. Ventilation – perfusion mismatch
2. Hypoventilation
3. Diffusion abnormality
4. Shunt

ANSWER: b. Hypoventilation

This is because the partial pressure of oxygen is decreased similar in both the arteries and alveoli in hypoventilation and thus there will not be an elevation in the gradient. Causes of alveolar hypoventilation can be due to decreased respiratory drive, neuromuscular disease, mechanical restriction due to trauma.

Physiology Q7

A patient with aero-hypoxia [(ex) in high altitude] has a normal A-a gradient?

1. True
2. False

ANSWER: a. True

Physiology Q8

Oxygen therapy does not help in which etiologies of hypoxemia?

1. Aero-hypoxia, such as in high altitude
2. Diffusion abnormality
3. Shunt
4. Ventilation – perfusion mismatch

ANSWER: c. Shunt

A shunt leads to gas exchanging alveolar units with no blood flow. So increasing the oxygen in those alveoli does not lead to a change in hypoxemia because the gas exchange is not happening at all.

Physiology Q9

Nicotine binds nicotinic cholinergic receptors, especially alpha 4 beta 2, and this binding leads to:

1. Glutamate release which promotes dopamine release
2. Upregulation of monoamine oxidases
3. GABA release and upregulation
4. Inhibition of NMDA

ANSWER: a. Glutamate release which promotes dopamine release

This dopamine release in the mesolimbic area, the corpus striatum, and the frontal cortex lead to the pleasure, mood changes, stimulation, and cravings associated with nicotine addiction.

Physiology Q10

In a patient with massive pulmonary embolism, transthoracic echocardiography can demonstrate McConnell’s sign. Which of these is characteristic of this sign?

1. Tricuspid regurgitation
2. Regional wall motion abnormality in the right ventricle that spares the apex
3. Left ventricular hypokinesis
4. Right atrial dilation and hypokinesis

ANSWER: b. Regional wall motion abnormality in the right ventricle that spares the apex. McConnell’s sign has a low sensitivity but a high specificity (97%+) for pulmonary embolism.

Physiology Q11

A potential EKG finding associated with patients with pulmonary embolism is:

1. S1 Q3 T3
2. Q waves in II, III, aVF
3. Left axis deviation
4. S waves in V1 and R waves in V6 summing to > 35 mm

ANSWER: a. S1 Q3 T3

Though often taught as a classic EKG finding for pulmonary embolism, studies have shown that it is neither sensitive or specific.

Physiology Q12

A medical students auscultates for vocal resonance as a patient says “ninety-nine”. Which of the following is true?

1. There should be more resonance with pleural effusion
2. There should be more resonance with consolidation
3. There should be more resonance with bronchospasm
4. There should be more resonance with pneumothorax

ANSWER: b. There should be more resonance with consolidation

Sound waves travel faster through more dense material. Thus when listening on exam, consolidation will transmit the sound waves better than aerated lung tissue and lead to resonance.

Physiology Q13

How do alpha 1 anti-tryptase and other proteolytic enzymes protect the lung in patients who smoke cigarettes?

1. Proteolytic enzymes stimulate alveolar macrophages to phagocytose cigarette toxin deposits
2. Proteolytic enzymes cleave nicotine from its receptors
3. Nicotine increases neutrophil secretion of elastase. Proteolytic enzymes inhibit elastase and prevent lung tissue destruction and development of emphysema
4. Proteolytic enzymes increase surfactant production and prevents surfactant degradation

ANSWER: c. Nicotine increases neutrophil secretion of elastase. Proteolytic enzymes inhibit elastase and prevent lung tissue destruction and development of emphysema.

Alpha-1 antitrypsin deficiency leads to destruction of lung tissue and emphysematous changes. Often this will be seen as a young patient presenting with findings of COPD.

Physiology Q14

Which of the following is part of management of flash pulmonary edema due to hypertensive emergency?

1. Increase cardiac preload
2. Decrease cardiac afterload
3. Increase cardiac contractility
4. Hyperventilation

ANSWER: b. Decrease cardiac afterload

Decreasing both cardiac preload and afterload is important in the management of flash pulmonary edema due to hypertensive emergency, often with the use of an IV nitrate. Non-invasive positive pressure ventilation also is beneficial.

Physiology Q15

How does nephrotic syndrome lead to development of a pleural effusion?

1. Increased RAAS blockade
2. Decreased intravascular oncotic pressure
3. Decreased intravascular hydostatic pressure
4. Increased protein accumulation in the pleural space

ANSWER: b. Decreased intravascular oncotic pressure

Nephrotic syndrome is characterized by significant protein loss, which leads to decreased intravascular oncotic pressure. Third-spacing of the intravascular fluid will lead to a transudative pleural effusion.

Physiology Q16

A 30 year old patient is sedated with fentanyl and midazolam and given local anesthetic and intubated for a procedure. 5 minutes later, his vitals read T 98, HR 108, BP 120/80, SpO2 82% (previously was 98%). Breath sounds are clear bilaterally. Blood gas demonstrates pH 7.38, PaO2 115, PaCO2 44. What is the diagnosis?

1. Intubation of the right mainstem bronchus
2. Pulmonary embolism
3. Opioid overdose
4. Acute respiratory distress syndrome
5. Methemoglobinemia

ANSWER: e. methemoglobinemia (secondary to the oxidizing local anesthetic).

High levels of methemoglobin will decrease oxygen carrying capacity of blood and shift the oxygen dissociation curve to the left. This leads to a functional anemia and tissue hypoxia. It will also cause a falsely low SpO2 reading, even when the PaO2 is high.

Physiology Q17

A 25 year old female presents to pulmonology clinic with shortness of breath on exertion. Vitals are normal and lungs are clear at this time. Her symptoms are episodic and mild, occur 1 time per week, do not wake her from sleep. Spirometry data demonstrates:

Prebronchodilator: FEV1/FVC ratio 55%

Postbronchodilator: FEV1/FVC ratio 82%

What is the best therapy to start her on?

1. Fluticasone daily
2. Budesonide-formoterol as needed
3. Ipratropium as needed
4. Anti-IgE
5. Umeclidinium-vilanterol daily

ANSWER: b. Budenoside-formoterol as needed

This patient meets criteria for intermittent asthma, as supported by her symptoms and the obstructive pattern on PFTs with a bronchodilator response. The best therapy at this time is a LABA-ICS, thus b is the correct answer.

Physiology Q18

A 55 year old female with alcoholic cirrhosis is in the hospital with ascites and encephalopathy. ABG demonstrates pH 7.47 PaO2 105 PaCO2 27 with bicarbonate 20 and anion gap 12. What is the cause of the primary acid base disorder?

1. Lactic acid accumulation
2. Lactulose mediated diarrhea and bicarbonate losses
3. Spontaneous bacterial peritonitis
4. Central stimulation of ventilation due to progesterone
5. Intracranial hemorrhage

ANSWER: d. Central stimulation of ventilation due to progesterone. The patient has a primary respiratory alkalosis. Chronic respiratory alkalosis is common in cirrhotics and due to progesterone accumulation due to impaired hepatic metabolism, which leads to central hyperventilation.

Physiology Q19

A 30 year old female presents with dyspnea and asthma-like symptoms. She had never had a diagnosis of asthma before and felt like the symptoms started 1 month ago after she painted the interior of her house. Spirometry demonstrates FEV1 85%, FVC 90%, FEV1/FVC 0.85. What is the next best test?

1. High resolution CT of the chest
2. Echocardiogram
3. Methacholine challenge testing
4. Cardiopulmonary rehabilitation
5. Bronchoscopy and bronchoalveolar lavage

ANSWER: c. Methacholine challenge testing. The patient likely has reactive airway dysfunction syndrome (RADS) which is a type of irritant triggered asthma. Though her spirometry was not obstructive at baseline, the methacholine challenge could is a bronchoprovocation test that could help diagnose RADS.

Physiology Q20

A 75 year old man presents with dyspnea. On physical exam there is dullness in the right lower lung, crackles on ausculatation, increased tactile fremitus, and egophany. There is 1+ edema bilaterally and he has a history of heart failure and COPD. What does the chest XR show?

1. Lobar Pneumonia
2. Pleural effusion
3. Pneumothorax
4. Pulmonary edema
5. Pulmonary embolism

ANSWER: a. lobar pneumonia

The increased tactile fremitus and egophany are due to a consolidation from the pneumonia allowing for better transmission of sound waves. In addition, the unilateral rales further supports a lobar pneumonia.

Physiology Q21

In lung point of care ultrasound, “B lines” are vertical “comet tail” hyperechoic lines extending from the pleura and are an artifact that indicate which of the following:

1. Pneumothorax
2. Pulmonary edema
3. Emphysema
4. Pleural effusion

ANSWER: b. Pulmonary edema

Diffuse B lines in the appropriate clinical context (heart failure, volume overload) are consistent with pulmonary edema. Other pathologies can cause focal B-lines such as consolidation and interstitial lung disease.

Physiology Q22

Which form of respiratory support device is most appropriate in management of a COPD exacerbation with hypercarbia?

1. Non-rebreather
2. Continuous positive aiway pressure
3. Bilevel positive airway pressure
4. Nasal cannula

ANSWER: c. Bilevel positive airway pressure

BIPAP will aid in the patient’s ability to ventilate. The difference in inspiratory and expiratory pressures will increase flow and tidal volume and allow the patient to exhale more carbon dioxide.

Physiology Q23

Which of the following is most likely to improve ventilation / perfusion matching?

1. Placing a patient in a prone position
2. Placing a patient in left lateral recumbant position
3. Hyperventilation
4. Moving to higher altitude

ANSWER: a. Placing a patient in a prone position

This position will improve V/Q matching by recruiting more dorsal lung tissue and decreasing dead space.

Physiology Q24

What effect does decreasing intrathoracic pressure have on cardiac pre-load?

1. increased preload
2. decreased preload
3. no effect

ANSWER: a. increased preload

When the diaphragm drops and intrathoracic pressure is decreased, venous return increases and cardiac pre-load increases.

Physiology Q25

What effect will a bicarbonate infusion have on a patient’s respiratory acid-base relationship?

1. increased pCO2
2. decreased pCO2
3. no change in pCO2

ANSWER: a. increased pCO2

Bicarbonate is metabolized to water and CO2. CO2 is eliminated through the lungs. Giving a patient bicarbonate without changing their respiratory function will lead to a respiratory acidosis.

Physiology Q26

A 60 year old woman with COPD presents from nursing home with somnolent mental status. She is found to have pH 7.36, pCO2 62, pO2 69, Bicarbonate 36. What is the acid base relationship?

1. Respiratory acidosis with compensatory metabolic alkalosis
2. Respiratory acidosis without compensatory metabolic alkalosis
3. Metabolic acidosis with compensatory respiratory alkalosis
4. Metabolic acidosis without compensatory respiratory alkalosis
5. Compensated metabolic alkalosis

ANSWER: a. Respiratory acidosis with compensatory metabolic alkalosis

A high pCO2 and low pH suggests that the primary disturbance is an acidosis with a respiratory cause due to accumulation of carbon dioxide. Her bicarb is elevated, consistent with a compensatory metabolic alkalosis.

Physiology Q27

A 24 year old man presents to the ED for severe anxiety after being trapped in an elevator for 4 hours. He is found to have pH 7.58, pCO2 20, pO2 90, Bicarbonate 18. What is the acid base relationship?

1. Respiratory alkalosis with compensatory metabolic acidosis
2. Respiratory alkalosis without compensatory metabolic acidosis
3. Metabolic alkalosis with compensatory respiratory acidosis
4. Metabolic alkalosis without compensatory respiratory acidosis

ANSWER: a. Respiratory alkalosis with compensatory metabolic acidosis

The high pH and decreased pCO2 is consistent with respiratory alkalosis due to a high respiratory rate driven by anxiety. He has breathed off carbon dioxide. The bicarbonate is decreased to compensate.

Physiology Q28
A resident went to Mexico for their honeymoon. He had many Tequila Sunrises but they were mixed with tapwater ice. Uh oh. The next few days were spent on the toilet. He finally went to the ED.

ABG 7.30 / pCO2 32 / pO2 90

Na 132, Chloride 110, Bicarb 16

What is the acid base disorder?

1. High anion gap metabolic acidosis
2. Non-gap metabolic acidosis
3. Respiratory acidosis
4. Metabolic alkalosis
5. Mixed gap-non gap metabolic acidosis

ANSWER: b. non-gap metabolic acidosis. The anion gap is 6. The resident has bicarb losses from the diarrhea. There is appropriate respiratory compensation, by winter’s formula:

1.5 * [Bicarb] + 8 +/- 2 = expected pCO2 32 +/- 2

Physiology Q29
A patient lost her insulin. She has abdominal pain, vomiting, and increased urinary frequency. ABG 7.27 / pCO2 23 / pO2 80. Corrected Na 140, Chloride 100, Bicarb 10, Cr 1.3, BUN 50, Lactate 2. What is the acid base disorder?

1. High anion gap metabolic acidosis
2. Non-gap metabolic acidosis
3. Respiratory acidosis
4. Mixed gap-non gap metabolic acidosis
5. Metabolic acidosis with mixed respiratory acidosis

ANSWER: a. High anion gap metabolic acidosis driven by diabetic ketoacidosis, uremia, and lactic acidosis. Anion gap 30. There is appropriate respiratory compensation and no other acid base disorder

**Winter’s Formula for Respiratory Compensation** = 1.5 * [Bicarb] + 8 +/- 2 = 23 +/- 2

AG measured = 30, expected = 12, Delta AG = 18

Bicarb measured = 10, expected = 24, Delta bicarb = 14

18 / 14 = 1.28 = **Pure high AG metabolic acidosis**

Physiology Q30
The patient ate some questionable fish from Reading Terminal Market. He had significant nausea/vomiting.

ABG 7.52 / pCO2 53 / pO2 90

Corrected Na 155, Chloride 100, Bicarb 45, Cr 3, BUN 60

What is the acid base disorder?

1. Respiratory alkalosis
2. Metabolic alkalosis with appropriate respiratory compensation
3. Metabolic alkalosis with mixed respiratory alkalosis
4. Metabolic alkalosis with mixed respiratory acidosis
5. Mixed metabolic alkalosis and metabolic acidosis

ANSWER: b. Metabolic Alkalosis with appropriate respiratory compensation. Due to vomiting and loss of gastric acids.

Metabolic alkalosis due to Bicarb 45, No anion gap.

Respiratory compensation for metabolic alkalosis:

Expected PaCO2= 40 + 0.6*[Delta Bicarb] = 40 + 0.6*[21] = 53 Measured = 53

Physiology Q31
A patient with history of DM2, CAD, HF, chronic COPD presents with acute viral GI illness with nausea/vomiting/diarrhea.

ABG 7.25 / pCO2 60 / pO2 100

Na 140, Chloride 118, Bicarb 12

What is the acid base disorder?

1. Respiratory acidosis
2. High anion gap metabolic acidosis with respiratory compensation
3. Mixed Respiratory acidosis and metabolic acidosis
4. Respiratory acidosis with appropriate metabolic compensation
5. Mixed high anion gap and non gap metabolic acidosis

ANSWER: c. Mixed respiratory acidosis with metabolic acidosis

1. Chronic Respiratory Acidosis – from the COPD

2. Metabolic Acidosis – from the diarrhea

Expected metabolic compensation for chronic resp acidosis = decreased 4 Bicarb from every 10 of pCO2 over 40

Expected Bicarb compensation = 16

Measured Bicarb = 12 (lower than expected, thus there is a **concurrent metabolic acidosis**)

Physiology Q32
A patient with alcoholic cirrhosis goes into withdrawal and starts retching. He presents to the ED with hematemesis and melena. He is found to be hypotensive, tachycardic, and tachypneic.

ABG 7.1 / pCO2 18 / pO2 80

Na 130, Chloride 90 , Bicarb 10

What is the acid base disorder?

1. Mixed high anion gap and non-gap metabolic acidosis
2. Pure high anion gap metabolic acidosis
3. Primary Respiratory alkalosis with compensatory metabolic acidosis
4. Primary high anion gap metabolic acidosis with mixed respiratory alkalosis

ANSWER: d. Primary high anion gap metabolic acidosis with mixed respiratory alkalosis

1. High anion gap metabolic acidosis due to shock, hypoperfusion, lactic acidosis

2. Respiratory Alkalosis due to tachypnea

PH 7.1, pCO2 18, Anion Gap 30, Bicarb 10 consistent with **metabolic acidosis**

Winter’s Formula 1.5 * Bicarb + 8 +/- 2 = Expected pCO2 23 +/- 2

Measured pCO2 = 18, which indicates **Respiratory alkalosis**!

Delta Anion gap / Delta Bicarb = (30 - 12) / (24 – 10) = 18 / 14 = **1.28**

Delta Delta b/w 1-2 consistent with a pure **high anion gap acidosis**

Physiology Q33

A patient with an acute COPD exacerbation is found to have pneumonia. He is treated with nebulizers, steroids, and antibiotics. An ABG is drawn:

ABG 7.33 / pCO2 70 / pO2 60 with Bicarb 27

What is the acid base disorder?

1. Respiratory acidosis with metabolic acidosis
2. Respiratory acidosis with compensatory metabolic alkalosis
3. Metabolic acidosis with respiratory acidosis
4. Metabolic acidosis with respiratory alkalosis

ANSWER: b. Respiratory acidosis with compensatory metabolic alkalosis

Respiratory acidosis with appropriate metabolic compensation

PH 7.33 with pCO2 70 consistent with **primary respiratory acidosis**

For every 10 pCO2 above normal, expect an increase in Bicarb by 1 mEq. This patient’s pCO2 is 30 above normal, so expected bicarb = 24 + 3. Consistent with his measured Bicarb of 27, thus this is **appropriate metabolic compensation.**

Physiology Q34

A patient was administered 3 boluses of fentanyl and 15 minutes later he was found to have respiratory rate of 5 per minute. A blood gas is drawn and the nurse starts ventilating for him with the bag valve mask.

ABG 7.1 /pCO2 70 / pO2 45 with Bicarb 24

What is the acid base disorder?

1. Respiratory acidosis with metabolic compensation
2. Respiratory acidosis without metabolic compensation
3. Metabolic acidosis with mixed respiratory acidosis
4. Metabolic alkalosis with mixed respiratory acidosis

ANSWER: b. Respiratory acidosis without metabolic compensation.

PH 7.1 and pCO2 70 consistent with **primary respiratory acidosis**.

The patient had an iatrogenic opiate overdose that suppressed his respiratory drive leading to hypoventilation, and thus a primary respiratory acidosis. This was an acute process and there was **no time for any metabolic compensatory mechanisms** thus his bicarb is still normal at 24.

Physiology Q35
A patient has a cardiac arrest at the dialysis unit. After appropriate cardiopulmonary resuscitation, return of spontaneous circulation is achieved. A blood gas is drawn at this time.

ABG 7.1 / pCO2 60 / pO2 120

Na 140, Chloride 100, Bicarb 8

What is the acid base disorder?

1. Pure high anion gap metabolic acidosis
2. Respiratory acidosis with compensatory metabolic alkalosis
3. Mixed gap – non gap metabolic acidosis
4. Pure high anion gap metabolic acidosis with compensatory respiratory alkalosis
5. Mixed high anion gap metabolic acidosis with respiratory acidosis

ANSWER: e. Pure high anion gap metabolic acidosis with mixed respiratory acidosis

1. High Anion Gap Metabolic Acidosis due to hypoperfusion and shock

2. Respiratory Acidosis due to hypoventilation

PH, low bicarb, anion gap 20 consistent with **metabolic acidosis**

Winter’s for Expected pCO2 = 1.5 * Bicarb + 8 +/- 2 = 20. Measured pCO2 = 60 = **Respiratory acidosis**

Delta Delta = (32-12) / (24-8) = 20/16 = 1.25

Delta Delta between 1 – 2 = **Pure Anion Gap Metabolic Acidosis**

Physiology Q36
The patient ingests a mystery substance at a party. She has altered mental status and diarrhea. In the ED, a blood gas is drawn.

ABG 7.3 / pCO2 15 / pO2 100

Na 140, Chloride 120, Bicarb 10

What is the acid base disorder?

1. Pure high anion gap metabolic acidosis
2. Mixed Non gap metabolic acidosis with respiratory alkalosis
3. Mixed Non gap metabolic acidosis with appropriate respiratory compensation
4. Mixed gap non gap metabolic acidosis with appropriate respiratory compensation
5. Mixed high anion gap metabolic acidosis with respiratory alkalosis

ANSWER: b. Mixed Non gap metabolic acidosis with respiratory alkalosis

1. Non anion gap metabolic acidosis – diarrhea

2. Respiratory Alkalosis – hyperventilating beyond the compensatory response

ABG 7.3/15, Anion gap 10, Bicarb 10 consistent with **primary non-gap metabolic acidosis**

Winter’s expected pCO2 = 1.5 * 10 + 8 +/- 2 = 23 +/- 2

Measured pCO2 = 15 which is lower than expected thus there is a **mixed** **respiratory alkalosis**

Physiology Q37
A patient is intubated for pancreatitis ARDS. She has multiorgan failure and shock and has received many amps of Bicarb. A blood gas is drawn.

ABG 6.9 / pCO2 80 / pO2 70

Na 140, Chloride 110, Bicarb 8

What is the acid base disorder?

1. High gap metabolic acidosis with mixed respiratory acidosis
2. Non gap metabolic acidosis with mixed respiratory acidosis
3. Mixed High gap and non-gap metabolic acidosis with respiratory acidosis
4. High gap metabolic acidosis with appropriate respiratory compensation

ANSWER: c. Mixed high gap and non-gap metabolic acidosis with respiratory acidosis

1 + 2. Mixed Gap and Non-Gap Metabolic Acidosis

3. Respiratory Acidosis

PH 6.9, Bicarb 8, Anion gap 22 consistent with **metabolic acidosis**

Delta Anion gap / Delta Bicarb = (22-12) / (24-8) = 10/16 = 0.625

Delta Delta < 1 indicates **mixed gap / non-gap metabolic acidosis**

Winter’s formula = 1.5 * 8 + 8 +/- 2 = 20 expected pCO2. Measured pCO2 = 80 consistent with **respiratory acidosis**

Physiology Q38
A young man overdoses on aspirin and presents to the ED.

ABG 7.48 / pCO2 14 / pO2 90

Na 126, K 2.2, Chloride 98, Bicarb 8

What is the acid base disorder?

1. Respiratory alkalosis with compensatory metabolic acidosis
2. Primary respiratory alkalosis with mixed gap and non gap metabolic acidosis
3. Metabolic alkalosis with compensatory respiratory acidosis
4. Metabolic acidosis with compensatory respiratory alkalosis

ANSWER: b. Primary respiratory alkalosis with mixed gap and non gap metabolic acidosis

1. Respiratory alkalosis – salicylate toxicity stimulates the respiratory drive center in the brain

2 + 3. Mixed Gap and Non-Gap metabolic acidosis- inhibition of Krebs cycle and lipid/glucose metabolism leads to increased metabolic acidosis. Salicylate itself is also an anion.

PH 7.48 with pCO2 14 indicates **primary respiratory alkalosis**

Bicarb 8, Anion Gap 20

Delta Anion gap / Delta Bicarb = (20-12)/(24-8) = 8 / 16 = 0.5

Delta Delta < 1 = **mixed gap and non gap metabolic acidosis**

Physiology Q39

A patient was using marijuana for pain control developed hyperemesis syndrome. The patient is in the ED with uncontrolled pain, agitation, and panic.

ABG pH 7.57 / pCO2 20 / pO2 80. Na 128, Chloride 78, Bicarb 23, Cr 1.8, BUN 60, Lactate 4. What is the acid base disorder?

1. Primary Respiratory alkalosis with compensatory metabolic acidosis
2. Mixed Respiratory alkalosis with metabolic acidosis
3. Mixed Respiratory alkalosis with mixed metabolic alkalosis
4. Mixed Respiratory alkalosis with mixed high anion gap metabolic acidosis and metabolic alkalosis
5. Mixed metabolic alkalosis with compensatory respiratory acidosis

ANSWER: d. Primary Respiratory alkalosis with mixed high anion gap metabolic acidosis and metabolic alkalosis

1. Respiratory Alkalosis: 7.57 / 20. Alkalosis from hyperventilation 2/2 panicking / pain

2. High anion gap metabolic acidosis: AG is 27, there must be a HAGMA

3. Metabolic alkalosis: expected bicarb would fall 2 for every 10 unit drop in pCO2.

PCO2 is 20 less than normal. Expected compensatory bicarb = 24 - 4 = bicarb 20.

Actual bicarb = 23. With the presence of the HAGMA, we would have expected the bicarb to be even lower. Thus there must be a metabolic alkalosis (from the vomiting and loss of gastric acid).

Delta Delta = 15 / 1 which further supports a **mixed high anion gap metabolic acidosis with a metabolic alkalosis**

Physiology Q40

Which of the following lung function parameters can NOT be measured by spirometry?

1. Vital capacity (VC)
2. Forced Vital Capacity (FVC)
3. Forced expired volume in one second (FEV1)
4. Total lung capacity (TLC)

ANSWER: d. TLC

Spirometry is part of pulmonary function testing. Specifically, it is a test that measures the ability to inhale and exhale air relative to time. Total lung capacity is not part of spirometry, but it is part of pulmonary function testing.

Physiology Q41

A 70 year old female smoker presents to pulmonology clinic. What do the PFTs suggest?

|  | **Result** | **Predicted** | **% Predicted** |
| --- | --- | --- | --- |
| **FEV1** | 2.5 | 3.6 | 69% |
| **FVC** | 4 | 4.5 | 89% |
| **FEV1/FVC** | 0.6 | 0.8 | 78% |
| **TLC** | 7 | 5.5 | 127% |
| **DLCO** | 20 | 25 | 80% |

1. Acute bronchitis
2. Emphysema
3. Pneumonia
4. Neuromuscular disease
5. Upper airway obstruction

ANSWER: b. Emphysema

The FEV1/FVC ratio < 0.7 suggests an obstructive airway disorder. The TLC is increased, and though the RV (Residual volume) is not given it may suggest hyperinflation. The DLCO is on the low end of normal; in severe emphysema you would expect the DLCO to be decreased.

Physiology Q42

A 55 year old female with rheumatoid arthritis and tobacco use disorder presents to pulmonology clinic. What do the PFTs suggest?

|  | **Result** | **Predicted** | **% Predicted** |
| --- | --- | --- | --- |
| **FEV1** | 2 | 3.2 | 63% |
| **FVC** | 2 | 3.5 | 57% |
| **FEV1/FVC** | 1 | 0.9 | 109% |
| **TLC** | 3 | 4.5 | 67% |
| **DLCO** | 18 | 25 | 72% |

1. Interstitial lung disease
2. Emphysema
3. Asthma
4. Bronchiectasis

ANSWER: a. Interstitial lung disease

The low TLC and normal FEV1/FVC is consistent with a restrictive disorder. Of the options, Interstitial Lung disease is the only option that would present as a restrictive disorder. Emphysema, asthma, and bronchiectasis classically present as obstructive airway disorders. A low DLCO is also consistent with Interstitial lung disease.

Physiology Q43

Which of the following would not produce a restrictive spirometry pattern on PFTs?

1. Bronchitis
2. Hepatic hydrothorax
3. Neuromuscular diseases
4. Morbid obesity

ANSWER: a. Bronchitis

Chronic Bronchitis will present as an obstructive airway disorder on PFTs. The other options all can cause extra-parenchymal restriction.

Physiology Q44

What does this flow volume loop suggest? (Obstructive disease, restrictive disease, fixed upper airway obstruction)


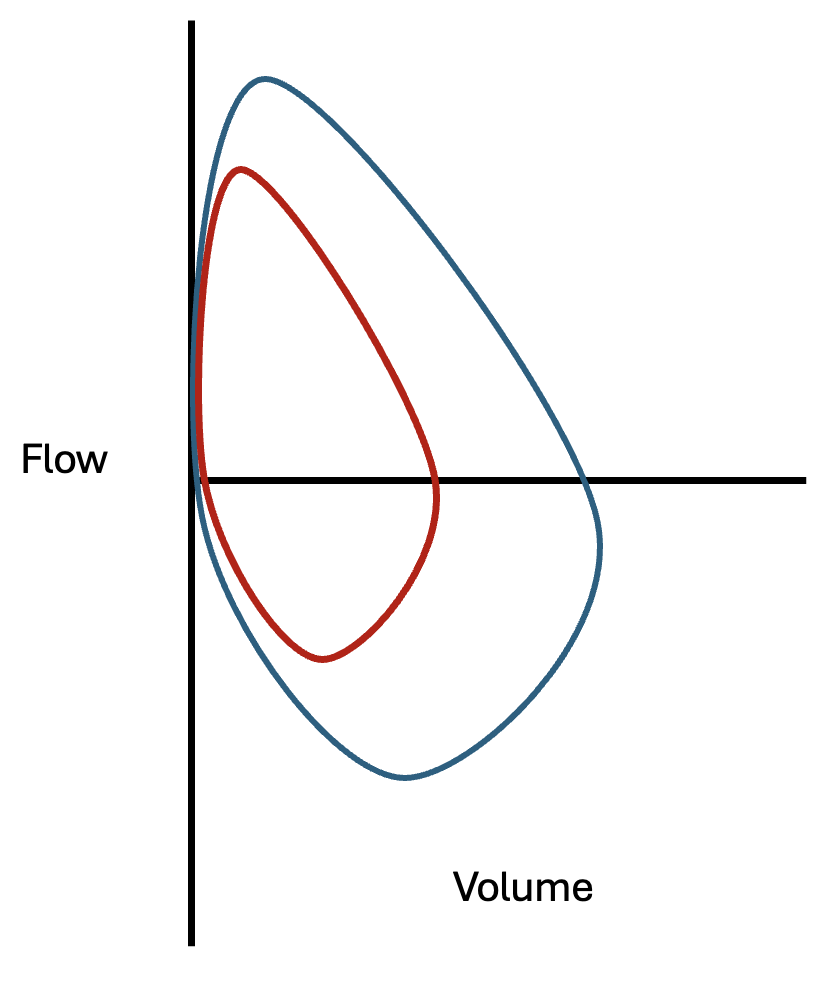


ANSWER: Restrictive lung disease

Physiology Q45

What does this flow volume loop suggest? (Obstructive disease, restrictive disease, fixed upper airway obstruction)


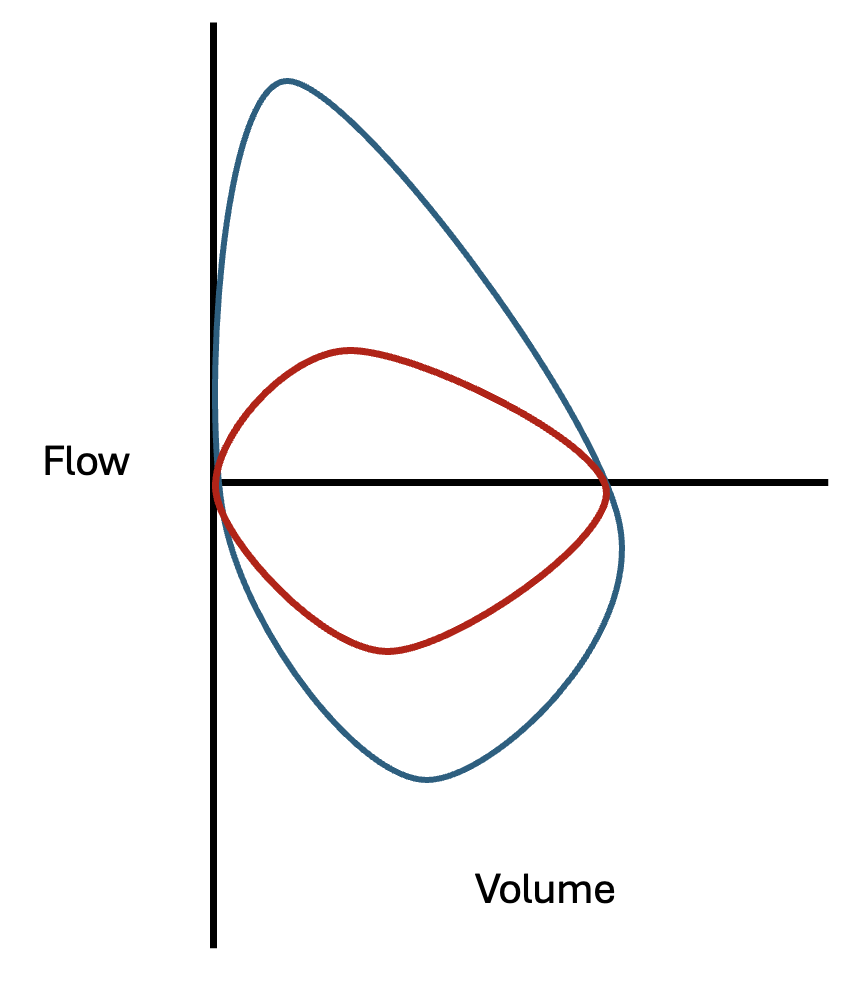


ANSWER: Fixed upper airway obstruction

Physiology Q46

What does this flow volume loop suggest? (Obstructive disease, restrictive disease, fixed upper airway obstruction)


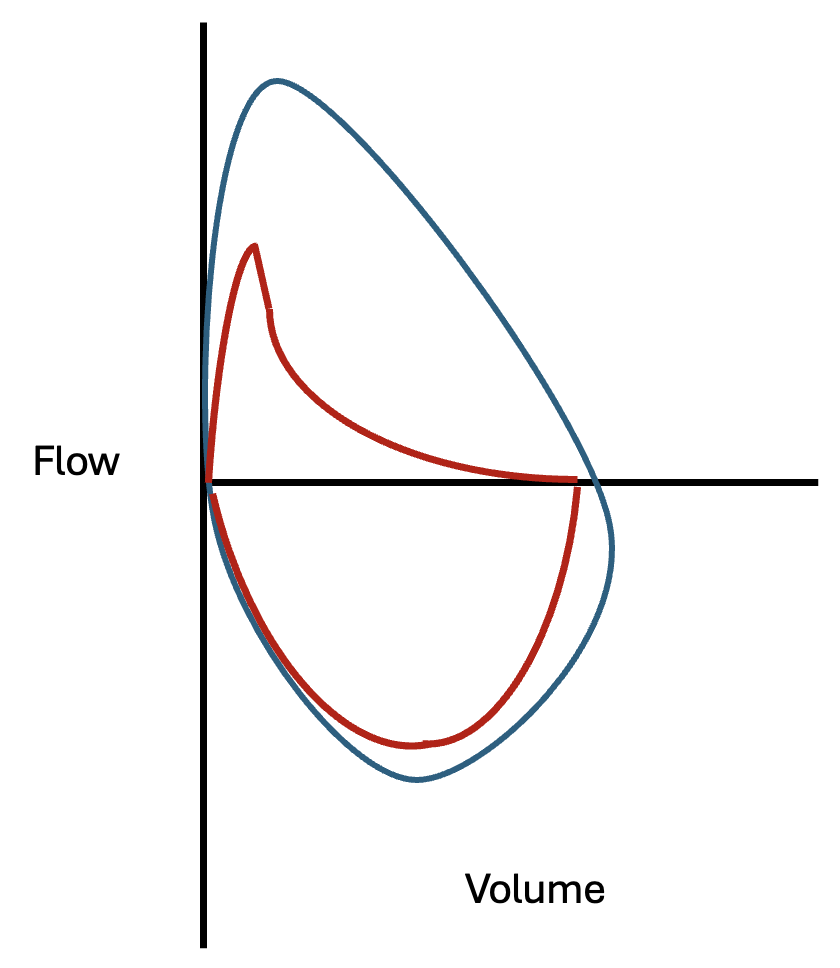


ANSWER: Obstructive lung disease

Physiology Q47

In pulmonary function testing, a certain medication is given as a bronchoprovocation challenge to aid in the diagnosis of asthma. What is this medication?

1. Albuterol
2. Fluticasone
3. Prednisone
4. Tiotropium
5. Methacholine

ANSWER: e. Methacholine

Physiology Q48

A patient develops cardiac arrest in the hospital. CPR is initiated and she is intubated. Capnography attached to the endotracheal tube demonstrates end tidal partial pressure of CO2 is 12 mmHg (EtCO2). What would you expect the capnography to show in the setting of poor quality compressions?

1. Gradual increase in EtCO2
2. Gradual decrease in EtCO2
3. Sudden increase in EtCO2
4. EtCO2 of > 30

ANSWER: b. Gradual decrease in EtCO2. The EtCO2 reflects pulmonary blood flow and capnography can be used as a noninvasive measure of cardiac output during CPR. With poor compressions the EtCO2 would drop.

Physiology Q49

Which of the following is associated with a pleural effusion with a triglyceride level >110 mg/dL?

1. Chylothorax
2. Pulmonary embolism
3. Nephrotic syndrome
4. Connective tissue disease

ANSWER: a. Chylothorax

Obstruction of the thoracic duct or other major lymphatics will lead to accumulation of lipid rich chyle in the pleural space. A triglyceride level >110 mg/dL is highly specific for a chylothorax.

Physiology Q50

A patient develops cardiac arrest in the hospital. CPR is initiated and she is intubated. Capnography attached to the endotracheal tube demonstrates end tidal partial pressure of CO2 is 12 mmHg (EtCO2). After 10 minutes of resuscitation, the EtCO2 rapidly increases to 37 mmHg. What explains this capnography finding?

1. Poor quality compressions
2. Pneumothorax due to compressions
3. Right mainstem intubation
4. Return of spontaneous circulation
5. Esophageal intubation

ANSWER: d. Return of spontaneous circulation. The sudden rise of EtCO2 is often the earliest indicator of ROSC, occurring even before a pulse is palpable.

**Pathophysiology**

Pathophysiology Q1

Which of the following mechanisms are least effective in the response to tuberculosis infection?

1. IL-12
2. Interferon—gamma
3. Tumor necrosis factor-alpha
4. CD20

ANSWER: d. CD20

Anti-CD20 treatment (rituximab) targets B lymphocytes. It is not a treatment for tuberculosis.

Pathophysiology Q2

Which of the following are NOT a reason for pulmonary tuberculosis’s preference for the upper lung lobes?

1. Lymphatic clearance is decreased in the upper lobes
2. Regional high oxygen tension in the upper lobes
3. Relative over-ventilation in the upper lobes
4. Decreased T cell response in the upper lobes

ANSWER: d. Decreased T cell response in the upper lobes

Pathophysiology Q3

Which of the following facts are NOT true about Bacillus of Calmette and Guerin (BCG) vaccination for tuberculosis?

1. The vaccination is a live attenuated strain of Mycobacterium bovis
2. It is not used in the USA because of low TB risk and interference with PPD screening
3. Overall, it is approximately 50% effective
4. People who received BCG 10 years ago and are tuberculin skin test positive likely are immune and not infected.

ANSWER: d. People who received BCG 10 years ago and are tuberculin skin test positive likely are immune and not infected.

BCG vaccinations can lead to false positives in addition to true positives. TB blood tests such as interferon gamma release assays will not result in false positives and are the preferred test in this population.

Pathophysiology Q4

In a patient with hypercarbic respiratory failure due to a COPD exacerbation, which of the following changes will help?

1. Increase tidal volume
2. Decrease respiratory rate
3. Increase FiO2
4. Increase positive end-expiratory pressure

ANSWER: a. Increase tidal volume

In a patient with hypercarbic respiratory failure due to a COPD exacerbation, they are retaining too much carbon dioxide and unable to ventilate. The treatment would be to increase the tidal volume and increase the respiratory rate.

Pathophysiology Q5

What type of immune hypersensitivity reaction does the purified protein derivative tuberculin skin test elicit?

1. Type I
2. Type II
3. Type III
4. Type IV

ANSWER: d. Type IV

This is a T-cell mediated reaction that is delayed, often with maximum reaction time between 48-72 hours.

Pathophysiology Q6

Which of the following is NOT a feature of a pleural effusion from rheumatoid arthritis?

1. Exudative
2. High glucose
3. Rheumatoid Factor positive
4. High protein

ANSWER: b. High glucose

Rheuamtoid pleuritis is often characterized by very low glucose, high protein, high LDH, and negative for malignant cells on cytology and negative for bacteria on gram stain.

Pathophysiology Q7

Which of the following is NOT a feature of malignant pleural effusion?

1. Exudative
2. Low glucose
3. High pH
4. High LDH

ANSWER: c. High pH

Malignant effusions are associated with low pH (less than 7.3), low glucose, high protein, high LDH, and often lymphocyte predominance the cell differential.

Pathophysiology Q8

In a patient with hypercarbic respiratory failure due to a COPD exacerbation treated with BIPAP, which of the following is most appropriate?

1. Increase the inspiratory pressure, keep the expiratory pressure the same
2. Increase the inspiratory pressure and the expiratory pressure
3. Increase the expiratory pressure and keep the inspiratory pressure the same
4. Change to continuous positive airway pressure.

ANSWER: a. Increase the inspiratory pressure, keep the expiratory pressure the same

A greater difference between the IPAP and EPAP will increase flow and tidal volume. This leads to more ventilation of the retained carbon dioxide.

Pathophysiology Q9

Which of the following is the studied indication for CODEX dosing (dexamethasone 20mg x 5 days followed by 10mg x 5 days) for treatment of COVID-19?

1. Hypoxia requiring supplemental oxygen
2. Moderate to severe ARDS, as defined by Berlin criteria
3. ICU admission
4. Requiring antibiotics

ANSWER: b. Moderate to severe ARDS, as defined by Berlin criteria

Pathophysiology Q10

Which of the following is not typically a part of the laboratory workup for a new diagnosis of community acquired pneumonia being admitted to the hospital?

1. SARS-CoV-2 rapid PCR test
2. Legionella urine antigen test
3. Blood or sputum cultures
4. CMV antigen or PCR test

ANSWER: d. CMV antigen or PCR test

Pathophysiology Q11

A patient presents with community acquired pneumonia and no prior hospital exposures, all of the following are common organisms except:

1. Streptococcus pneumoniae
2. Haemophilus influenzae
3. Pseudomonas aeruginosa
4. Staphylococcus aureus

ANSWER: c. Pseudomonas aeruginosa

Pathophysiology Q12

All of the following disease processes cause diffusion abnormalities on pulmonary function testing except for:

1. Pneumonia
2. Congestive heart failure
3. Cystic fibrosis
4. Interstitial lung disease
5. Asthma

ANSWER: e. Asthma

Pathophysiology Q13

Restrictive lung diseases have all the below characteristics on pulmonary function testing, except:

1. Normal residual volume
2. Low lung volumes
3. FEV1 to FVC ratio < 70%
4. No bronchodilator response

ANSWER: c. FEV1 to FVC ratio < 70%

This is characteristic of obstructive lung disease.

Pathophysiology Q14

What is the most common cause of Acute Respiratory Distress Syndrome (ARDS)?

1. Sepsis
2. Congestive heart failure
3. Blood product transfusion reaction
4. Drug toxicity
5. Injury from inhaled toxins or chemicals

ANSWER: a. Sepsis

In sepsis, the inflammatory response and high cytokine influx disrupts the alveolar-capillary barrier. This leads to fluid and inflammatory mileau entering the alveoli and can eventually cause ARDS.

Pathophysiology Q15

A patient is found to have acute respiratory distress syndrome (ARDS) and is intubated and mechanically ventilated. What should the target tidal volumes be? (in ml/kg of ideal body weight)

1. 6-8
2. 8-10
3. 10-12
4. 12-14

ANSWER: a. 6-8 ml/kg of ideal body weight

The ARDSNET study found that there was significant benefit to patients when using lung protective ventilation in ARDS. This includes a low tidal volume ventilation to reduce volutrauma and barotrauma in the alveoli.

Pathophysiology Q16

The Berlin criteria for Acute Respiratory Distress Syndrome include all of the following except:

1. Acute onset, less than 1 week
2. Bilateral opacity on chest XR or CT scan
3. Ratio of the arterial partial oxygen tension, or PaO2, to the fraction of inspired oxygen, or FiO2 for severe ARDS is < 100
4. SpO2 less than 88% on room air

ANSWER: d. SpO2 less than 88% on room air

The Berline criteria uses a ratio of PaO2 to FiO2 to define and characterize ARDS, rather than measuring SpO2.

Pathophysiology Q17

All of the following are subtypes of Non-Small Cell Lung Cancer except:

1. Squamous
2. Endothelial
3. Adenocarcinoma
4. Large cell neuroendocrine carcinoma

ANSWER: b. Endothelial

Non-small cell lung cancer has three subtypes. Adenocarcinoma is the most common, followed by squamous cell and large cell.

Pathophysiology Q18

Which of the following symptoms is not typical for an Apical Pancoast lung cancer causing Horner’s Syndrome?

1. Lateral gaze palsy
2. Drooping upper eyelid
3. Constricted pupil
4. Anhidrosis

ANSWER: a. Lateral gaze palsy

Horner syndrome is caused by a tumor disrupting the sympathetic nerves supplying the head and neck. The classic triad is ptosis, anhidrosis, and miosis. Lateral gaze palsy is caused by defect to cranial nerve VI.

Pathophysiology Q19

The following are paraneoplastic syndromes commonly associated with Small cell carcinoma except:

1. Syndrome of inappropriate ADH
2. Eaton Lambert syndrome
3. Cushings syndrome
4. Nephrotic syndrome

ANSWER: d. Nephrotic syndrome

Pathophysiology Q20

Which of the following are molecular targets for lung cancer treatment?

1. BCR-ABL
2. TNF-alpha
3. PD-L1
4. CD20

ANSWER: c. PD-L1

Programmed Death Ligand 1 is a molecular target for lung cancer. It is associated with worse survival. Patients who test positive for PD-L1 are treated with immunotherapy, such as pembrolizumab.

Pathophysiology Q21

All of the following are World Health Organization defined groups for pulmonary hypertension except:

1. Pulmonary arterial hypertension
2. Pulmonary hypertension due to left heart disease
3. Pulmonary hypertension due to liver disease
4. Pulmonary hypertension due to lung disease
5. Pulmonary hypertension due to chronic thromboembolic disease

ANSWER: c. Pulmonary hypertension due to liver disease

Group 5 is a miscellaneous group of diseases that cause pulmonary hypertension. To name a few: sarcoidosis, hematologic disorders, cancer, glycogen storage metabolic disorders.

Pathophysiology Q22

A patient without prior history of sarcoidosis presents with a high suspicion for pulmonary sarcoidosis that requires definitive diagnostic testing. Which of the following is the best approach?

1. Biopsy an enlarged cutaneous lesion thought to be also due to sarcoid
2. Transbronchial biopsy of a hilar lymph node
3. Transcutaneous biopsy of parenchymal lung tissue
4. Measurement of serum angiotensin converting enzyme

ANSWER: a. Biopsy an enlarged cutaneous lesion thought to be also due to sarcoid

For tissue diagnosis of sarcoidosis, guidelines recommend targeting the lowest risk and most accessible lesion. A biopsy of a cutaneous lesion would be appropriate before targeting a pulmonary or hilar lesion.

Pathophysiology Q23

Allergic bronchopulmonary aspergillosis is characterized by all of the following except:

1. Eosinophilic pneumonia
2. Mucoid impaction of the bronchi
3. Bronchocentric granulomatosis
4. Restrictive lung disease on pulmonary function testing

ANSWER: d. Restrictive lung disease on pulmonary function testing

ABPA causes bronchoconstriction and an obstructive pattern on PFTs.

Pathophysiology Q24

Allergic bronchopulmonary aspergillosis usually occurs in patients with:

1. Asthma
2. COPD
3. Sarcoidosis
4. Tuberculosis

ANSWER: a. Asthma

Pathophysiology Q25

Patients with cystic fibrosis are commonly colonized with bacteria in their respiratory system. Which of the following is NOT a common organism in these patients?

1. Staphylococcus aureus
2. Pseudomonas aeruginosa
3. Stenotrophomonas maltophila
4. Burkholderia cepacia complex
5. Tuberculosis mycobacterium

ANSWER: e. Tuberculosis mycobacterium

Pathophysiology Q26

All of the following are characteristics of cystic fibrosis pulmonary disease, except:

1. Neutrophilic release of elastase that overwhelms anti-proteases which increases tissue destruction
2. Bronchiectasis
3. Respiratory alkalosis
4. Impaired mucociliary clearance
5. Airway hyperreactivity responsive to bronchodilator therapy

ANSWER: c. Respiratory alkalosis

Cystic fibrosis patients often have a respiratory acidosis and metabolic alkalosis.

Pathophysiology Q27

All of the following are associated conditions with cystic fibrosis, except:

1. Obstructive sleep apnea
2. Tracheomalacia
3. Allergic bronchopulmonary aspergillosis
4. Pulmonary hypertension
5. Small cell lung cancer

ANSWER: e. Small cell lung cancer

Pathophysiology Q28

A 20 year old patient with cystic fibrosis presents to your outpatient clinic for vaccinations. Which of the following vaccinations are appropriate for this patient?

1. Seasonal influenza vaccine
2. Pneumococcal vaccine
3. COVID-19 vaccine
4. HPV vaccine
5. All of the above

ANSWER: e. All of the above

Pathophysiology Q29

The Global Initiative for Chronic Obstructive Lung Disease (GOLD) guidelines classify COPD based on all of the following except:

1. Exacerbation history / hospitalizations
2. Modified Medical Research Council dyspnea questionaire
3. COPD assessment test
4. DLCO

ANSWER: d. DLCO

Pathophysiology Q30

Which of the following are signs of hypercapnia?

1. Asterixis
2. Confusion
3. Somnolence
4. Increased respiratory rate
5. All of the above

ANSWER: e. All of the above

Pathophysiology Q31

A patient has a ground level fall and a resulting radial fracture. An arterial blood gas checked after she presented to the ED was pH 7.50, pO2 140, pCO2 26, HCO3 21. Which of the following etiologies is consistent with this blood gas in this clinical setting?

1. Tachypnea from pain
2. Submassive Pulmonary embolism
3. Asthma exacerbation
4. Pulmonary edema

ANSWER: a. Tachypnea from pain

Tachypnea leads to hyperventilation and decreased carbon dioxide. This leads to a respiratory alkalosis. If enough time has passed, the kidneys will create a compensatory metabolic acidosis.

Pathophysiology Q32

The most common mutation for Cystic Fibrosis is the CF transmembrane conductance regulator (CFTR) gene mutation delta-F508 on chromosome:

1. 3
2. 5
3. 7
4. 9

ANSWER: c. 7

Pathophysiology Q33

An abnormal enlargement and decreased function of the right ventricle as a consequence of increased pulmonary hypertension is termed:

1. Pulmonic regurgitation
2. Cor Pulmonale
3. Ventricular septal defect
4. Takutsubo cardiomyopathy

ANSWER: b. Cor Pulmonale

Pathophysiology Q34

WHO Group 2 pulmonary hypertension is due to this etiology:

1. Lung disease
2. Left heart disease
3. Chronic thromboembolic lung disease
4. Pulmonary arterial hypertension

ANSWER: b. Left heart disease

Pathophysiology Q35

WHO Group 4 pulmonary hypertension is due to this etiology:

1. Lung disease
2. Left heart disease
3. Chronic thromboembolic lung disease
4. Pulmonary arterial hypertension

ANSWER: c. Chronic thromboembolic lung disease

Pathophysiology Q36

WHO Group 3 pulmonary hypertension is due to this etiology:

1. Lung disease
2. Left heart disease
3. Chronic thromboembolic lung disease
4. Pulmonary arterial hypertension

ANSWER: a. Lung disease

Pathophysiology Q37

A 80 year old male with COPD and alcohol use disorder complains of 6 weeks of progressively worsening dyspnea and productive cough. Chest XR demonstrates a right lower lobe cavitary lesion with an air fluid level in the right lower lobe. Which of the following is the best treatment to initiate?

1. Rifampin, Isoniazid, Pyrazinamide, Ethambutol
2. IV ampicillin-sulbactam
3. IV metronidazole
4. IV Ceftaroline
5. IV Vancomycin

ANSWER: b. IV ampicillin-sulbactam

The clinical picture is consistent with an aspiration pneumonia and a pulmonary abscess. Lung abscesses require prolonged treatment with IV antibiotics. In addition to coverage of usual community acquired organisms, anaerobic coverage is also needed.

Pathophysiology Q38

Which of the following arterial blood gases is most consistent with a patient with chronic COPD, presenting with baseline dyspnea on exertion and vitals T 37.1, BP 140/95, HR 88, respirations 20/min.

1. PH 7.3, bicarbonate 33, anion gap 9
2. PH 7.3, bicarbonate 24, anion gap 12
3. PH 7.3, bicarbonate 18, anion gap 16
4. PH 7.46, bicarbonate 24, anion gap 12
5. PH 7.46, bicarbonate 33, anion gap 9

ANSWER: a. PH 7.3, bicarbonate 33, anion gap 9

With chronic COPD, we would expect that the carbon dioxide retention would lead to a respiratory acidosis. Metabolic compensation would lead to increased serum bicarbonate. We would not expect an anion gap unless there was another mixed acid-base process.

Pathophysiology Q39

A 32 year old male presents with dyspnea and decreased functional status that is progressively worsening over months. He does not smoke cigarettes, he works a desk job, has no previous lung disease. Chest XR imaging demonstrates basilar emphysematous changes. Spirometry shows FEV1 58%, FVC 70%, FEV1/FVC 62% and postbronchodilator FEV1 58%. Labs are unremarkable except for mild transaminitis. What is the best next test?

1. Alpha-1 antitrypsin testing
2. Repeat pulmonary function testing in 3 months
3. Transthoracic echocardiogram
4. Empiric initiation of asthma treatment
5. High resolution CT of the chest

ANSWER: a. alpha-1 antitrypsin testing. Basilar emphysema and liver disease in a young man with no other risk factors for lung disease should raise your suspicion for this disease. His spirometry is obstructive without a bronchodilator response, not consistent with asthma.

Pathophysiology Q40

All of the following are findings consistent with obesity hypoventilation syndrome and sleep apnea except:

1. Right heart dilation
2. Bicarbonate elevation
3. Hypercarbia
4. Anemia
5. FEV1/FVC ratio of 88%

ANSWER: d. anemia. The effects of OHS and OSA often lead to erythrocytosis.

Pathophysiology Q41

Interstitial lung disease associated with polymyositis or dermatomyositis is heavily correlated with the presence of which of the following antibodies?

1. Anti-Jo 1
2. Anti-Mitochondrial
3. Anti-double stranded DNA
4. Anti-IgE
5. Anti-SSA

ANSWER: a. Anti-Jo 1

Pathophysiology Q42

Which of the following autoimmune disorders is the most common cause of pulmonary-renal syndrome?

1. Eosinophilic granulomatosis with polyangiitis (Churg Strauss)
2. Microscopic polyangiitis
3. Rheumatoid arthritis
4. SLE
5. Takayasu arteritis

ANSWER: b. Microscopic polyangiitis

It is often characterized by diffuse alveolar hemorrhage and glomerulonephritis.

Pathophysiology Q43

A patient is being treated with high dose prednisone for Giant cell arteritis and presents with cough. Chest CT demonstrates ground glass opacities and cavitary nodules. Galactomannan positive in the serum. What is the etiology?

1. Aspergillosis
2. Histoplasmosis
3. Mycobacterial tuberculosis
4. Mycobacterium avium complex
5. Small cell lung cancer

ANSWER: a. Aspergillosis

Steroid use increases the risk of invasive aspergillosis. The imaging findings are consistent with it and the galactomannan study has relatively high sensitivity and specificity for aspergillosis.

Pathophysiology Q44

Honeycombing is a radiographic finding of clustered cystic air spaces (usually 3-10 mm in diameter) that are usually basilar, peripheral, and subpleural. This finding is most commonly associated with:

1. Small cell lung cancer
2. Alpha-1 antitrypsin deficiency
3. Usual interstitial pneumonia (UIP)
4. Asthma
5. Pulmonary embolism

ANSWER: c. Usual interstitial pneumonia (UIP)

UIP is a radiologic and histologic pattern and is a type of interstitial lung disease. Common etiologies include connective tissue disease and vasculitis.

Pathophysiology Q45

Eosinophilic granulomatosis with polyangiitis is an ANCA vasculitis characterized by multiorgan involvement. Asthma is a cardinal feature, which of the following is the next most common clinical feature?

1. Gastritis
2. Renal artery stenosis
3. Rhinosinusitis
4. Monoarticular arthritis
5. Aortic root dilation

ANSWER: c. Rhinosinusitis

Pathophysiology Q46

In a patient with a heparin induced thrombocytopenia and new diagnosis of pulmonary embolism, which of the following is the best management?

1. Warfarin
2. Low molecular weight heparin
3. Argatroban
4. Inferior vena cava filter placement

ANSWER: c. Argatroban

Heparin derived products must be avoided. Warfarin is avoided because protein C and S depletion can worsen thrombotic events. An IVC filter is not appropriate. Argatroban is the best choice.

Pathophysiology Q47

A 30 year old female has a pulmonary embolus diagnosed in the setting of a COVID infection. Which of the following is the most appropriate therapy?

1. Apixaban for 1 month
2. Rivaroxaban for 3 months
3. Apixaban for 1 year
4. Heparin for 6 months
5. Lovenox for 1 year

ANSWER: b. Rivaroxaban for 3 months

The patient has a provoked pulmonary embolism and thus will require anticoagulation for at least 3 months. A DOAC is an appropriate choice.

Pathophysiology Q48

A 20 year old patient is diagnosed with narcolepsy due to severe daytime somnolence, sleep paralysis, disrupted nocturnal sleep. What is the best next step in management?

1. Modafinil
2. CPAP
3. Improve sleep hygiene
4. Methylphenidate

ANSWER: a. Modafinil is the first line treatment and is preferred over amphetamines or methylphenidate.

Pathophysiology Q49

Loffler syndrome is characterized by all of the following except:

1. Helminth infection with transpulmonary migration
2. Peripheral blood eosinophilia
3. Hemoptysis
4. Transient pulmonary radiographic opacities

ANSWER: c. Hemoptysis

In an immunocompetent patient, Loffler syndrome often has a mild presentation. Hemoptysis is rare.

Pathophysiology Q50

The following are contra-indications to thrombolytic therapy for patients with pulmonary embolism, except:

1. Suspected aortic dissection
2. Malignant intracranial neoplasm
3. Ischemic stroke within the last three months
4. Hemodynamic instability

ANSWER: d. Hemodynamic instability

Thrombolytic therapy is indicated in patients with a massive pulmonary embolism, defined as a pulmonary embolism with systemic hypotension, shock, or a drop in systolic blood pressure by 40mmHg.

**“Modifier” Cards**

Modifier Q1

Which of the following treatments for Tuberculosis cause a reversible and dose dependent decrease in your green-red visual discrimination?

1. Ethambutol
2. Pyrazinamide
3. Rifampin
4. Isoniazid

ANSWER: a. Ethambutol

Modifier Q2

Which of the following is the preferred treatment for Mycobacterium Avium Complex in HIV patients?

1. Rifampin and Isoniazid for 9 months
2. Isoniazid, Rifampin, Pyrazinamide, and Ethambutol for 2 months then Isoniazid and Rifampin for 4 months
3. Clarithromycin and ethambutol for 12 months
4. Ampicillin-Sulbactam and Clarithromycin for 6 weeks with followup CT chest imaging

ANSWER: c. Clarithromycin and ethambutol for 12 months

MAC treatment requires a macrolide in combination with ethambutol or rifampin. The treatment course is very long - guidelines recommend 12 months of treatment after negative sputum culture conversion.

Modifier Q3

In which stage of airway and respiratory development do true alveoli form?

1. Embryonic
2. Pseudoglandular (approximate gestational week 5 – 18)
3. Canalicular (approximate gestational week 16 – 27)
4. Saccular (approximate gestational week 24 – birth)

ANSWER: d. Saccular (approximate gestational week 24 – birth)

Modifier Q4

In which stage of airway and respiratory development does surfactant first start being produced?

1. Embryonic
2. Pseudoglandular (approximate gestational week 5 – 18)
3. Canalicular (approximate gestational week 16 – 27)
4. Saccular (approximate gestational week 24 – birth)

ANSWER: c. Canalicular (approximate gestational week 16 – 27)

Modifier Q5

The CURB-65 score is used to estimate mortality of community acquired pneumonia to help guide decision-making for inpatient admission vs outpatient management. Which of the following is not a component of this score?

1. Age
2. Altered mental status
3. BUN
4. Blood pressure
5. Respiratory acidosis

ANSWER: e. Respiratory acidosis

The CURB-65 score has 5 components: Confusion, BUN > 19 mg/dL, Respiratory rate >30, Blood pressure < 90/60, and Age > 65.

Modifier Q6

A patient in the post-anesthesia care unit is found to be cyanotic and apneic, with a pulse and normotension. After starting bag valve mask ventilation, supplemental oxygen, and preparing for intubation, which of the following actions should be taken?

1. Serum and urine drug screen for opiates
2. Give Narcan empirically
3. Start a pressor such as norepinephrine
4. Give antibiotics empirically
5. Order a bedside echocardiogram

ANSWER: b. Give Narcan empirically

Modifier Q7

A patient with cirrhosis (not a transplant candidate) and persistent hepatic hydrothorax presents with shortness of breath. Which of the following is the most appropriate management of the hydrothorax?

1. Chest tube placement
2. Serial thoracentesis based on symptoms
3. Pleurodesis
4. Long term prophylactic antibiotics

ANSWER: b. Serial thoracenteses based on symptoms

Serial thoracenteses is the most appropriate management. Chest tube placement leads to a high risk of infection. Pleurodesis is relatively ineffective for treatment of hepatic hydrothorax. Long term antibiotics are not indicated unless he has history of spontaneous bacterial peritonitis.

Modifier Q8

In Guillain-Barre syndrome, which of the following methods is the best monitoring to determine if a patient requires ventilatory support?

1. PaO2
2. SpO2
3. Forced Vital Capacity
4. Forced expiratory volume in 1 second
5. Total lung capacity

ANSWER: c. Forced Vital Capacity

FVC is the gold standard for measuring respiratory muscle strength in this setting. An FVC < 20 ml/kg should prompt critical care evaluation and consideration of mechanical support.

Modifier Q9

Ashbaugh and Petty first named the syndrome of respiratory failure, “Acute Respiratory Distress Syndrome”, in 1967 in *Lancet*. In what setting was ARDS most notably described and studied in the 1960s and 70s?

1. Lung transplant surgery
2. Study of congestive heart failure
3. Wartime lung injury from surgery and blood transfusions
4. Inhalation injury from fires, studied in firefighters and victims

ANSWER: c. Wartime lung injury from surgery and blood transfusions

Modifier Q10

All of the following ventilator strategies are utilized for management of Acute Respiratory Distress Syndrome except:

1. Permissive hypercapnea
2. High tidal volume goal of 10-12 cc/kg ideal body weight
3. Minimize volume overload with diuresis
4. Use of sedatives and paralytics to reduce work of breathing and improve compliance with the ventilator

ANSWER: b. High tidal volume goal of 10-12 cc/kg ideal body weight

Guidelines recommend a low tidal volume, lung protective ventilatory strategy to avoid volutrauma and barotrauma. The tidal volume is usually 6-8 cc/kg ideal body weight.

Modifier Q11

Which of the following is the best explanation for why slowing down the respiratory rate of a patient with severe asthma exacerbation can improve their respiratory status?

1. Prolong expiratory time and improve hyperinflation
2. Increases the patient’s total lung capacity
3. Improves gas exchange
4. Decreases the patient’s FEV1

ANSWER: a. Prolong expiratory time and improve hyperinflation

Severe bronchospasm, mcuous production, and airway constriction leads to inability to ventilate in a severe asthma exacerbation. Dynamic hyperinflation leads to barotrauma and increased dead space. By slowing down the respiratory rate, there is more time for the expiratory phase and thus decrease the hyperinflation.

Modifier Q12

The US preventative services task force (USPSTF) would recommend a low dose CT scan for lung cancer screening in your 60 year old patient with:

1. 15 pack-year smoking history and current smoker
2. 30 pack-year smoking history who quit smoking 20 years ago
3. 20 pack-year smoking history, current smoker, advanced pancreatic cancer with 6 month life expectancy
4. 20 pack-year smoking history who quit smoking 5 years ago

ANSWER: d. 20 pack-year smoking history who quit smoking 5 years ago

Modifier Q13

The Pulmonary Embolism Severity Index (PESI) is a scoring system to guide prognosis after pulmonary embolism. All of the following are components of this system except:

1. History of cancer
2. Heart failure
3. Blood pressure
4. Current use of anticoagulation

ANSWER: e. Current use of anticoagulation

The PESI scoring system includes: age, sex, history of cancer, heart failure, chronic lung disease, vital signs, and altered mental status. This scoring system helps classify patients already diagnosed with a pulmonary embolism and predict risk of mortality and morbidity.

Modifier Q14

Which of the following is NOT a criteria for acute respiratory distress syndrome (ARDS)?

1. Onset of respiratory symptoms within 1 week of insult
2. Bilateral lung opacities
3. Signs of cardiac failure and fluid overload
4. PaO2/FiO2 ratio < 300

ANSWER: c. signs of cardiac failure and fluid overload

A diagnosis of ARDS necessitates that you rule out a cardiogenic cause for the lung infiltrates.

Modifier Q15

A patient with newly diagnosed COVID-19 has a 2L oxygen requirement. Their D-dimer is normal. They have no history of acute ischemic stroke, myocardial infarction, deep vein thrombosis or pulmonary embolism. Their labs are otherwise unremarkable. What is the best approach to anticoagulation?

1. Prophylactic anticoagulation with low molecular weight heparin
2. Therapeutic anticoagulation with unfractionated heparin
3. Therapeutic anticoagulation with low molecular weight heparin
4. Therapeutic anticoagulation with apixaban or rivaroxaban

ANSWER: a. Prophylactic anticoagulation with low molecular weight heparin

Though COVID increases the risk of thromboembolic events, in a patient with no indication for therapeutic anticoagulation, guidelines recommend prophylactic dose anticoagulation.

Modifier Q16

A patient with allergic bronchopulmonary aspergillosis is unable to taper off of prednisone despite treatment with antifungal drugs and management of asthma. What is the next best approach?

1. Start anti- IL 5 agent such as mepolizumab
2. Start anti- TNF alpha agent such as infliximab
3. Change to dexamethasone
4. Start empiric antibiotics

ANSWER: a. Start anti- IL 5 agent such as mepolizumab

In patients with ABPA complicated by asthma refractory to steroids and antifungal drugs, treatment with anti- IL5 / IL5Ra monoclonal drugs (mepolizumab, benralizumab) demonstrated decreased exacerbation and improved pulmonary function.

Modifier Q17

A patient has persistent cough after a viral respiratory illness. She complains of a nonpurulent cough for 2 weeks that initially had productive yellow sputum and now has thin white mucous. There is no fever, chills, vital sign abnormalities and the lungs are clear. What is the next best step in management?

1. Chest CT
2. Moxifloxacin
3. Sputum cultures
4. Albuterol nebulizer

ANSWER: e. Albuterol nebulizer. Only symptomatic management is needed in this patient with a recent viral illness. There are no signs of a bacterial superinfection. Antibiotics, cultures, and chest CT are not indicated at this time.

Modifier Q18

A patient is diagnosed with invasive aspergillosis characterized by several cavitary lung nodules and hemoptysis in the setting of immunosuppression. In addition to reducing immunosuppression, what is the next best therapy?

1. Rifampin
2. Anidulafungin
3. Voriconazole
4. Vancomycin

ANSWER: d. Voriconazole

The Infectious Diseases Society of America recommends Voriconazole as initial therapy for invasive aspergillosis, with the addition of an echinocandin in select cases.

Modifier Q19

Patients with cystic fibrosis should undergo genotyping to guide treatment for:

1. Antibiotic therapy resistances
2. CF transmembrane conductance regulator modulators
3. Anti-IL 5 therapy
4. Anti-Ig E therapy

ANSWER: b. CF transmembrane conductance regulator modulators

All patients with CF should undergo genotyping. CFTR modulators have shown to improve FEV1, quality of life, and to reduce the number of acute exacerbations.

Modifier Q20

A patient presents to your office for followup of COPD. Her resting PaO2 is 55mmHg and oxygen saturation is 88%. Her FEV1/FVC is 55%. Which of the following treatments can prolong survival?

1. Long-term oxygen therapy
2. Inhaled long acting beta agonist combined with corticosteroid
3. Inhaled long acting muscarinic antagonist
4. Inhaled short acting beta agonist
5. Nightly CPAP

ANSWER: a. Long-term oxygen therapy

In patients with COPD, long term oxygen therapy is indicated for resting PaO2 less than or equal to 55 mmHg or an SpO2 less than or equal to 88%. It has been shown to reduce mortality in this patient population.

Modifier Q21

Most COPD patients target pulse ox of at least 88%. Which of the following comorbid conditions will raise your targeted pulse ox goal to 90%?

1. Aortic stenosis
2. Sarcoidosis
3. Cor pulmonale
4. Asthma
5. Chronic kidney disease

ANSWER: c. Cor pulmonale

In patients with COPD, long term oxygen therapy is indicated for resting PaO2 less than or equal to 55 mmHg or an SpO2 less than or equal to 88%. There is evidence for a goal of PaO2 less than or equal to 59 mmHg or SpO2 of 89% for cor pulmonale, right heart failure, or erythrocytosis.

Modifier Q22

A patient with COPD presents with an exacerbation. He is started on appropriate medical therapy and nebulizers. 3 hours later, his Arterial blood gas demonstrates pH 7.22, PaCO2 82, PaO2 72. He is lethargic but awake and oriented. Which of the following is the best next step in management?

1. Increase oxygen supplementation
2. Intubation and mechanical ventilation
3. Continuous albuterol
4. Bilevel positive airway pressure ventilation

ANSWER: d. Bilevel positive airway pressure ventilation

The patient has acute hypercarbic respiratory failure due to a COPD exacerbation. BIPAP will help the patient ventilate and blow of carbon dioxide. If the patient were unable to protect their airway or too somnolent, then intubation and mechanical ventilation would be the most appropriate next step.

Modifier Q23

A patient with history of hypertension presents with progressive dyspnea and is found to have diastolic heart failure. Echocardiogram shows a left ventricular ejection fraction of 55%. Which of the following medications is indicated for this condition and reduced the combined risk of cardiovascular death and hospitalization?

1. Metoprolol
2. Valsartan-sacubitril
3. Empagliflozin
4. Apixaban

ANSWER: c. Empagliflozin

The EMPEROR-Preserved trial demonstrated the benefit of Empagliflozin in heart failure with preserved ejection fraction.

Modifier Q24

A 60 year old man with 40 pack year smoking history presents to clinic for an incidentally discovered 2.4cm spiculated nodule in the periphery of the right lung. He has no B symptoms and vitals are normal. No family history of cancer. What is the best management of the nodule?

1. Sputum culture and cytology
2. Surgical wedge resection
3. Bronchoscopy with transbronchial biopsy
4. Repeat CT scan in 6 months
5. Smoking cessation

ANSWER: b. surgical wedge resection

This patient has high risk for lung cancer and a suspicious nodule. The peripheral location and patient factors make a surgical wedge resection the most appropriate management for the nodule. Smoking cessation is important, but it does not address management of the nodule.

Modifier Q25

A 75 year old patient presents to the ED with shortness of breath. He was discharged to a nursing home 3 days ago after being treated for pneumonia and completed his antibiotics. Presenting vitals T100.4 HR 110 BP 110/80 SpO2 89% on room air. Chest XR shows a resolving density in the right lung base. Lungs are clear to your auscultation. What is the best next step?

1. V/Q scan
2. CT pulmonary angiography
3. Antibiotics
4. High resolution CT chest

ANSWER: b. CT pulmonary angiography.

The patient’s Well’s score was > 4, raising your pre-test probability of pulmonary embolism. V/Q scan is not appropriate because of the existing lung disease, which would confound the results.

Modifier Q26

In eosinophilic granulomatosis with polyangiitis (EGPA), the prodromal phase is characterized by asthma and atopic diseases, the eosinophilic phase is notable for extravascular eosinophilia to organs such as the lungs and GI tract. The vasculitis phase is characterized by all of the following except:

1. Myocarditis
2. ANCA associated glomerulonephritis
3. Palpable purpura
4. Aortic dissection

ANSWER: d. aortic dissection.

EGPA is characteristically a medium and small vessel vasculitis. Aortic involvement is seen in large vessel vasculitis such as Takayasu or Giant cell arteritis.

Modifier Q27

A 32 year old African American male presents with months of progressive fatigue, nonproductive cough, fevers, night sweats. Labs are unremarkable. He has erythema nodosum on physical exam and lungs are clear bilaterally. Chest imaging demonstrates bilateral hilar lymphadenopathy and no parenchymal consolidations. What is the best next step?

1. Biopsy of the erythema nodosum skin lesion
2. Endobronchial biopsy of a hilar lymph node
3. Surgical resection of a complete lymph node
4. High resolution CT imaging of the chest

ANSWER: b. Endobronchial biopsy of a hilar lymph node.

The patient likely has sarcoidosis and tissue biopsy of the most amenable target is needed to confirm the diagnosis (other etiologies on the differential include vasculitis, lymphoma, infection). The erythema nodosum lesions do not demonstrate noncaseating granulomas and are not an appropriate biopsy target.

Modifier Q28

A 50 year old male in the surgical ICU is intubated after an extensive bowel surgery and is post operative day 1. She is on IV antibiotics, fentanyl, midazolam and you are called to bedside because she is hypertensive to 190/120, agitated, and there is ventilator dyssynchrony. Other vitals are T37 HR 110 RR 35 and SpO2 98%. Arterial blood gas: pH 7.50, PaO2 110, PaCO2 25. The labs are all unchanged from prior. Chest imaging is unremarkable. What is the best next step?

1. Start a titratable anti-hypertensive agent
2. Decrease the tidal volume
3. Increase fentanyl dose
4. Start a paralytic to improve vent synchrony

ANSWER: c. Increase fentanyl dose.

The patient has a respiratory alkalosis and has signs of uncontrolled pain. He is unable to express it due to being intubated and it is interpreted as hypertension, agitation, ventilator dyssynchrony. The other objective data is reassuring that there are no other organic causes. The best next step is to increase the fentanyl and observe if that improves the signs and symptoms.

Modifier Q29

A patient with COPD on long term oxygen therapy of 1L by nasal cannula plans on flying from Virginia to New York to visit family. She needs 1L of oxygen at rest and maintains SpO2 of 96%. What should be done for this patient for her flight?

1. Pulmonary function testing
2. Pharmacologic stress test
3. Increase supplemental oxygen by 2L/min for the flight
4. Six minute walk test
5. No changes are needed

ANSWER: c. Increase supplemental oxygen by 2L for the flight.

The cabin FiO2 is approximately 15% compared to 21% at sea level. For patients with COPD on oxygen, their baseline oxygen flow rate should be increased by 1-2 L/min. In a different scenario, if the patient had been at 96% at rest with no baseline oxygen needs, she would not have required any supplemental oxygen or further testing before the flight.

Modifier Q30

A 55 year old man is admitted with pneumonia. Chest imaging was notable for a moderate to large right sided pleural effusion. A diagnostic thoracentesis was performed and notable for pH 7.15, glucose 36, and gram stain with many neutrophils and bacteria. What is the next best step?

1. Repeat thoracentesis tomorrow
2. Repeat chest XR tomorrow to guide whether to perform thoracentesis
3. Chest tube placement immediately
4. Thoracic surgery consult for video assisted thorascopic surgery and pleurodesis

ANSWER: c. Chest tube placement.

The diagnostic thoracentesis is consistent with an empyema with pH <7.2, glucose < 60, and bacteria seen on gram stain. A chest tube should be placed to drain the effusion, obtain source control, and will lead to faster clinical improvement than just antibiotics alone.

Modifier Q31

An 80 year old male is intubated and mechanically ventilated after his pneumonia and sepsis progressed to ARDS. The ventilator is on assist control mode with RR 16, Tidal volume 480, Positive end expiratory pressure (PEEP) 5mmHg and FiO2 75%. ABG shows pH 7.32, pCO2 48, pO2 45. What is the next best step?

1. Increase PEEP
2. Increase Tidal volume
3. Increase Respiratory rate
4. Change to Pressure control ventilatory mode

ANSWER: a. increase PEEP.

In ARDS, the ALVEOLI study compared high PEEP vs low PEEP ventilation and found that high PEEP helped with oxygenation in ARDS though had no mortality difference. The increased PEEP can help recruit alveoli that are collapsed or flooded and also decrease cyclical atelectasis which can cause ventilator associated lung injury.

Modifier Q32

A 55 year old female with history of COPD presents to clinic with worsening dyspnea over the past months. She is only using an albuterol inhaler several times a week. Spirometry shows FEV1 55%, FVC 90% and FEV1/FVC 65% with no bronchodilator improvement. Chest imaging does not show any infiltrates, vitals are normal, lung exam with mild expiratory wheezing. He has no hospitalizations or major exacerbations. What is the best therapy to start (in addition to his albuterol inhaler)?

1. ICS
2. LABA-ICS
3. Long acting muscarinic antagonist
4. Long term oxygen therapy

ANSWER: c. long acting muscarinic antagonist.

A daily tiotropium, for example, can be added to his regimen. If his symptoms progress, he would benefit from the addition of a LABA-ICS.

Modifier Q33

A 65 year old man presents with dyspnea after a coronary artery bypass procedure 3 months ago. He has worsened dyspnea when supine. SpO2 upright is 97% and supine is 90%. Vitals are otherwise normal and labs are normal. Jugular venous pressure is at 5cm H2O and lung exam has dullness to percussion in the left base. Spirometry is consistent with a restrictive picture, FEV1/FVC ratio of 1.00. What is the cause of the dyspnea?

1. Heart failure exacerbation
2. Pulmonary embolism
3. Pneumonia
4. Paralyzed left hemidiaphragm

ANSWER: d. Paralyzed left hemidiaphragm.

During the patient’s cardiac surgery, the left phrenic nerve was damaged. The SpO2 and dyspnea is worse when supine because the abdominal viscera are displaced cephalad when supine and further worsen the restrictive defect.

Modifier Q34

An immunocompetent 25 year old patient is intubated with ARDS secondary to necrotizing alcoholic pancreatitis. He is started on appropriate broad anti-bacterial agents and fluid resuscitation. His sputum culture has light growth candida species and chest XR shows persistent bilateral infiltrates. What is the next best step in management of the sputum culture findings?

1. Itraconazole
2. Fluconazole
3. Anidulafungin
4. No antifungal drug treatment

ANSWER: d. No antifungal drug treatment.

In an immunocompetent adult patient with Candida from respiratory sputum sample, there is no need to treat the candida because it is likely colonization. Candida pneumonia is usually due to hematogenous spread from disseminated candidiasis from a separate source, rather than aspiration of oropharyngeal secretions.

Modifier Q35

A patient with status asthmaticus is intubated after symptoms were refractory to appropriate initial management. In addition to continuing the albuterol and steroids and proper sedation, which of the following treatments is most appropriate at this time?

1. Ketamine
2. Benadryl
3. Naproxen
4. Sodium bicarbonate

ANSWER: a. Ketamine.

Ketamine is bronchodilator and can be used in status asthmaticus. The other options have no role in acute asthma management.

Modifier Q36

A patient is interested in smoking cessation. Which of the following considerations need to be assessed before starting varencline?

1. Cancer history
2. Pulmonary hypertension
3. Liver disease
4. Kidney disease

ANSWER: d. Kidney disease

Varenicline dosage will need to be adjusted in severe renal dysfunction. The other answers are not contraindications. There is no dose adjustment required for patients with liver disease.

Modifier Q37

A young patient with no medical history is involved in a motor vehicle crash leading to a humerus fracture. He is brought to the OR and is transfused 2 liters of normal saline and 1 unit of packed RBC. He is extubated and brought to the PACU in stable condition. 3 hours later he has acute onset of hypoxemia requiring re-intubation. Chest XR has new patchy bilateral alveolar infiltrates. What is the cause of his respiratory failure?

1. Fat embolism
2. Congestive heart failure and volume overload
3. Transfusion-associated acute lung injury
4. Myocardial infarction

ANSWER: c. Transfusion associated acute lung injury.

Hallmarks for TRALI include new acute lung injury, hypoxemia with PaO2/FiO2 <300, bilateral pulm infiltrates. Onset of the lung injury was within 6 hours of the transfusion.

Modifier Q38

A patient is hospitalized with a community acquired pneumonia and treated with 3 days of ceftriaxone and azithromycin before being discharged with a fluoroquinolone to finish a 7 day course. She follows up in pulmonology clinic 6 weeks later and has full resolution of symptoms and normal vitals. What is the next best step at this time?

1. Chest XR
2. Chest CT
3. C-reactive protein and erythrocyte sedimentation rate levels
4. Pro-calcitonin testing
5. No additional testing

ANSWER: e. No additional testing

Modifier Q39

A patient with recent diagnosis of right sided breast cancer was treated with lumpectomy and radiation which was completed 8 weeks ago. She presents with dry cough, pleuritic pain, dyspnea and was treated for 7 days with moxifloxacin without improvement. Chest XR demonstrates a right lower lobe opacity. Her vitals are normal and labs are unremarkable except for WBC 11k. TTE is normal. What is the best next step in management?

1. Lasix
2. Ceftriaxone and Azithromycin
3. Prednisone
4. Fluconazole

ANSWER: c. Prednisone.

The patient has acute radiation pneumonitis, which often presents with pneumonia-like symptoms that are not responding to antibiotics, in the setting of radiation treatment within the past 1-3 months. She will require treatment with steroids for 2 weeks with a gradual taper over potentially several months.

Modifier Q40

A 20 year old female is on maximal asthma therapy with good compliance yet has had repeated episodes of asthma exacerbations. She has been intubated twice and has needed many courses of prednisone bursts. The attacks are triggered by stress, dust, cigarette smoke, exercise without seasonal variations. Her vitals are normal and she says she feels throat tightness occasionally. Spirometry demonstrates FEV1 95% predicted and FVC 100% predicted with FEV1/FVC 0.95. Lung volumes and diffusion capacity are normal. Chest XR is normal.

What is the best next step?

1. Chest CT
2. Transthoracic echocardiogram
3. Repeat spirometry with a flow volume loop
4. Start an anxiolytic

ANSWER: c. Repeat spirometry with a flow volume loop.

The patient has features consistent with vocal cord dysfunction, and likely has paradoxical vocal fold motion (PVFM). PVFM is often misdiagnosed as asthma and should be considered when patients are unresponsive to maximal asthma therapy. PVFM can be detected on flow-volume curves with a flattening of the inspiratory curve suggesting variable extrathoracic airway obstruction. Diagnosis is confirmed with laryngoscopy.

Modifier Q41

A patient with asthma and chronic rhinosinusitis has an asthma exacerbation after starting over the counter aspirin and ibuprofen for sinus headaches and congestion. Within 2 hours he started having facial flushing, rhinorrhea, conjunctival injection and worsening dyspnea. He was treated with prednisone and albuterol nebulizers with improvement. What else would benefit this patient in addition to discontinuation of the NSAIDs and Aspirin?

1. Azithromycin
2. Tiotropium
3. Anti-IgE therapy
4. Montelukast

ANSWER: d. Montelukast.

The patient has a presentation consistent with aspirin-exacerbated respiratory disease (AERD). Classically the Samter’s triad for AERD includes chronic rhinosinusitis, asthma, and aspirin (or NSAID) sensitivity. NSAIDs or ASA can cause asthma exacerbation, profuse rhinorrhea, conjunctival injection, and facial flushing within 3 hours. The reaction is NOT an IgE mediated allergic response; the mechanism is thought to be a leukotriene overproduction.

Modifier Q42

In a patient with hemoptysis, how should they be positioned?

1. Bad lung down
2. Bad lung up
3. Supine
4. Prone
5. 60 degrees head of the bed incline

ANSWER: Bad lung down

The blood will move dependently and positioning “bad lung down” will prevent blood from compromising the unaffected lung.

Modifier Q43

A 50 year old female presents with a left sided pneumonia and is intubated for hypoxic respiratory failure. Capnography waveform is normal, colorimetric end tidal CO2 device has a color change, the endotracheal tube is at 25cm at the teeth. Pre intubation SpO2 is 82% and post intubation SpO2 is 85% on 100% FiO2 and PEEP 10. There are reduced lung sounds on the left. What is the next best step?

1. Needle decompression of the left chest
2. Increase PEEP
3. Increase respiratory rate
4. Retract the endotracheal tube by 3-5cm
5. Broaden antibiotics

ANSWER: d. Retract the endotracheal tube by 3-5cm.

The right mainstem bronchus was intubated leading to collapse of the left lung. A chest XRay can confirm the tube position.

Modifier Q44

Which of the following is the best method for diagnosis of idiopathic pulmonary fibrosis?

1. Bronchoscopy with transbronchial biopsy
2. Transesophageal echocardiogram
3. High resolution chest CT scan
4. Chest CT scan with IV contrast
5. Pulmonary function testing

ANSWER: c. High resolution chest CT scan

High resolution chest CT scans use thin slices (often <1.5mm) to increase the spatial resolution for identifying pulmonary parenchymal and small airways pathology. It is the preferred imaging modality for idiopathic pulmonary fibrosis.

Modifier Q45

Pulmonary involvement can occur in c-antineutrophil cytoplasmic antibody mediated diseases. Which of the following is a c-ANCA associated disease?

1. Granulomatosis with polyangiitis
2. Rheumatoid arthritis
3. Sjogren’s disease
4. Primary sclerosing cholangitis
5. Polyarteritis nodosa

ANSWER: a. Granulomatosis with polyangiitis

Modifier Q46

The patient has a peripherally located spiculated nodule and a right sided pleural effusion. A thoracentesis is performed and 20ml are sent with exudative fluid studies and negative cytology. What is the next best step for diagnosis?

1. Sputum cultures
2. Bronchoscopy with lavage
3. Repeat thoracentesis up to three times with cytology
4. High resolution CT scan
5. Follow-up in 3-6 months

ANSWER: c. Repeat thoracentesis up to three times with cytology.

The first pleural fluid cytologic study has a ~60% sensitivity for detecting malignant cells. With 3 separate thoracenteses, the sensitivity improves to 90% in malignant effusions.

Modifier Q47

A patient presents with acute shortness of breath and a cardiac point of care ultrasound demonstrates right ventricular enlargement with akinesis of the mid free wall and a more dynamic RV apex. Lung POCUS demonstrates normal lung sliding and A lines. What pathology do these findings suggest?

1. Pneumothorax
2. Pulmonary embolism
3. Congestive heart failure
4. Pneumonia
5. Acute respiratory distress syndrome (ARDS)

ANSWER: b. Pulmonary embolism.

The cardiac POCUS findings described are termed “McConnell’s sign” and is a finding associated with sudden right heart pressure and volume overload in the setting of a massive pulmonary embolism. The lung POCUS findings are “normal” because pulmonary embolism should not affect the lung sliding or parenchyma.

Modifier Q48

In lung point of care ultrasound, an anechoic space located superior to the diaphragm indicates which of the following:

1. Pneumothorax
2. Pulmonary edema
3. Consolidation
4. Emphysema
5. Pleural effusion

ANSWER: e. Pleural effusion

As a reminder, on ultrasound, fluid is black (anechoic) and denser structures are bright.

Modifier Q49

A thoracentesis is performed and the fluid studies demonstrates Protein 3.5, Glucose 12, LDH 1250, cell count 1000 /uL with 88% lymphocytes. What is the most likely etiology of the pleural effusion?

1. Empyema
2. Pulmonary embolism
3. Pancreatitis
4. Rheumatoid pleurisy
5. Congestive heart failure
6. Nephrotic syndrome

ANSWER: d. Rheumatoid pleurisy.

The very low glucose < 30 with extremely high LDH > 1000 is typically only found in a bacterial empyema or rheumatoid pleurisy. The cell count differentiates these two: low cell count with lymphocyte predominance indicates rheumatoid pleurisy and high cell count with neutrophil predominance indicates empyema.

Modifier Q50

A patient with a severe asthma exacerbation has refractory symptoms despite albuterol, IV methylprednisolone, and escalating oxygen support. Which of the following would be most appropriate at this time?

1. Epinephrine
2. Budesonide
3. IV Phosphate
4. IV Immunoglobulin
5. Hydroxyzine

ANSWER: a. Epinephrine

Other therapies used in refractory asthma exacerbations include IV epinephrine infusion, ketamine, helium-oxygen mixture, continuous albuterol nebulizers.
