# Supplementary material for: Pulmonopoly: A Game-Based Approach to Teach and Reinforce Basic Concepts of Pulmonary Medicine to Medical Students
Source: MedEdPORTAL. 2025 Feb 21;21:11493. doi: 10.15766/mep_2374-8265.11493 (PMC11842520; doi:10.15766/mep_2374-8265.11493)
Supplement: Supplementary file 1 — Pulmonopoly Board.pdfQuestion Cards.docxProperty Cards, Modifier Cards, and Player Pieces.pdfQuestion and Answer Key.docxGame Rules.docxPre- and Postintervention Surveys.docx [file mep_2374-8265.11493-s001.zip › E. Game Rules.docx]

**Pulmonopoly Game Rules!**

**Setup**

- 4 teams (can have one or more players per team) and a game moderator.
- Each team should choose a player piece and place it on “Lost Your Inhaler”
- Each team will roll two dice. The highest number will determine the team that goes first. Then, each team will take turns in a clockwise direction.
- Place each of the Question decks on their appropriate positions (Anatomy/Pharmacology, Physiology, Pathophysiology, Modifier)
- Place the Property Cards on the side. These will be distributed by the Moderator as the game progresses.
- Expected time to complete the game: 45-60 minutes

**Gameplay**

- Teams start on “Lost Your Inhaler” and roll the two dice to advance around the board.
- When a team lands on a Property space, there will be a question category listed (Anatomy/Pharmacology, Physiology, Pathophysiology). Draw a card from the corresponding Question Bank deck.
  - Read the question aloud for all teams to hear. The team who drew the card will have one minute to answer the question.
  - The moderator will have the question bank and answers. After the team answers, the moderator will read the correct answer and explanation (if available) to the teams. They also may add salient teaching points.
  - For educational purposes, moderators will encourage discussion of the answers with all players prior to announcing the correct answer and explanation. Other teams cannot “steal” a property card by answering the question out of turn.
  - The session instructor is available to settle disputes!
  - If the team answers correctly, they will receive the corresponding Property Card.
  - If the team does not answer the question correctly, there will be no penalty and the team will not be awarded the card in play. After discussion, the question card is discarded and the game will proceed with the next team’s turn.
- When a team lands on a Modifier space, they will draw a Modifier Question from the corresponding deck. These cards have more advanced questions related to pulmonary medicine.
  - As above, the team who drew the card will read it aloud and have one minute to answer, and the moderator will read the correct answer and explanation afterwards.
  - If the team answers correctly, they will receive a gray Modifier Card.
  - If the team does not answer the Modifier question correctly, there will be no penalty and the team will not be awarded the Modifier card in play. After discussion, the Modifier question card is discarded and the game will proceed with the next team’s turn.
  - As above, other teams cannot “steal” the card by answering the question. However, discussion of the questions and answers is encouraged.
- After a question is discussed, it is discarded. There are a surplus of questions such that a game can be played multiple times in a given session with the same group of learners.
- The turn ends after the dice roll and the subsequent action is completed.

**Using Modifier Cards**

- As the game progressed, teams will accumulate Modifier cards after answering correctly from the Modifier Question deck.
- The Modifier cards have different functions that can be triggered by using one, two, or three Modifier cards at a time.

1. Using one Modifier card will allow the player to increase or decrease the dice roll number by up to two (for example, changing a dice roll of 6 into an 8), thus changing the space the team lands on.
2. Two Modifier cards can be used to force a property trade with another team. The other team can try to negotiate but cannot refuse the trade.
3. Three Modifier cards can be used to obtain any property at the end of their turn.

- Once used, the Modifier cards are discarded.

**Victory Conditions**

- To win the game, a team must gain one Property Set. A Property Set is all the properties within a single color-coordinated neighborhood.
- For a longer game experience, you may instead change the win condition to gaining two Property Sets.
