# Supplementary material for: Pulmonopoly: A Game-Based Approach to Teach and Reinforce Basic Concepts of Pulmonary Medicine to Medical Students
Source: MedEdPORTAL. 2025 Feb 21;21:11493. doi: 10.15766/mep_2374-8265.11493 (PMC11842520; doi:10.15766/mep_2374-8265.11493)

Chronic Cough Canyon	Chronic Cough Canyon	Chronic Cough Canyon	Chronic Cough Canyon
The Sputum Sea	The Sputum Sea	The Sputum Sea	The Sputum Sea
Ventilator Vista	Ventilator Vista	Ventilator Vista	Ventilator Vista
Tracheal Terrace	Tracheal Terrace	Tracheal Terrace	Tracheal Terrace

The Bronchs	The Bronchs	The Bronchs	The Bronchs
The Vagus Strip	The Vagus Strip	The Vagus Strip	The Vagus Strip
Mucolytic Mountain	Mucolytic Mountain	Mucolytic Mountain	Mucolytic Mountain
Dyspnea Drive	Dyspnea Drive	Dyspnea Drive	Dyspnea Drive

**Auscultation Alley**	**Auscultation Alley**	**Auscultation Alley**	**Auscultation Alley**
**Diaphragmatic Diner**	**Diaphragmatic Diner**	**Diaphragmatic Diner**	**Diaphragmatic Diner**
**Obstruction Overpass**	**Obstruction Overpass**	**Obstruction Overpass**	**Obstruction Overpass**
**Bronchial Boulevard**	**Bronchial Boulevard**	**Bronchial Boulevard**	**Bronchial Boulevard**

**Phrenic Parkway**	**Phrenic Parkway**	**Phrenic Parkway**	**Phrenic Parkway**
**Spacer Station**	**Spacer Station**	**Spacer Station**	**Spacer Station**
**Alveolar Avenue**	**Alveolar Avenue**	**Alveolar Avenue**	**Alveolar Avenue**
**Tracheal Terrace**	**Tracheal Terrace**	**Tracheal Terrace**	**Tracheal Terrace**

Inhaler Island	Inhaler Island	Inhaler Island	Inhaler Island
Pleural Place	Pleural Place	Pleural Place	Pleural Place
Lake LABA	Lake LABA	Lake LABA	Lake LABA
Pneumonia Pnational Park	Pneumonia Pnational Park	Pneumonia Pnational Park	Pneumonia Pnational Park

CPET Coliseum	CPET Coliseum	CPET Coliseum	CPET Coliseum
PFT Palace	PFT Palace	PFT Palace	PFT Palace
Modifier Card	Modifier Card	Modifier Card	Modifier Card
Modifier Card	Modifier Card	Modifier Card	Modifier Card

**Modifier Card**	**Modifier Card**	**Modifier Card**	**Modifier Card**
**Modifier Card**	**Modifier Card**	**Modifier Card**	**Modifier Card**
**Modifier Card**	**Modifier Card**	**Modifier Card**	**Modifier Card**
**Modifier Card**	**Modifier Card**	**Modifier Card**	**Modifier Card**

Modifier Card	Modifier Card	Modifier Card	Modifier Card
Modifier Card	Modifier Card	Modifier Card	Modifier Card
Modifier Card	Modifier Card	Modifier Card	Modifier Card
Modifier Card	Modifier Card	Modifier Card	Modifier Card

Player Pieces

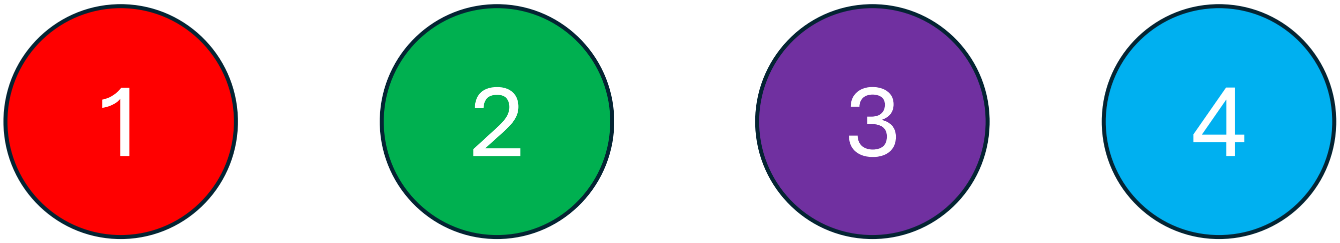

Supplement: Supplementary file 1 — Pulmonopoly Board.pdfQuestion Cards.docxProperty Cards, Modifier Cards, and Player Pieces.pdfQuestion and Answer Key.docxGame Rules.docxPre- and Postintervention Surveys.docx [file mep_2374-8265.11493-s001.zip › C. Property Cards, Modifier Cards, and Player Pieces.pdf]
